# Petasis Sequence Reactions for the Scaffold-Diverse Synthesis of Bioactive Polycyclic Small Molecules

**DOI:** 10.1021/acsomega.2c06585

**Published:** 2022-12-16

**Authors:** Amrutha K. Avathan Veettil, Jan-Lukas Kirchhoff, Lukas Brieger, Carsten Strohmann, Peng Wu

**Affiliations:** † Chemical Genomics Centre, Max Planck Institute of Molecular Physiology, Dortmund 44227, Germany; ‡ Department of Chemical Biology, Max Planck Institute of Molecular Physiology, Dortmund 44227, Germany; § Faculty of Chemistry and Chemical Biology, TU Dortmund University, Dortmund 44227, Germany

## Abstract

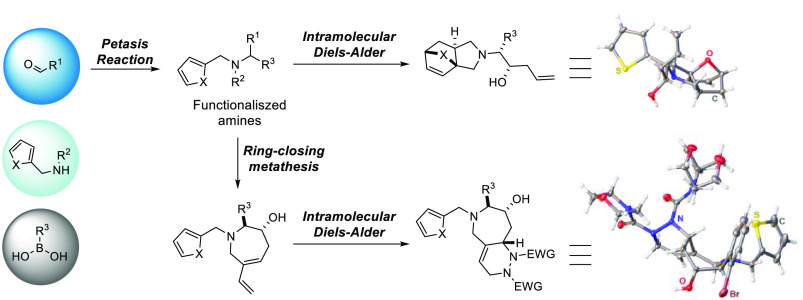

The multicomponent Petasis reaction is a versatile method to access functionalized amines. The combination of Petasis reaction with subsequent ring-closing reactions is a powerful strategy to build novel polycyclic scaffolds. In this study, we report the generation of a diverse set of small molecules with polycyclic scaffolds featuring a high content of sp^3^-hybridized carbon atoms and multiple stereogenic centers by employing three-component Petasis reaction (3C-PR)—Intramolecular Diels–Alder (IMDA) and 3C-PR—ring-closing metathesis (RCM)—IMDA sequence reactions. This work demonstrates the wide substrate tolerance and broad applicability to access unexplored polycyclic scaffolds of biological interest using Petasis sequence reactions.

## Introduction

Small molecules are valuable therapeutics for treating human diseases and useful tools in probing biological functions of proteins, human physiology, and numerous cellular activities. One limitation in the current field of small-molecule research lies in the limited coverage of “chemical space” in synthetic molecules and commercially available screening libraries, which represent merely a tiny fraction of all possible structural complexity. The vastness of unexplored chemical space spurred chemists to develop efficient approaches to populate the current reservoir of small molecules and compound libraries with compounds covering bioactivity-related chemical space.^1−3^ One such approach is to build compound collections with high structural diversity and coverage on unexplored chemical space.^4−9^ A wide range of synthetic methods have been applied for the synthesis of sp^3^-rich polycyclic bioactive small molecules.^10−12^ Recent synthetic examples include the solid-phase synthesis of DNA-tagged heterocycles,^13^ rhodium-catalyzed intramolecular annulations,^14^ and visible-light-photocatalyzed synthesis of N-heterospirocyles.^15^ Compounds that are structurally complex are likely to interact with biomacromolecules with different selectivity and specificity profiles.^7,16^ An efficient synthetic method to access compounds of diverse structures is the employment of multicomponent reactions (MCRs), which are highly relevant to medicinal chemistry and drug discovery as MCRs enable the construction of diverse heterocyclic scaffolds with a high degree of complexity and stereoselectivity.^17,18^

**Figure 1:**
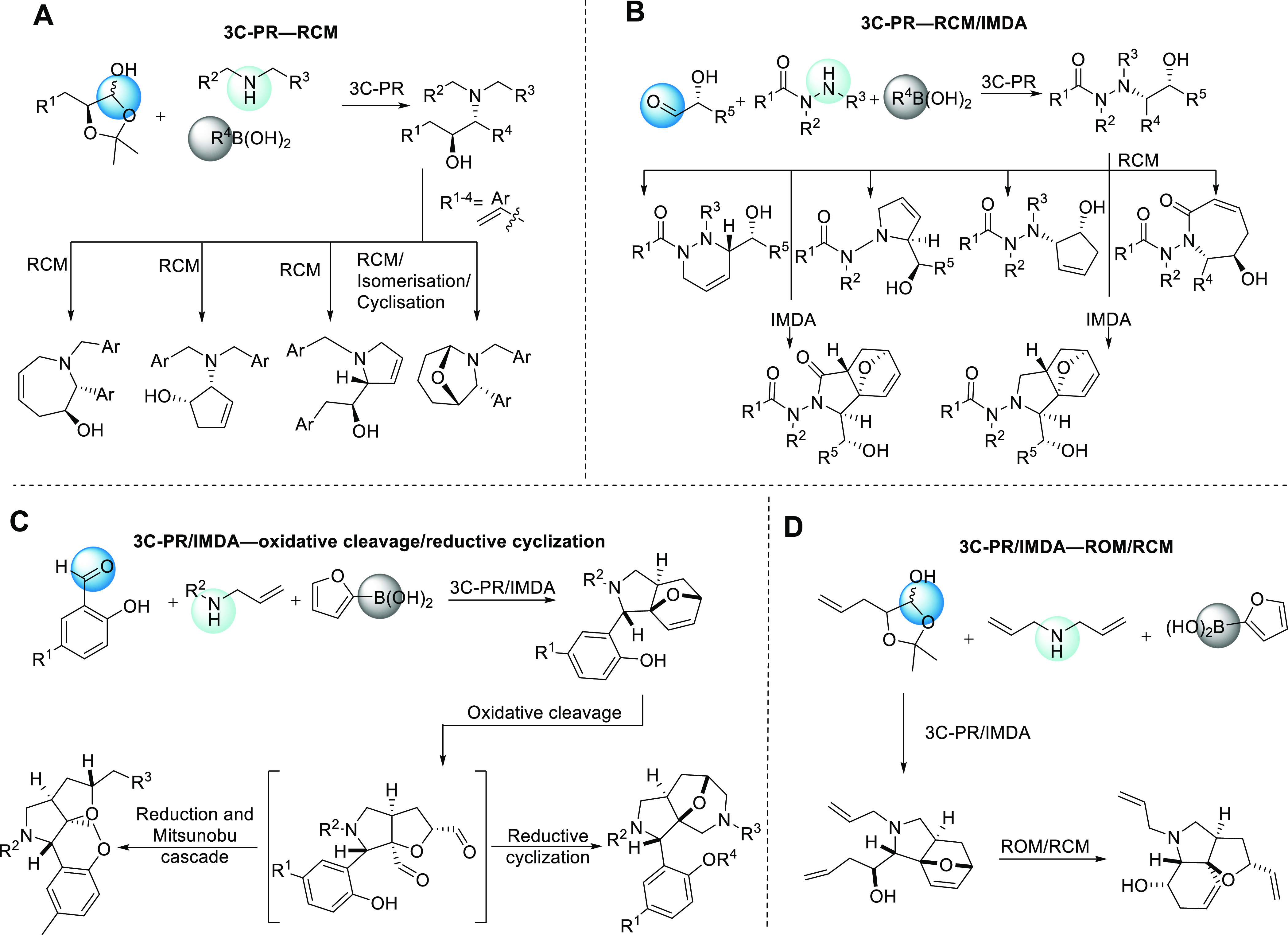
Representative examples of multicomponent Petasis sequence reactions for the synthesis of structurally diverse scaffolds. (A) 3C-PR—RCM sequence reactions; (B) 3C-CR—RCM/IMDA sequence reactions; (C) 3C-PR/IMDA—oxidative cleavage/reductive cyclization sequence reactions; (D)3C-PR/IMDA—ROM/RCM sequence reactions. 3C-PR: three-component Petasis reaction; IMDA: intramolecular Diels–Alder reaction; RCM: ring-closing metathesis reaction; ROM: ring-opening metathesis reaction.

**Figure 2:**
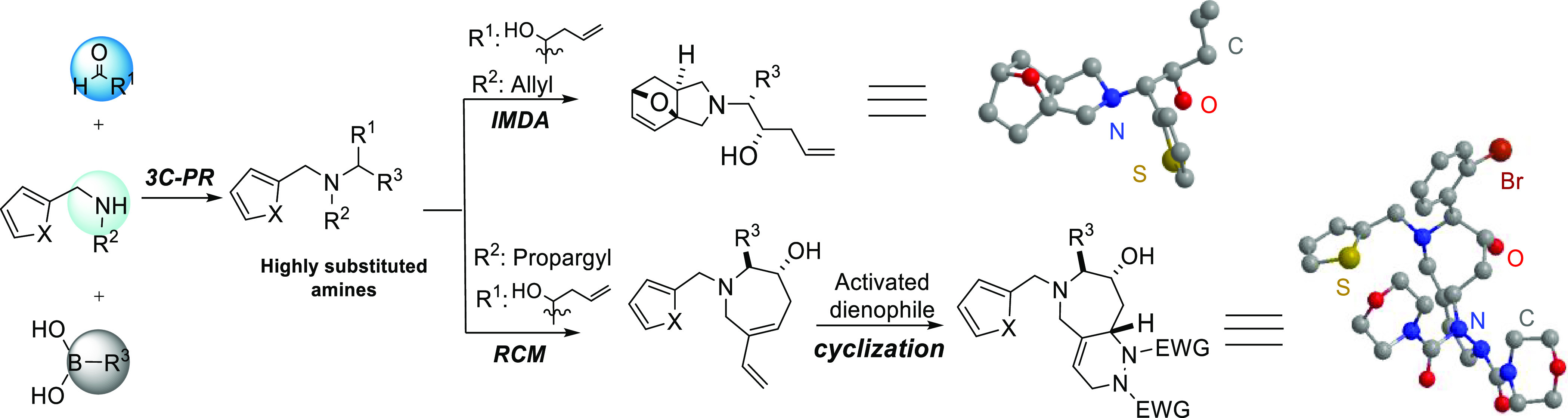
Petasis sequence reactions reported in this study: A three-component Petasis reaction (3C-PR)/intramolecular Diels–Alder reaction (IMDA) sequence led to the generation of a hydroepoxyisoindole scaffold. A 3C-PR—RCM—Intermolecular cyclization led to the generation of a hydroazepine-fused bicyclic scaffold.

Multicomponent Petasis reaction (PR) enables the preparation of highly functionalized amines from a primary or secondary amine, a boronic acid, and a carbonyl component.^19−22^ PR products are amenable precursors for sequence reactions and are compatible with the combination of various secondary transformations to yield compounds with polycyclic scaffolds, such as PR—ring-closing metathesis (RCM) (Figure 1A), PR—intramolecular Diels–Alder (IMDA) (Figure 1B), and PR/IMDA in conjunction with other cyclization reactions (Figure 1C and 1D). Examples reported in the past decade demonstrated the great value of PR in synthesizing structurally diverse small molecules, natural products, and molecular libraries.^22−32^ In this context, we are continuing our efforts aiming to develop efficient methods for the construction of new polycyclic scaffolds for small molecule discovery. In this study, we report two synthetic sequences involving the multicomponent PR to form synthetically tractable small molecules of diverse structural complexity that are evaluated as bioactive compounds in early drug discovery stages (Figure 2).

## Results and Discussion

**Scheme 1:**
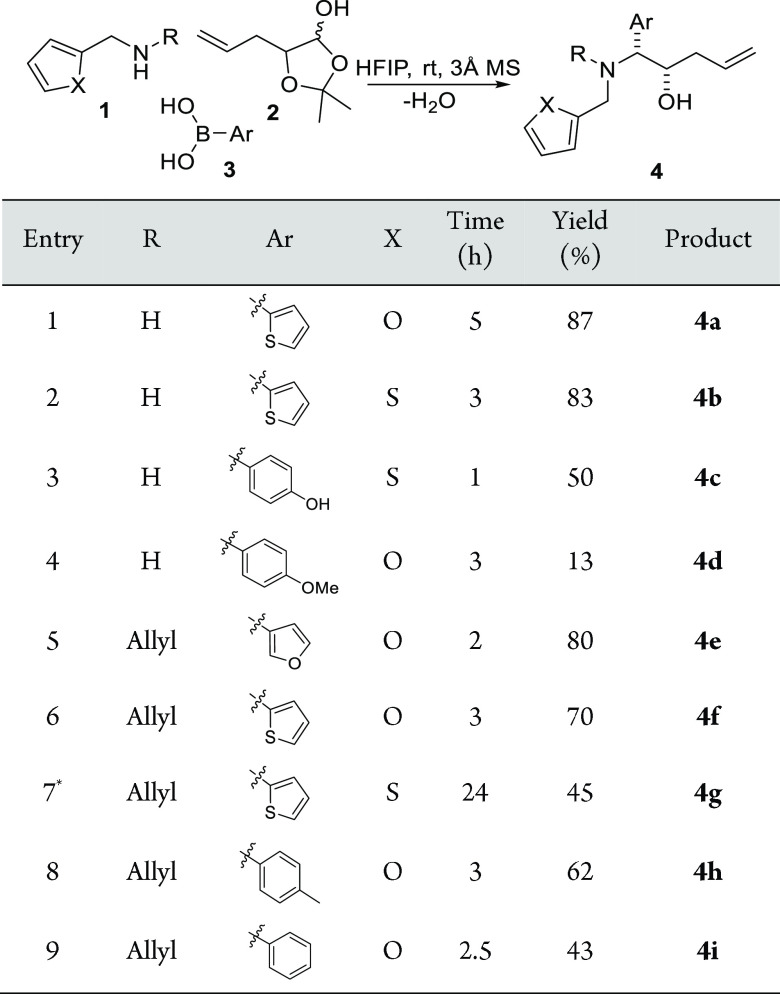
Petasis Reaction to Form Allylated Tertiary Amines **4** Reaction conditions: 1 (1 equiv), 2 (1.2 equiv), 3 (1.2 equiv) The reaction was monitored throughout by LC-MS and TLC. *N-allylation of **4b**.

We started our investigation by performing the 3C-PR to yield Petasis products with suitable appendages for subsequent polycyclic scaffold-generating reactions. Initial Petasis reactions were performed with primary amines (furfurylamine **1a** and 2-thiophene methyl amine **1b**), α-hydroxy aldehyde masked as the corresponding lactol (5-allyl-2,2-dimethyl-1,3-dioxolan-4-ol **2**), and various commercially available arylboronic acids **3** in an optimized reaction condition with hexafluoroisopropanol (HFIP) as solvent (Scheme 1).^33^ The use of primary aromatic amines **1a** and **1b** gave the corresponding Petasis products **4a**–**4d** in varied yields (13–87%) depending on the boronic acids used. Boronic acids substituted by electro-withdrawing groups, such as (4-nitrophenyl)boronic acid and (4-(trifluoromethyl)phenyl)boronic acid, failed to give the Petasis products in isolatable yields. A following *N*-alkylated step using allyl bromide could afford the corresponding allylation compounds. Alternatively, *N*-allyl-furfurylamine and *N*-allyl-thiophenemethylamine were used as the amine component to afford the Petasis products **4e**–**4i** in overall moderate to good yield (43–83%).

**Scheme 2:**
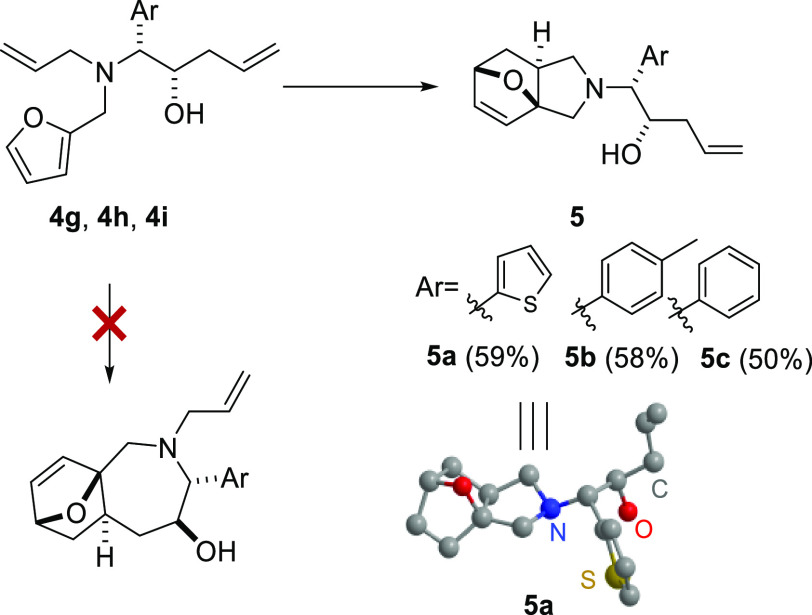
IMDA Products **5** from Petasis Products **4** Reaction conditions: toluene (0.5–0.4 M), reflux at 110 °C, 6 h.

Preliminary cyclization was performed based on the Petasis products **4e**–**4i** that contained the olefin appendages for an IMDA reaction. We envisioned that two cyclic scaffolds, epoxyisoindole and epoxybenzoazepine, would be formed, while after trying under different cyclization conditions, only the formation of the epoxyisoindole scaffold was observed. The furan-2-ylmethyl substituted Petasis products **4f**, **4h**, and **4i** gave the epoxyisoindoles **5a**–**5c** in 50–59% yields (Scheme 2). The thiophen-2-ylmethyl substituted Petasis product **4g** failed to cyclize under the refluxing condition even with a prolonged duration. All products were obtained as a single diastereomer. The *exo-*product of the IMDA reaction was confirmed by single-crystal X-ray analysis of compound **5a**.

**Scheme 3:**
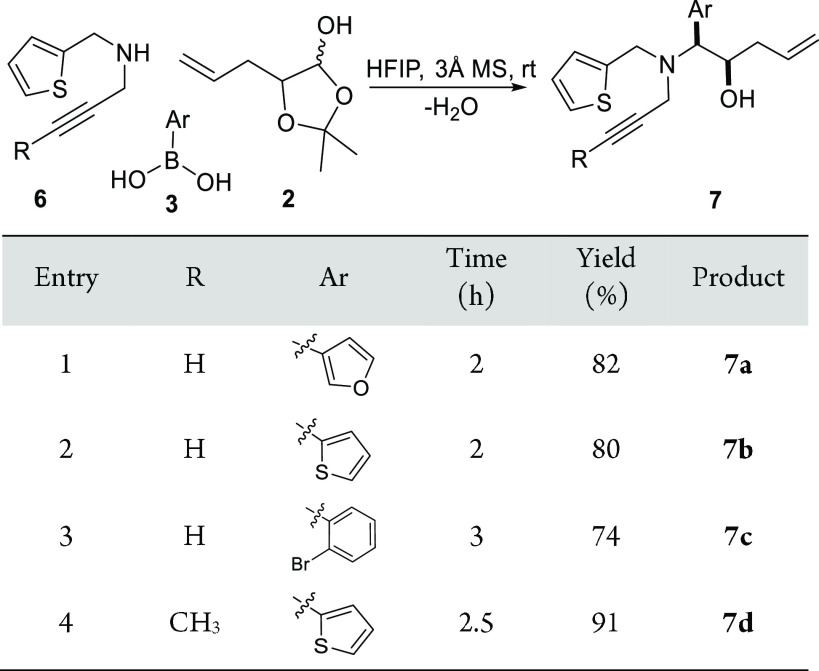
Petasis Reaction Accessing Tertiary Amines with Allyl and Propargyl Appendages Reagents and conditions: Amine (1.0 equiv), boronic acid (1.2 equiv), aldehyde (1.2 equiv), in HFIP at rt with 3 Å molecular sieves.

Given the PR—IMDA results using allylated Petasis products, we continued the synthesis of propargylated Petasis products **7** with the aim of obtaining new polycyclic scaffolds via IMDA or RCM between the positioned allyl- and propargyl substituents. *N*-propargyl-thiophenemethylamine **6** was synthesized by reported procedures and used as the amine component.^34^ Using 5-allyl-2,2-dimethyl-1,3-dioxolan-4-ol **2** and arylboronic acids, the corresponding Petasis products **7a**–**7d** were obtained in yields up to 91% (Scheme 3). The subsequent IMDA reactions using **7a**–7**d** as the substrate did not lead to any IMDA cyclized products under refluxing conditions. Therefore, we switched our efforts in screening different ruthenium catalysts to perform the enyne metathesis reaction to generate cyclized scaffolds.

**Scheme 4:**
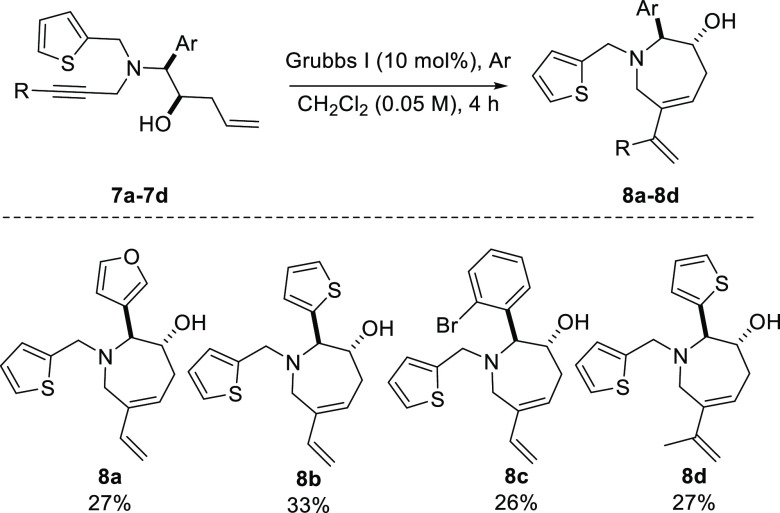
Enyne Metathesis of Petasis Products **7**

**Scheme 5:**
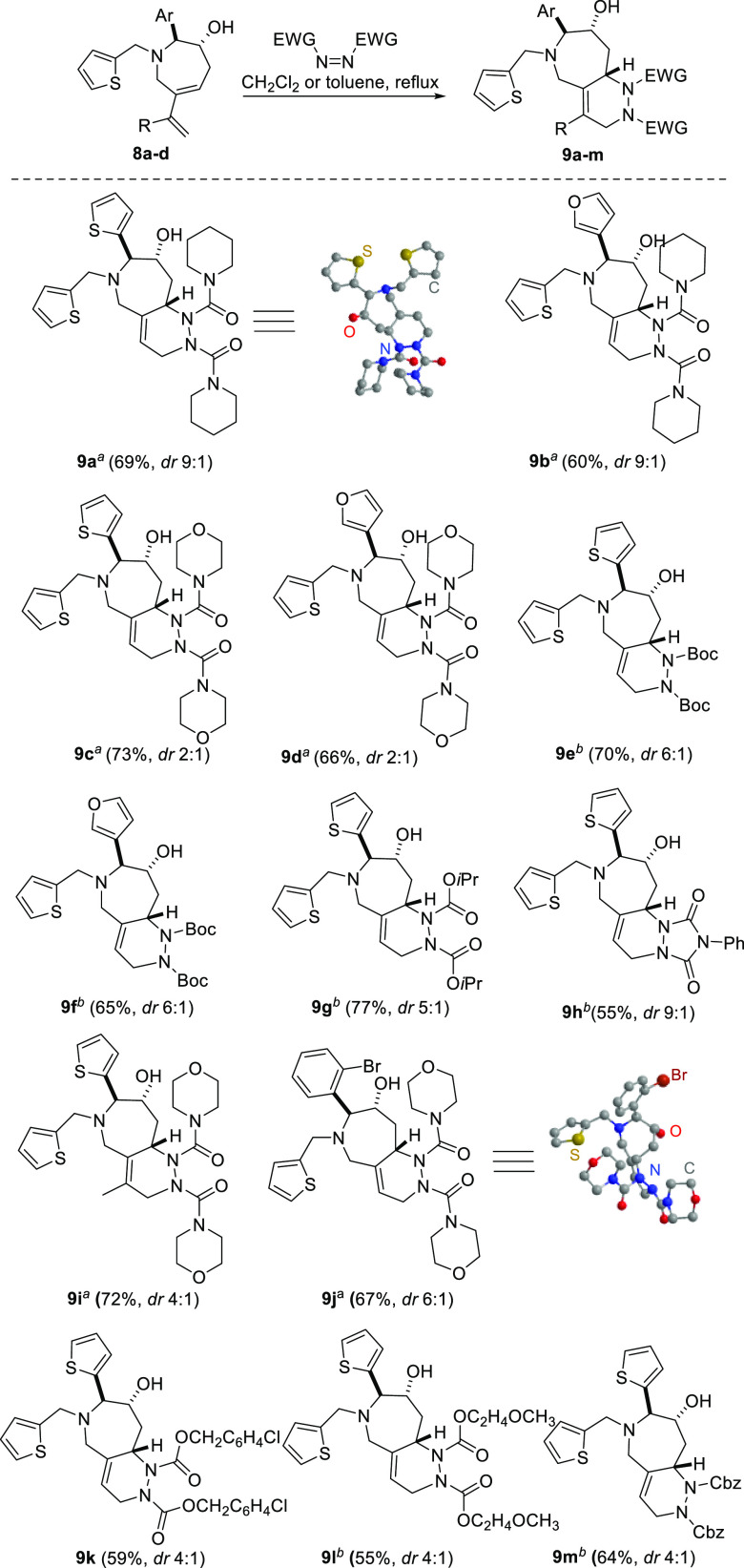
Secondary Cyclization: Intermolecular Diels–Alder Reaction Toluene reflux. CH_2_Cl_2_ reflux. *dr* determined by LCMS.

The use of Grubbs second generation catalyst resulted in the dimerization of Petasis product **7** with only a trace amount of expected cyclized product **8** (Scheme 4). The use of Grubbs first generation catalyst afforded the cyclized products **8a**–**8d** in improved yet poor yields, which were subjected to a further intermolecular Diels–Alder (DA) reaction in the presence of activated dienophiles to form bicyclic compounds **9** with a pyridazino[4,3-*c*]azepine scaffold (Scheme 5). A selection of the activated dienophiles including azodicarboxylic dimorpholide and 1,1′-(azodicarbonyl)dipiperidine gave the desired products in yields ranging from 55–77% as diastereomeric mixtures with the *endo*-product being the major diastereomer. The major *endo*-product was confirmed by single-crystal X-ray analysis of compounds **9a** and **9j**. The pyridazino[4,3-*c*]azepines **9** can be subject to further decoration, e.g., compounds **9e** and **9f** can be Boc-deprotected and functionalized on the resulting hydrazine amines.

**Figure 3:**
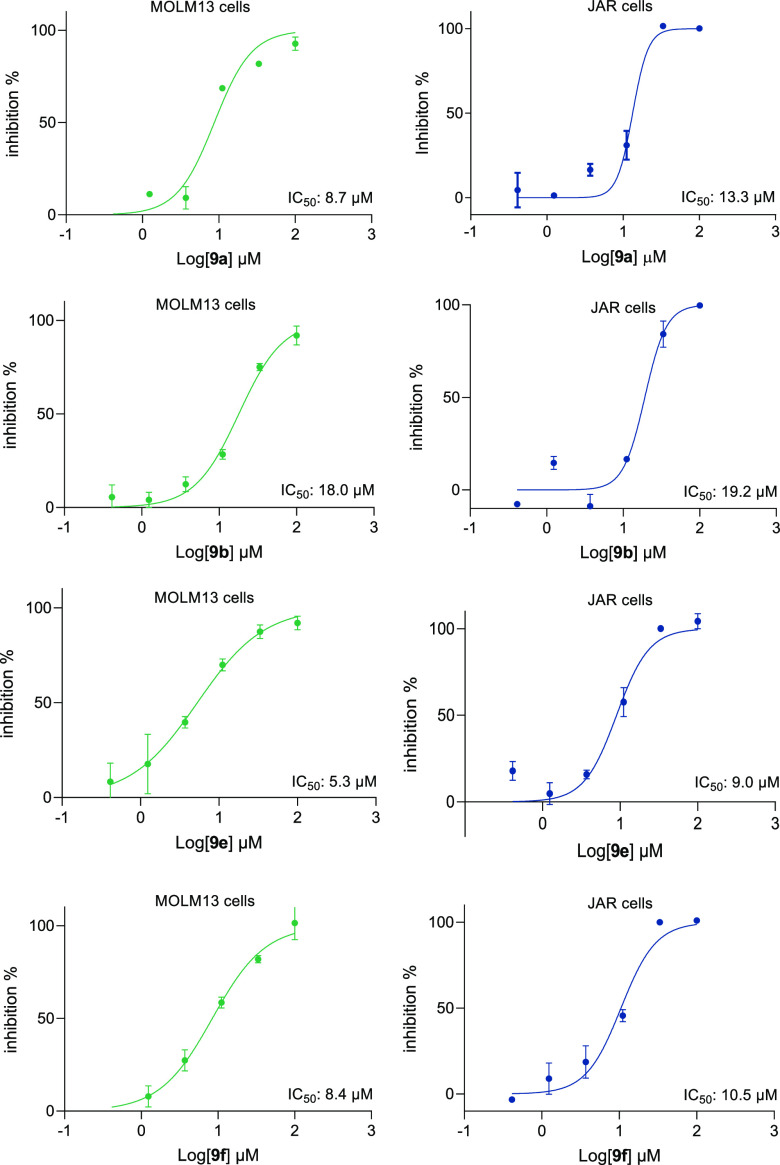
Antiproliferation activities of the obtained pyridazinoazepines **9a**, **9b**, **9e**, and **9f** against human cancer cells MOLM13 (left) and JAR (right), performed in duplicates.

To probe the medicinal chemistry properties, we calculated the molecular and absorption-distribution-metabolism-excretion (ADME) properties of synthesized compounds **5** and **9** (Tables S1 and S2), which revealed that many compounds showed favorable predicted drug-likeness properties. The biological relevance of the obtained pyridazino[4,3-*c*]azepines **9** was demonstrated by an antiproliferation assay, in which the tested compounds exhibited 40–90% inhibition against human acute myeloid leukemia cells MOLM-13 and the human choriocarcinoma cells JAR at a concentration of 10 μM. Compounds that showed at least 50% inhibition at 10 μM were further tested for their IC_50_ values, which revealed single-digit micromolar potency for selected compounds **9a**, **9b**, **9e**, and **9f** (e.g., azepine **9e**, IC_50_: 5.3 μM against MOLM-3 and 9.0 μM against JAR cells, respectively, Figure 3).

## Conclusion

We employed 3C-PR—IMDA and 3C-PR—RCM—IMDA sequences of robust and complexity-generating reactions to yield new small molecules with epoxyisoindole and pyridazinoazepine scaffolds. The sequence reactions were performed in straightforward and mild conditions with easily accessible substrates. The Petasis products and generated polycyclic compounds feature a high content of sp^3^-hybridized atoms and are amenable to be appended with other functional groups. The biological relevance of the obtained compounds has been tested in the antiproliferation assay against human cancer cell lines. The work demonstrated the broad applicability to access unexplored polycyclic scaffolds of biological interest using 3C-PR-involved sequence reactions.

## Experimental Section

### General Information

All commercially available solvents and reagents were purchased from Sigma-Aldrich, TCI Chemical, or Fisher Scientific and used without further purification. All reactions were monitored by thin layer chromatography (TLC) and an LC-MS Agilent 1260 II Infinity system equipped with a mass detector (column: InfinityLab Poroshell 120 EC-C18, 2.1 × 150, 2.7 μm). Appropriate gradient systems were applied by mixing H_2_O (+ 0.1% TFA) and acetonitrile (+ 0.1% TFA). Analytical thin-layer chromatography was carried out using Merck silica gel aluminum plates with F-254 indicator, visualized under UV light (at 254 nm), iodine stain, or dipping in potassium permanganate stain (1.5 g of KMnO_4_, 10 g of K_2_CO_3_, 1.25 mL of 10% aqueous NaOH solution, and 200 mL of water). Starting materials **1c**, **2**, **6a**, and **6b** were prepared following the known literature procedures and the characterization data were in agreement with the literature data. The products were purified by column chromatography over silica gel (Merck 60 particle size 0.040–0.063 mm). Solvents for chromatography were laboratory grade. All ^1^H and ^13^C NMR spectra were recorded on a Bruker DRX400 (400 MHz), DRX500 (500 MHz), DRX600 (600 MHz), and DRX700 (700 MHz) spectrometers in CDCl_3_ and (CD_3_)_2_SO. Data are reported in the following order: chemical shift in ppm; multiplicities are indicated s (singlet), d (doublet), t (triplet), q (quartet), dd (doublet of doublet), ddt (doublet of doublet of triplets), and m (multiplet). Coupling constants (*J*) are given in Hertz (Hz). High-resolution mass spectra were recorded on an LTQ Orbitrap mass spectrometer coupled to an Accela HPLC System (HPLC column: Hypersyl GOLD, 50 mm × 1 mm, 1.9 μm). Chemical yields refer to isolated pure substances.

### General Procedure for the Synthesis of Allylated Tertiary Amines **4a**–**4i**

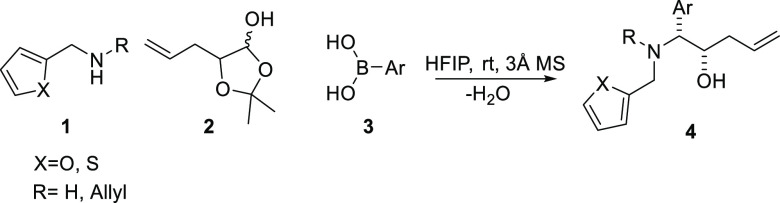



Primary amine/secondary amine (1 equiv), aldehyde (1.2 equiv), and boronic acid (1.2 equiv) were added simultaneously to hexafluoro-2-propanol (HFIP) (0.1 M) with 3 Å molecular sieves. The mixture was stirred at room temperature and monitored by LC-MS and TLC. The solvent then evaporated under reduced pressure. The resulting residue was purified by flash chromatography (4–10% ethyl acetate in petroleum ether).

#### (1*S*,2*R*)-1-((Furan-2-ylmethyl)amino)-1-(thiophen-2-yl)pent-4-en-2-ol (**4a**)

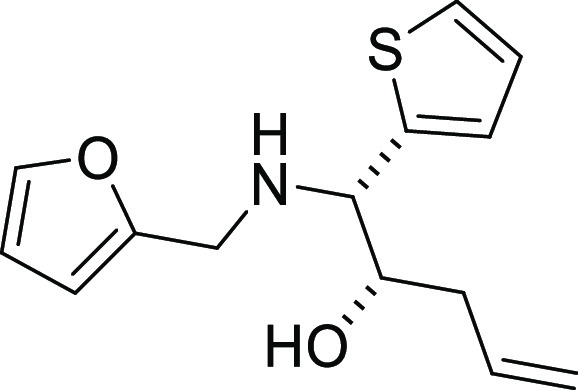



Using general procedure for the preparation of **4a**–**4i**, **4a** was obtained from furfurylamine **1a** (50 mg, 0.51 mmol), 5-allyl-2,2-dimethyl-1,3-dioxolan-4-ol **2** (97.74 mg, 0.62 mmol), and 2-thienylboronic acid **3a** (79.06 mg, 0.62 mmol) in HFIP (5 mL) for 5 h. Purified as a colorless oil. (117.96 mg, 87% yield). ^1^H NMR (500 MHz, CDCl_3_) δ 7.37 (dd, *J* = 1.8, 0.8 Hz, 1H), 7.31–7.29 (m, 1H), 7.02–6.99 (m, 2H), 6.31 (dd, *J* = 3.2, 1.9 Hz, 1H), 6.18 (m, 1H), 5.79 (m, 1H), 5.07 (m, 2H), 4.05 (d, *J* = 3.6 Hz, 1H), 3.96 (ddd, *J* = 8.5, 5.1, 3.7 Hz, 1H), 3.75 (dd, *J* = 73, 14.5 Hz, 2H), 2.11 (m, 2H). ^13^C NMR (126 MHz, CDCl3) δ 152.73, 142.29, 141.17, 134.62, 127.07, 126.49, 125.82, 118.01, 110.36, 107.93, 72.24, 61.22, 43.50, 37.88. HRMS-ESI (*m*/*z*): calculated for [M + H]^+^ C_14_H_18_NO_2_S, 264.1053; found, 264.1054.

#### (1*S*,2*R*)-1-(Thiophen-2-yl)-1-((thiophen-2-ylmethyl)amino)pent-4-en-2-ol (**4b**)

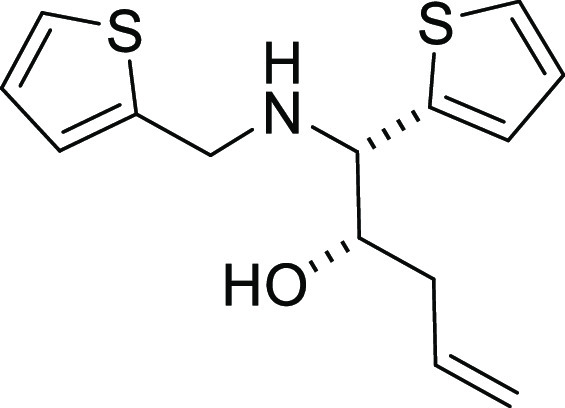



Using the general procedure for the preparation of **4a**–**4i**, **4b** was obtained from 2-thiophenemethylamine **1b** (50 mg, 0.44 mmol), 5-allyl-2,2-dimethyl-1,3-dioxolan-4-ol **2** (83.86 mg, 0.53 mmol), and 2-thienylboronic acid **3a** (67.83 mg, 0.53 mmol) in HFIP (4 mL) for 3 h. Purified as a colorless oil (102.46 mg, 83% yield). ^1^H NMR (600 MHz, CDCl_3_) δ 7.30 (m, 1H), 7.22 (dd, *J* = 5.4, 1.2 Hz, 1H), 7.01 (dd, *J* = 5.3, 3.6 Hz, 1H), 6.99–6.98 (m, 1H), 6.95 (dd, *J* = 4.8, 3.0 Hz, 1H), 6.90–6.89 (m, 1H), 5.84–5.77 (m, 1H), 5.05–5.09 (m, 2H), 4.09 (d, J = 3. Six Hz, 1H), 4.00 (dd, *J* = 13.8, 0.6 Hz, 1H), 3.95–3.92 (m, 1H), 3.88 (dd, *J* = 13.8, 0.6 Hz, 1H), 2.199–2.15 (m, 1H), 2.12–2,07 (1H, m).^13^C NMR (151 MHz, CDCl_3_) δ 143.25, 142.02, 134.75, 126.83, 126.76, 126.51, 125.56, 125.52, 124.85, 117.97, 72.70, 61.25, 45.74, 37.91. HRMS-ESI (*m*/*z*): calculated for [M + H]^+^ C_14_H_18_NOS_2_, 280.0824; found, 280.0825.

#### 4-((1*R*,2*R*)-2-Hydroxy-1-((thiophen-2-ylmethyl)amino)pent-4-en-1-yl)phenol (**4c**)

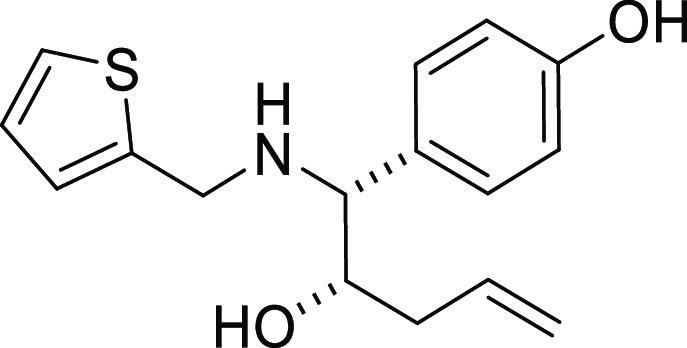



Using the general procedure for the preparation of **4a**–**4i**, **4c** was obtained from 2-thiophenemethylamine **1b** (50 mg, 0.44 mmol), 5-allyl-2,2-dimethyl-1,3-dioxolan-4-ol **2** (83.86 mg, 0.53 mmol), and 4-hydroxyphenylboronic acid **3b** (72.83 mg, 0.53 mmol) in HFIP (4 mL) for 1 h. Purified as a white powder (53 mg, 50% yield). ^1^H NMR (700 MHz, DMSO-*d*_6_) δ 9.19 (s, 1H), 7.36 (dd, *J* = 4.9, 1.4 Hz, 1H), 7.12–7.09 (m, 2H), 6.93 (dd, *J* = 4.9, 2.8 Hz, 1H), 6.85–6.83 (m, 1H), 6.72–6.70 (m, 2H), 5.81–5.75 (m, 1H), 4.96–4.93 (m, 2H), 4.56 (d, J = 5.4 Hz, 1H), 3.73 (d, *J* = 14 Hz, 1H), 3.65 (m, 1H), 3.58 (d, *J* = 14 Hz, 1H), 3.50 (d, *J* = 4.9 Hz, 1H), 2.55 (bs, 1H), 2.11–1.95 (m, 2H). ^13^C NMR (176 MHz, (CD_3_)_2_SO) δ 156.55, 145.57, 137.11, 131.13, 129.72, 127.01, 124.86, 124.76, 116.47, 115.16, 73.84, 65.49, 45.70, 37.98. HRMS-ESI (*m*/*z*): calculated for [M + H]^+^ C_16_H_20_NO_2_S, 290.1209; found, 290.1209.

#### (1*R*,2*R*)-1-((Furan-2-ylmethyl)amino)-1-(4-methoxyphenyl)pent-4-en-2-ol (**4d**)

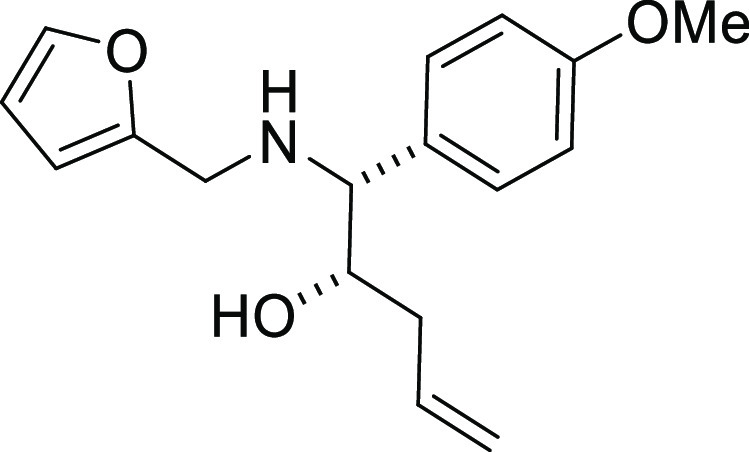



Using the general procedure for the preparation of **4a**–**4i**, **4d** was obtained from furfurylamine **1a** (70 mg, 0.72 mmol), 5-allyl-2,2-dimethyl-1,3-dioxolan-4-ol **2** (136.83 mg, 0.86 mmol), and 4-methoxyphenylboronic acid **3c** (131.43 mg, 0.86 mmol) in HFIP (5 mL) for 3 h. Purified as light-yellow oil (27 mg, 13% yield). ^1^H NMR (500 MHz, CDCl_3_) δ 7.36 (dd, *J* = 3.1, 1.9 Hz, 1H), 7.28–7.25 (m, 2H), 6.91–6.88 (m, 2H), 6.30 (dd, *J* = 3.1, 1.9 Hz, 1H), 6.12 (dd, *J* = 3.5, 0.5 Hz, 1H), 5.82–5.74 (m, 1H), 5.07–5.03 (m, 2H), 3.90–3.87 (m, 1H), 3.81 (s, 3H), 3.76 (d, *J* = 14.5 Hz, 1H), 3.69 (d, *J* = 2.5 Hz, 1H), 3.60 (d, *J* = 14.5 Hz, 1H), 2.12–1.97 (m, 2H). ^13^C NMR (126 MHz, CDCl_3_) δ 159.25, 153.26, 142.12, 135.15, 130.46, 129.72, 117.74, 113.94, 110.28, 107.52, 72.78, 64.86, 55.38, 43.65, 37.66. HRMS-ESI (*m*/*z*): calculated for [M + H]^+^ C_17_H_22_NO_3_, 288.1594; found, 288.1595.

#### (1*R*,2*R*)-1-(Allyl(furan-2-ylmethyl)amino)-1-(furan-3-yl)pent-4-en-2-ol (**4e**)

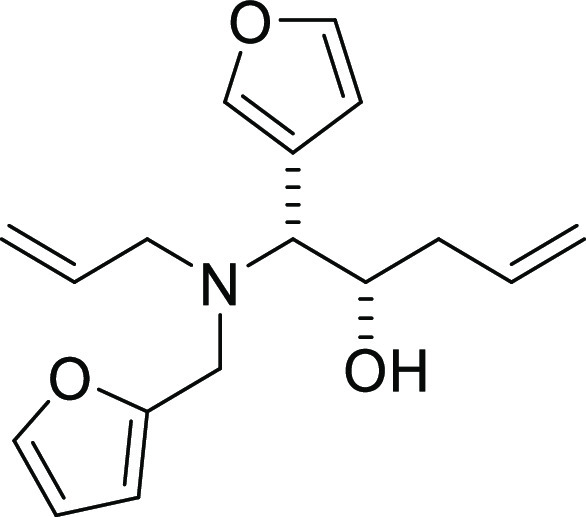



Using the general procedure for the preparation of **4a**–**4i**, **4e** was obtained from *N*-allyl-*N*-(2-furylmethyl) amine (**1c**) (100 mg, 0.73 mmol), 5-allyl-2,2-dimethyl-1,3-dioxolan-4-ol **2** (138.38 mg, 0.87 mmol), and furan-3-boronic acid **3d** (97.879 mg, 0.87 mmol) in HFIP (5 mL) for 2 h. Purified as a colorless oil (168.60 mg, 80% yield). ^1^H NMR (500 MHz, CDCl_3_) δ 7.44 (t, *J* = 1.5 Hz, 1H), 7.38–7.37 (m, 2H), 6.44 (d, *J* = 1.5 Hz, 1H), 6.32 (dd, *J* = 3.1, 1.9 Hz, 1H), 6.16 (d, *J* = 2.9 Hz, 1H), 5.86–5.77 (m, 2H), 5.22–5.15 (m, 2H), 5.11–5.07 (m, 2H), 4.11 (m, 1H), 3.82–3.76 (m, 1H), 3.60 (d, *J* = 6.1 Hz, 1H), 3.44–3.37 (m, 1H), 3.26 (m, 1H), 2.90 (dd, *J* = 14.5, 7.5 Hz, 1H), 2.39–2.34 (m, 1H), 2.16–2.10 (m, 1H). ^13^C NMR (126 MHz, CDCl_3_) δ 152.86, 143.22, 142.08, 142.05, 135.98, 135.14, 119.54, 117.81, 117.69, 111.21, 110.27, 108.64, 70.31, 59.79, 53.81, 46.99, 38.67. HRMS-ESI (*m*/*z*): calculated for [M + H]^+^ C_17_H_22_NO_3_, 288.1594; found, 288.1594.

#### (1*S*,2*R*)-1-(Allyl(furan-2-ylmethyl)amino)-1-(thiophen-2-yl)pent-4-en-2-ol (**4f**)

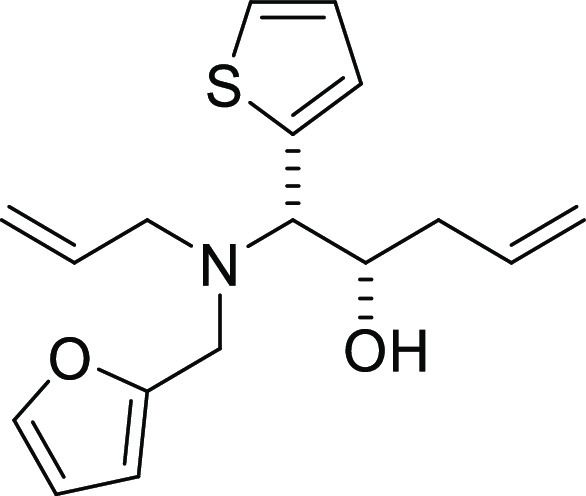



Using the general procedure for the preparation of **4a**–**4i**, **4f** was obtained from *N*-allyl-*N*-(2-furylmethyl) amine (**1c**) (100 mg, 0.73 mmol), 5-allyl-2,2-dimethyl-1,3-dioxolan-4-ol **2** (138.38 mg, 0.87 mmol), and 2-thienylboronic acid **3a** (111.934 mg, 0.87 mmol) in HFIP (5 mL) for 3 h. Purified as a colorless oil (154.8 mg, 70% yield). ^1^H NMR (600 MHz,CDCl_3_) δ 7.39 (m, 1H), 7.31 (d, *J* = 4.8 Hz, 1H), 7.04 (dd, *J* = 5.4, 3.6 Hz, 1H), 6.98–6.97 (m, 1H), 6.33–6.32 (m, 1H), 6.20 (m, 1H), 5.86–5.80 (m, 2H), 5.24 (dd, *J* = 17.2, 1.2 Hz, 1H), 5.18–5.16 (m, 1H), 5.11–5.08 (m, 2H), 4.20 (m, 1H), 3.96 (m, 1H), 3.82 (d, J = 14.5 Hz, 1H), 3.45 (d, *J* = 14.5 Hz, 1H), 3.28 (m, 1H), 2.93–2.89 (m, 1H), 2.45–2.43 (m, 1H), 2.19 (bs, 1H), 2.17–2.13 (m, 1H). ^13^C NMR (151 MHz, CDCl_3_) δ 152.81, 142.17, 138.67, 136.01, 135.00, 127.94, 126.49, 125.60, 117.91, 117.84, 110.29, 108.81, 70.95, 63.81, 53.82, 46.95, 38.66. HRMS-ESI (*m*/*z*): calculated for [M + H]^+^ C_17_H_22_NO_2_S, 304. 1366; found, 304.1366.

#### (1*S*,2*R*)-1-(Allyl(thiophen-2-ylmethyl)amino)-1-(thiophen-2-yl)pent-4-en-2-ol (**4g**)

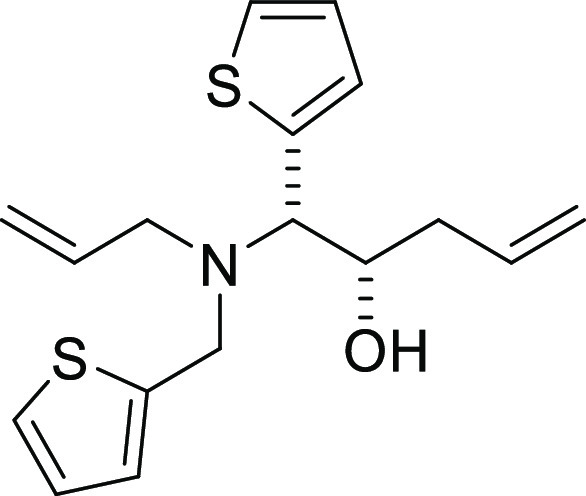



To a solution of **4b** (40 mg, 0.14 mmol) in DCM (2.5 mL) were added triethylamine (43.46 mg, 0.43 mmol) and allyl bromide (86.59 mg, 0.72 mmol). The resultant mixture was allowed to stir for 24 h. The pure product was isolated by flash chromatography using 1% ethyl acetate in petroleum ether. Purified as a yellow oil (21 mg, 45% yield). ^1^H NMR (700 MHz, CDCl_3_) δ 7.32 (d, *J* = 4.9, 1H), δ 7.23 (d, *J* = 4.9, 1H), δ 7.07 (t, *J* = 3.5, 1H), 6.97–6.90 (m, 3H), 5.92–5.82 (m, 2H), 5.26–5.18 (m, 2H), 5.16–5.11 (m, 2H), 4.16 (t, *J* = 8.4, 1H), 4.00 (d, *J* = 7.7, 1H), 3.95 (d, *J* = 14, 1H), 3.55 (d, *J* = 14, 1H), 3.34–3.32 (m, 1H), 2.88 (m, 1H), 2.67–2.64 (m, 1H), 2.24–2.20 (m, 1H), 1.93 (bs, 1H). ^13^C NMR (176 MHz, CDCl_3_) δ 143.83, 138.25, 136.04, 135.16, 127.90, 126.69, 126.61, 125.75, 125.50, 125.03, 118.06, 117.97, 71.59, 63.23, 53.46, 49.62, 38.90. HRMS-ESI (*m*/*z*): calculated for [M + H]^+^ C_17_H_22_NOS_2_, 320.1137; found, 320.1137.

#### (1*R*,2*R*)-1-(Allyl(furan-2-ylmethyl)amino)-1-(*p*-tolyl)pent-4-en-2-ol (**4h**)

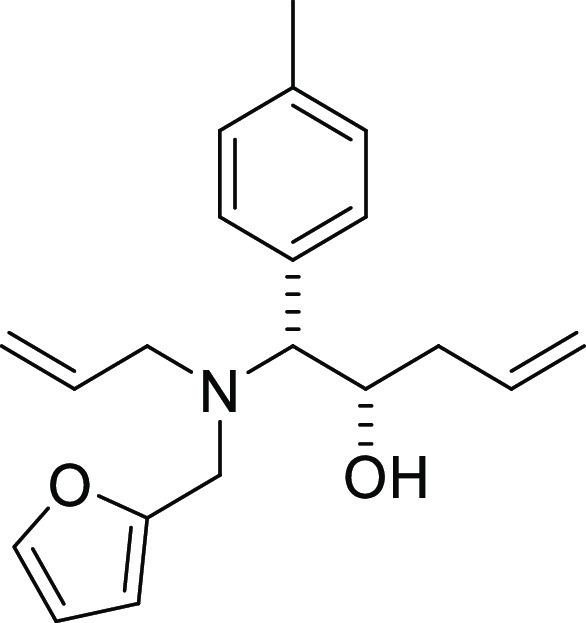



Using the general procedure for the preparation of **4a**–**4i**, **4h** was obtained from *N*-allyl-*N*-(2-furylmethyl) amine (**1c**) (73.33 mg, 0.53 mmol), 5-allyl-2,2-dimethyl-1,3-dioxolan-4-ol **2** (101.33 mg, 0.64 mmol), tolylboronic acid **3e** (87.08 mg, 0.64 mmol) in HFIP (3 mL). Purified as a colorless oil (103.3 mg, 62% yield). ^1^H NMR (400 MHz, CDCl_3_) δ 7.39–7.38 (m, 1H), 7.20 (d, *J* = 8 Hz, 2H), 7.17 (d, *J* = 8 Hz, 2H), 6.33 (dd, *J* = 3.2, 1.6 Hz, 1H), 6.16 (d, *J* = 3.2, 1H), 5.91–5.78 (m, 2H), 5.23–5.15 (m, 2H), 5.09–5.06 (m, 2H), 4.31–4.26 (m, 1H), 3.81 (d, *J* = 15.0 Hz, 1H), 3.58 (d, *J* = 6.4 Hz, 1H), 3.47 (d, *J* = 15.0 Hz, 1H), 3.24 (dd, *J* = 14.5, 5.0 Hz, 1H), 2.94–2.89 (m, 1H), 2.36 (S, 3H) 2.34–2.31 (m, 1H) 2.04–1.97 (m, 1H). ^13^C NMR (101 MHz, CDCl3) δ 152.95, 142.02, 137.44, 135.98, 135.52, 133.23, 129.82, 129.07, 117.77, 117.42, 110.21, 108.69, 69.73, 68.07, 53.40, 46.51, 38.89, 21.26. HRMS-ESI (*m*/*z*): calculated for [M + H]^+^ C_20_H_26_NO_2_, 312.1958; found, 312.1957.

#### (1*R*,2*R*)-1-(Allyl(furan-2-ylmethyl)amino)-1-phenylpent-4-en-2-ol (**4i**)

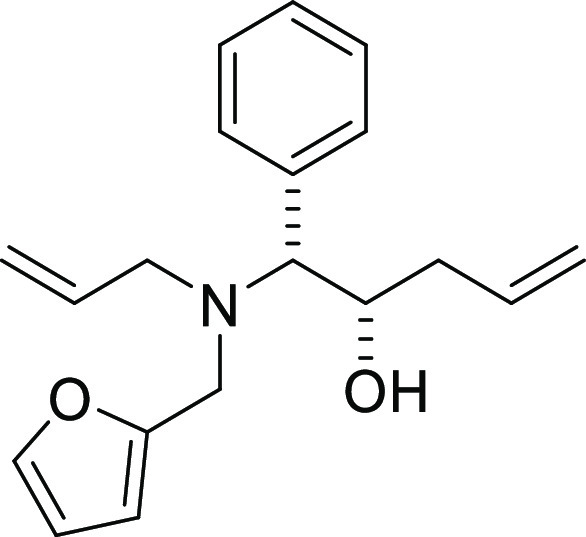



Using the general procedure for the preparation of **4a**–**4i**, **4i** was obtained from *N*-allyl-*N*-(2-furylmethyl) amine (**1c**) (60.00 mg, 0.44 mmol), 5-allyl-2,2-dimethyl-1,3-dioxolan-4-ol **2** (83.63 mg, 0.53 mmol), and phenylboronic acid **3f** (64.46 mg, 0.53 mmol) in HFIP (4 mL). Purified as a white powder (56 mg, 43% yield). ^1^H NMR (600 MHz, CDCl_3_) δ 7.39 (m, 1H), 7.38–7.30 (m, *J* = 5H), 6.33 (m, 1H), 6.17 (m, 1H), 5.84 (m, 2H), 5.22–5.17 (m, 2H), 5.08–5.05 (m, 2H), 4.32 (m, 1H), 3.82 (d, *J* = 12 Hz, 1H), 3.61 (m, 1H), 3.49 (d, *J* = 12.6 Hz, 1H), 3.26–3.24 (m, 1H), 2.95–2.92 (m, 1H), 2.37 (bs, 1H), 2.31–2.30 (m, 1H), 2.01–1.96 (m, 1H). ^13^C NMR (151 MHz, CDCl_3_) δ 152.88, 142.11, 136.49, 135.89, 135.39, 129.98, 128.37, 127.87, 117.87, 117.55, 110.27, 108.80, 69.62, 68.46, 53.44, 46.48, 38.89. HRMS-ESI (*m*/*z*): calculated for [M + H]^+^ C_19_H_24_NO_2_; 298.1802 found, 298.1802.

### General Procedure for the Synthesis of IMDA Products **5a**–**5c**

Petasis products **4g**, **4h**, and **4i** were dissolved in toluene (0.4–0.5 M) and allowed the reaction mixture to stir under reflux conditions, 110 °C. The reaction was monitored by LC-MS and TLC. The solvent then evaporated under reduced pressure. The resultant residue was purified by column chromatography on silica gel (2–5% methanol in DCM).

#### (1*S*,2*R*)-1-((3a*R*,6*R*,7a*R*)-1,6,7,7a-Tetrahydro-3a,6-epoxyisoindol-2(3*H*)-yl)-1-(thiophen-2-yl)pent-4-en-2-ol (**5a**)

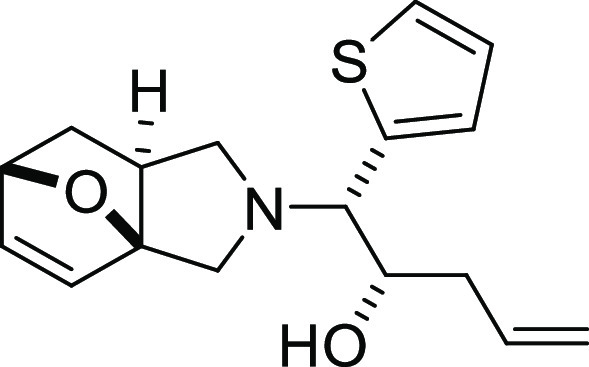



Using the general procedure for the preparation of **5a**–**5c**, **5a** was obtained from **4g** (138 mg, 0.45 mmol) in toluene (3 mL). Purified as a colorless crystal, formed as 1:1 rotamers (81.80 mg, 59% yield). 1:1 rotamers: ^1^H NMR (400 MHz, CDCl_3_) δ 7.29–7.28 (m, 1H), 6.97–6.96 (m, 1H), 6.37 (dd, *J* = 41.2, 5.6, 1H), 6.29–6.26 (m, 1H), 5.86–5.76 (m, 1H), 5.07–5.02 (m, 2H), 4.98–4.97 (m, 1H), 4.12–4.06 (m, 1H), 3.81 (d, *J* = 2.8, 0.50 H, rotamer 1) 3.78–3.76 (m, 1H), 3.48 (t, *J* = 7.6, 0.50 H, rotamer 2), 3.31 (d,, *J* = 12.5 Hz, 0.50 H, rotamer 1), 3.14, 3.02 (two bs, total 1 H, 2 rotamers), 2.88–2.81 (m,1H), 2.75 (d, *J* = 12.5 Hz, 0.50 rotamer 2), 2.28–2.24 (m, 0.50 H, rotamer 1), 2.16–2.11 (m, 0.50 H, rotamer 2), 2.09–1.92 (m, 3H), 1.74–1.71 (m, 0.50 H, rotamer 1) 1.64–1–59 (m, 0.50 H, rotamer 2), 1.37 (dd, *J* = 11.6, 7.6 Hz, 0.50 H, rotamer 1), 1.28 (dd, *J* = 11.2, 7.6 Hz, 0.50 H, rotamer 2). ^13^C NMR (176 MHz, CDCl_3_) δ 140.19 (rotamer 1), 139.92 (rotamer 2), 136.26 (rotamer 1), 136.11 (rotamer 2), 135.91 (rotamer 1), 135.85 (rotamer 2), 134.76 (rotamer 1), 134.68 (rotamer 2), 127.58 (rotamer 1), 127.42 (rotamer 2), 126.11 (rotamer 1), 125.91 (rotamer 2), 125.83 (rotamer 1), 125.71 (rotamer 2), 117.62 (rotamer 1), 117.59 (rotamer 2), 95.86 (rotamer 1), 95.27 (rotamer 2), 80.21 (rotamer 1), 80.11 (rotamer 2), 70.55 (rotamer 1), 70.31 (rotamer 2), 69.19 (rotamer 1), 69.11 (rotamer 2), 57.44(rotamer 1), 57.15 (rotamer 2), 54.11 (rotamer 1), 54.03 (rotamer 2), 43.37 (rotamer 1), 42.75 (rotamer 2), 38.03 (rotamer 1), 37.92 (rotamer 2), 29.54 (rotamer 1), 29.13 (rotamer 2). HRMS-ESI (*m*/*z*): calculated for [M + H]^+^ C_17_H_22_NO_2_S, 304. 1366; found, 304.1366.

#### (1*R*,2*R*)-1-((3a*R*,6*R*,7a*R*)-1,6,7,7a-Tetrahydro-3a,6-epoxyisoindol-2(3*H*)-yl)-1-(p-tolyl)pent-4-en-2-ol (**5b**)

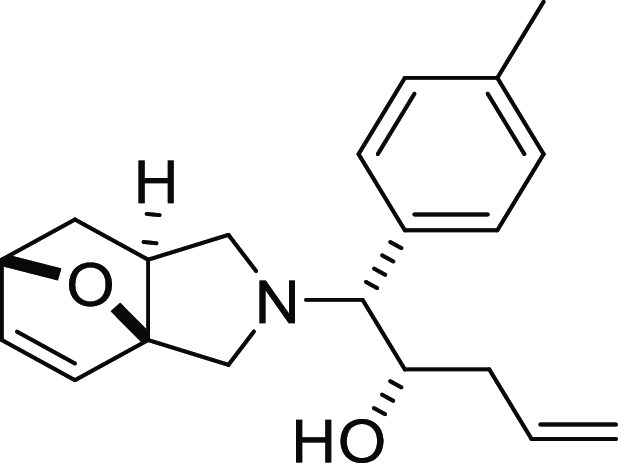



Using the general procedure for the preparation of **5a**–**5c**, **5b** was obtained from **4i** (81 mg, 0.26 mmol) in toluene (1.8 mL). Purified as a colorless crystals, formed as 1:1 rotamers (47.3 mg, 58% yield). 1:1 rotamers: ^1^H NMR (400 MHz, CDCl_3_) δ 7.24–7.11 (m, 4H), 6.44, 6.31 (two d, *J* = 5.6 Hz, total 1H, 2 rotamers), 6.29, 6.26 (two dd, *J* = 5.6, 1.6 Hz, total 1 H, two rotamers), 5.86–5.75 (m, 1H), 5.04–4.96 (m, 3H), 4.12–4.06 (m, 1H), 3.80 (d, *J* = 11.6 Hz, 0.50 H, rotamer 1), 3.56–3.48 (m, 0.50 H, rotamer 1), 3.37 (dd, *J* = 20, 3.2 Hz, 1H), 3.28 (d, *J* = 12.4 Hz, 0.50 H, rotamer 1), 2.91–2.87 (m, 0.50 H, rotamer 2), 2.85 (d, *J* = 11.7 Hz, 0.50 H, rotamer 2), 2.60 (d, *J* = 12.4 Hz, 0.50 H, rotamer 2), 2.34 (m, 3H), 2.30–2.25, 2.06–2.01(two m, total 1H, 2 rotamers), 2.13–2.07, 1.99–1.95 (two m, total 1 H, 2 rotamers), 1.93–1.78 (m, 2H), 1.77–1.71, 1.61–1.55 (two m, total 1H, 2 rotamers), 1.37, 1.26 (two dd, *J* = 11.6, 7.6 Hz, total 1H, two rotamers). ^13^C NMR (101 MHz, CDCl_3_) δ 137.42, 136.12 (rotamer 1), 136.04 (rotamer 2), 135.99 (rotamer 1), 135.87 (rotamer 2), 135.27 (rotamer 1), 135.18 (rotamer 2), 134.80, 129.56 (rotamer 1), 129.49 (rotamer 2), 128.91 (rotamer 1), 128.86 (rotamer 2), 117.24 (rotamer 1), 117.18 (rotamer 2), 95.95 (rotamer 1), 95.34 (rotamer 2), 80.16 (rotamer 1), 80.09 (rotamer 2), 73.77 (rotamer 1), 73.67 (rotamer 2), 71.07 (rotamer 1), 70.74 (rotamer 2), 57.60 (rotamer 1), 57.32 (rotamer 2), 54.54 (rotamer 1), 54.37 (rotamer 2), 43.33 (rotamer 1), 42.89 (rotamer 2), 38.40 (rotamer 1), 38.38 (rotamer 2), 29.55 (rotamer 1), 29.09 (rotamer 2), 21.25. HRMS-ESI (*m*/*z*): calculated for [M + H]^+^ C_20_H_26_NO_2_, 312.1958; found, 312.1957.

#### (1*R*,2*R*)-1-Phenyl-1-((3a*R*,6*R*,7a*R*)-1,6,7,7a-tetrahydro-3a,6-epoxyisoindol-2(3*H*)-yl)pent-4-en-2-ol (**5c**)

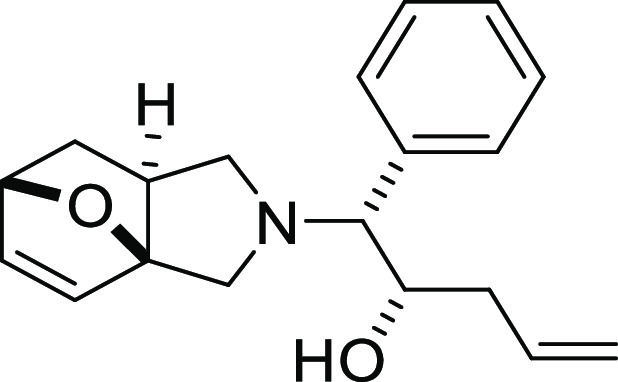



Using the general procedure for the preparation of **5a**–**5c**, **5c** was obtained from **4j** (33 mg, 0.11 mmol) in toluene (1.0 mL). Purified as a white powder, formed as 1:1 rotamers (16.5 mg, 50% yield). 1:1 rotamers: ^1^H NMR (400 MHz, CDCl_3_) δ 7.35–7.31 (m, 5H), 6.46–6.26 (m, total 2H, 2 rotamers), 5.85–5.75 (m, 1H), 5.05–4.99 (m, 3H), 4.16–4.11 (m, 1H), 3.89–3.59 (two singlet, total 1H, 2 rotamers), 3.44 (dd, *J* = 18.8, 1.6, 1H), 3.32, 2.64 (two doublets, *J* = 12.4, total 1H, 2 rotamers), 2.95–2.89 (m, 1H), δ 2.36–2.29, 2.11–2.05 (two multiplets, total 1H, 2 rotamers), 2.17–2.13, 2.03–1.99 (two multiplets, total 1H, 2 rotamers) 1.93–1.80 (m, 2H) 1.78–1.73, 1.61–1.56 (two multiplets, total 1H, 2 rotamers), 1.42–1.28 (m, total 1H, 2 rotamers). ^13^C NMR (101 MHz, CDCl_3_) δ 136.26 (rotamer 1), 136.18 (rotamer 2), 135.8, 135.69 (rotamer 1), 135.68 (rotamer 2), 135.11 (rotamer 1), 135.01(rotamer 2), 129.78 (rotamer 1), 129.72 (rotamer 2), 128.28 (rotamer 1), 128.23 (rotamer 2), 127.99, 117.45 (rotamer 1), 117.37 (rotamer 2), 95.83 (rotamer 1), 95.25 (rotamer 2), 80.26 (rotamer 1), 80.19 (rotamer 2), 74.29 (rotamer 1), 74.23 (rotamer 2), 70.93 (rotamer 1), 70.59 (rotamer 2), 57.78 (rotamer 1), 57.51 (rotamer 2), 54.61(rotamer 1), 54.44 (rotamer 2), 43.30 (rotamer 1), 42.89 (rotamer 2), 38.38 (rotamer 1), 38.37 (rotamer 2), 29.67 (rotamer 1), 29.22 (rotamer 2). HRMS-ESI (*m*/*z*): calculated for [M + H]^+^ C_19_H_24_NO_2_; 298.1802 found, 298.1801.

### General Procedure for the Synthesis of Tertiary Amines with Allyl and Propargyl Appendages (**7a**–**7d**)

Secondary amine (1 equiv), aldehyde (1.2 equiv), and boronic acid (1.2 equiv) were added simultaneously to HFIP (0.5 M) with 3 Å molecular sieves. The mixture was stirred at room temperature and monitored by LC-MS and TLC. The solvent then evaporated under reduced pressure. Purified the resultant residue by column chromatography (5–7% ethyl acetate in petroleum ether).

#### (1*R*,2*R*)-1-(Furan-3-yl)-1-(prop-2-yn-1-yl(thiophen-2-ylmethyl)amino)pent-4-en-2-ol (**7a**)

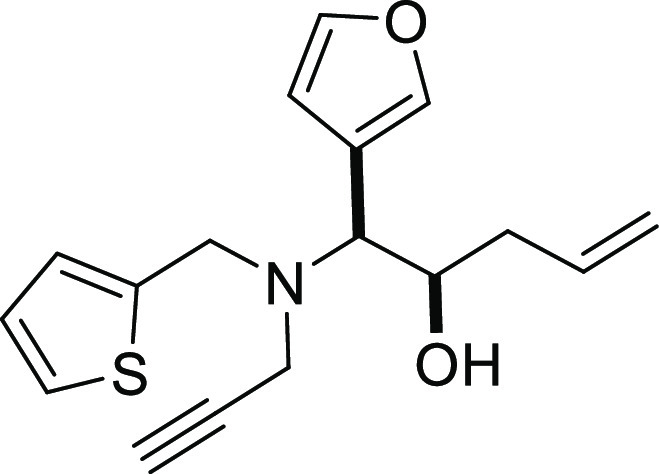



Using the general procedure for the preparation of **7a**–**7d**, **7a** was obtained from *N-*propargyl-thiophenemethylamine **6a** (223.00 mg, 1.47 mmol), 5-allyl-2,2-dimethyl-1,3-dioxolan-4-ol **2** (279.93 mg, 1.77 mmol), and furan-3-boronic acid **3d** (197.99 mg, 1.77 mmol) in HFIP (6 mL). Purified as a colorless oil (365.00 mg, 82% yield). ^1^H NMR (500 MHz, CDCl_3_) δ 7.46 (s, 1H), 7.44–43 (m, 1H), 7.24 (dd, *J* = 3.5 Hz, *J* = 1.5 Hz, 1H) 6.94–6.92 (m, 2H), 6.53 (m, 1H), 5.89–5.80 (m, 1H), 5.10–5.06 (m, 2H), 4.20–4.17 (m, 1H), 3.89 (dd, *J* = 14.5 Hz, *J* = 54 Hz, 2H), 3.76 (d, *J* = 2.5 Hz, 1H), 3.51–3.25 (m, 2H), 2.72 (bs, 1H), 2.25 (t, *J* = 2.5 Hz; 1H), 2.14–2.11 (m, 2H). ^13^C NMR (126 MHz, CDCl_3_) δ 143.35, 142.54, 142.28, 134.88, 126.75, 126.20, 125.30, 120.09, 117.63, 111.28, 78.71, 77.41, 77.16, 76.91, 73.75, 69.33, 60.42, 49.31, 39.32, 38.69. HRMS-ESI (*m*/*z*): calculated for [M + H]^+^ C_17_H_20_NO_2_S, 302.1209; found, 302.1211.

#### (1*S*,2*R*)-1-(Prop-2-yn-1-yl(thiophen-2-ylmethyl)amino)-1-(thiophen-2-yl)pent-4-en-2-ol (**7b**)

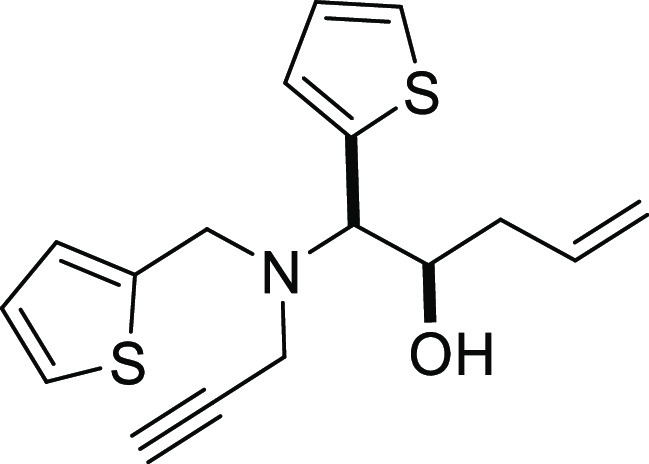



Using the general procedure for the preparation of **7a**–**7d**, **7b** was obtained from *N-*propargyl-thiophenemethylamine **6a** (400.00 mg, 2.64 mmol), 5-allyl-2,2-dimethyl-1,3-dioxolan-4-ol **2** (502.12 mg, 3.17 mmol), and 2-thienylboronic acid **3a** (406.14 mg, 1.77 mmol) in HFIP (5.5 mL). Purified as a pale yellow oil (668.00 mg, 80% yield). ^1^H NMR (600 MHz, CDCl_3_) δ 7.32 (d, *J* = 4.5 Hz, 1H), 7.23 (d, *J* = 4.5 Hz, 1H), 7.07 (d, *J* = 3, Hz, 1H), 7.02 (dd, *J* = 5.1, 3.5 Hz, 1H), 6.95 (m, 1H), 6.92 (dd, *J* = 5.0, 3.4 Hz, 1H), 5.89–5.82 (m, 1H), 5.11–5.08 (m, 2H), 4.27–4.24 (m, 1H), 4.12 (d, *J* = 1.8 Hz, 1H), 3.85 (dd, *J* = 49.2, 13.8 Hz, 1H), 3.55 (d, *J* = 17.4 Hz, 1H), 3.30 (d, *J* = 17.4 Hz, 1H), 2.52 (bs, 1H), 2.27 (s, 1H), 2.19–2.07 (m, 2H). ^13^C NMR (151 MHz, CDCl_3_) δ 142.35, 138.88, 134.70, 128.38, 126.67, 126.31, 126.26, 125.38, 117.84, 78.58, 73.89, 69.52, 64.51, 49.54, 39.14, 38.73. HRMS-ESI (*m*/*z*): calculated for [M + H]^+^ C_17_H_20_NOS_2_, 318.0981; found, 318.0981.

#### (1*R*,2*R*)-1-(2-Bromophenyl)-1-(prop-2-yn-1-yl(thiophen-2-ylmethyl)amino)pent-4-en-2-ol (**7c**)

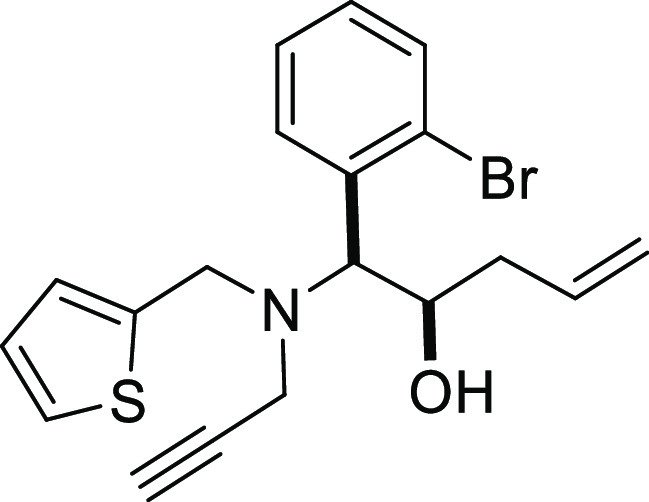



Using the general procedure for the preparation of **7a**–**7d**, **7c** was obtained from *N-*propargyl-thiophenemethylamine **6a** (300.00 mg, 1.98 mmol), 5-allyl-2,2-dimethyl-1,3-dioxolan-4-ol **2** (376.58 mg, 2.38 mmol), and 2-bromophenyl boronic acid **3g** (478.06 mg, 2.38 mmol) in HFIP (4.0 mL). Purified as pale yellow oil. (573.60 mg, 74% yield). ^1^H NMR (700 MHz, CDCl_3_) δ 7.81 (d, *J* = 8.4 Hz, 1H), 7.58 (dd, *J* = 7.7 Hz, *J* = 1.4 Hz, 1H), 7.35 (t, *J* = 7.7 Hz, 1H), 7.21 (d, *J* = 4.9 Hz, 1H), 7.18–7.15 (m, 1H), 6.92–6.90 (m, 2H), 5.87–5.81 (m, 1H), 5.07–5.03 (m, 2H), 4.44 (m, 1H), 4.30 (d, *J* = 8.4, 1H), 3.99 (d, *J* = 14, 1H), 3.70 (d, *J* = 14.7 Hz, 1H), 3.63 (d, *J* = 17.9 Hz, 1H), 3.45 (d, *J* = 17.8 Hz, 1H). ^13^C NMR (176 MHz, CDCl_3_) δ 142.54, 137.11, 135.20, 133.09, 131.36, 129.36, 127.70, 126.67, 126.56, 126.18, 125.24, 117.77, 78.31, 74.25, 69.67, 66.75, 49.36, 38.80, 38.28. HRMS-ESI (*m*/*z*): calculated for [M + H]^+^ C_19_H_21_BrNOS, 390.0522; found, 390.0525.

#### (1*S*,2*R*)-1-(But-2-yn-1-yl(thiophen-2-ylmethyl)amino)-1-(thiophen-2-yl)pent-4-en-2-ol (**7d**)

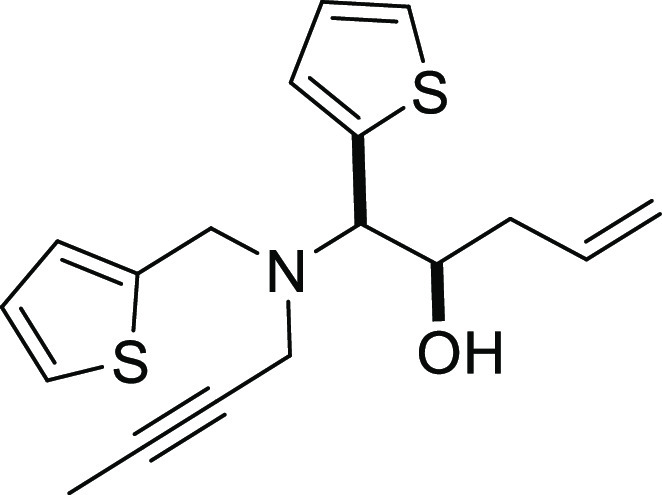



Using the general procedure for the preparation of **7a**–**7d**, **7d** was obtained from *N*-(thiophen-2-ylmethyl)but-2-yn-1-amine **6b** (324.10 mg, 1.96 mmol), 5-allyl-2,2-dimethyl-1,3-dioxolan-4-ol **2** (372.30 mg, 2.35 mmol), and 2-thienylboronic acid **3a** (301.14 mg, 2.35 mmol) in HFIP (4.0 mL). Purified as a colorless oil (591.62 mg, 91% yield). ^1^H NMR (400 MHz, CDCl_3_) δ 7.31 (d, *J* = 4.8 Hz, 1H), 7.21 (dd, *J* = 4.8 Hz, *J* = 1.6 Hz, 1H), 7.05 (d, *J* = 2.8 Hz, 1H), 7.02–7.00 (m, 1H), 6.93–6.91 (m, 2H), 5.92–5.82 (m, 1H), 5.12–5.08 (m, 2H), 4.25–4.24 (m, 1H), 4.12 (d,, *J* = 4.0 Hz, 1H), 3.86 (d, *J* = 14.4 Hz, 1H), 3.76 (d, *J* = 14.0 Hz, 1H), 3.48 (d, *J* = 17.2 Hz, 1H), 3.23 (d, *J* = 17.2 Hz, 1H), 2.56 (bs, 1H), 2.21–2.06 (m, 2H), 1.87 (t, 2.4) ^13^C NMR (101 MHz, CDCl_3_) δ 142.87, 139.18, 134.92, 128.16, 126.60, 126.20, 126.05, 125.15, 117.61, 81.55, 73.69, 69.62, 64.37, 49.58, 39.58, 38.66, 3.73. HRMS-ESI (*m*/*z*): calculated for [M + H]^+^ C_18_H_22_NOS_2_, 332.1137; found, 332.1140.

### General Procedure for Enyne Metathesis of Petasis Products **8**

Petasis products **7** (1 equiv), dissolved in DCM (0.05 M) were added into an oven-dried Schlenk tube followed by Grubbs first generation catalyst (10 mol %, 0.1 equiv). The reaction was heated at 40 °C for 4 h under an argon atmosphere, whereupon the volatiles were removed *in vacuo*. The residue was purified by column chromatography on silica gel (10–20% EA in petroleum ether).

#### (2*S*,3*R*)-2-(Furan-3-yl)-1-(thiophen-2-ylmethyl)-6-vinyl-2,3,4,7-tetrahydro-1*H*-azepin-3-ol (**8a**)

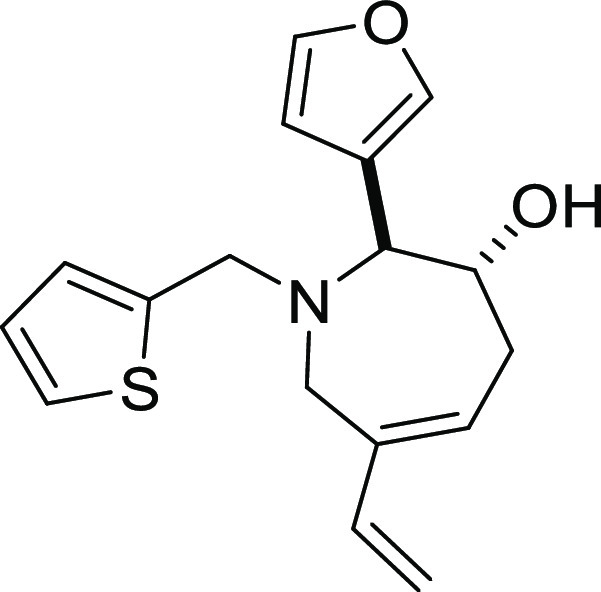



Using the general procedure for the preparation of **8a**–**8d**, **8a** was obtained from **7a** (388.00 mg, 1.29 mmol), using Grubbs first generation catalyst (106.16 mg, 0.129 mmol) in DCM (25 mL, 0.05 M). Purified as a brown oil (104.76 mg, 27% yield). ^1^H NMR (500 MHz, CDCl_3_) δ 7.45 (m, 1H), 7.44 (m, 1H), 7.22 (dd, *J* = 5, 1.5 Hz, 1H), 6.92 (dd, *J* = 5.0, 3.5 Hz, 1H), 6.87–6.86 (m, 1H), 6.46 (m, 1H), 6.30 (dd, *J* = 17.6, 11.0 Hz, 1H), 5.79 (t, *J* = 5.8 Hz, 1H), 4.86–4.79 (m, 2H), 3.99–396 (m, 1H), 3.92–3.90 (m, 3H), 3.55–3.50 (m, 1H), 3.40 (d, *J* = 16.5 Hz, 1H), 2.88–2.77 (m, 1H), 2.58–2.54 (m, 1H), 2.36 (bs, 1H). ^13^C NMR (126 MHz, CDCl_3_) δ 144.28, 143.48, 141.16, 139.79, 139.36, 128.68, 126.48, 125.44, 125.17, 123.06, 110.97, 110.86, 65.11, 54.63, 47.87, 34.34. HRMS-ESI (*m*/*z*): calculated for [M + H]^+^ C_17_H_20_NO_2_S, 302.1209; found, 302.1209.

#### (2*R*,3*R*)-2-(thiophen-2-yl)-1-(thiophen-2-ylmethyl)-6-vinyl-2,3,4,7-tetrahydro-1H-azepin-3-ol (**8b**)

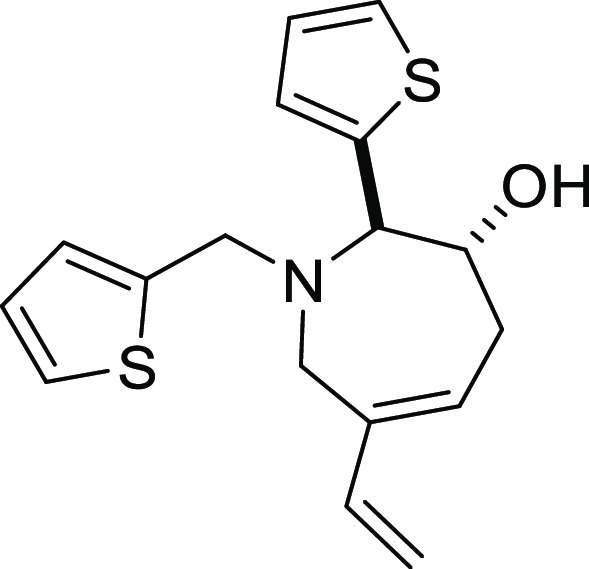



Using the general procedure for the preparation of **8a**–**8d**, **8b** was obtained from **7b** (600.00 mg, 1.89 mmol), using Grubbs first generation catalyst (155.53 mg, 0.189 mmol) in DCM (35 mL, 0.05 M). Purified as a brown oil (196.8 mg, 33% yield). ^1^H NMR (500 MHz, CDCl_3_) δ 7.31 (m, 1H), 7.22 (dd, *J* = 5.0 Hz, *J* = 1.0 Hz, 1H), 7.05–7.04 (m, 2H), 6.92 (dd, *J* = 5.0 Hz, *J* = 3.5 Hz, 1H), 6.89–6.88 (m, 1H), 6.31 (dd, *J* = 17.5 Hz, *J* = 11 Hz, 1H) 5.81 (t, *J* = 5.5 Hz, 1H), 4.87–4.81 (m, 2H), 4.19 (d, *J* = 7.5 Hz, 1H), 4.13–4.10 (m, 1H), 3.97 (d, *J* = 14.0 Hz, 1H), 3.90 (d, *J* = 14.5 Hz, 1H), 3.68 (d, *J* = 17.0 Hz, 1H), 3.42 (d, *J* = 17.0 Hz, 1H), 2.87–2.82 (m, 1H), 2.63 (dd, *J* = 16.5, 7.5 Hz, 1H). ^13^C NMR (126 MHz, CDCl_3_) δ 144.05, 143.52, 139.75, 139.20, 128.5, 126.89, 126.55, 126.42, 125.61, 125.36, 125.23, 111.02, 74.40, 68.88, 54.67, 47.28, 34.50. HRMS-ESI (*m*/*z*): calculated for [M + H]^+^ C_17_H_20_NOS_2_, 318.0981; found, 318.0980.

#### (2*S*,3*R*)-2-(2-bromophenyl)-1-(thiophen-2-ylmethyl)-6-vinyl-2,3,4,7-tetrahydro-1*H*-azepin-3-ol (**8c**)

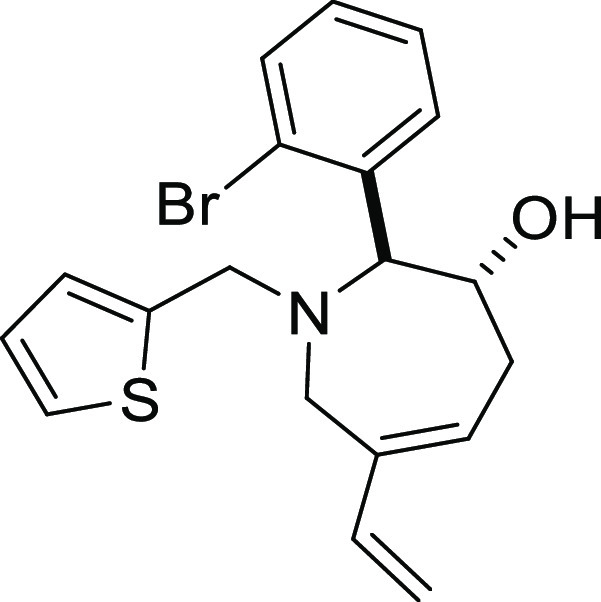



Using the general procedure for the preparation of **8a**–**8d**, **8c** was obtained from **7c** (546.00 mg, 1.40 mmol), using Grubbs first generation catalyst (155.21 mg, 0.140 mmol) in DCM (28 mL, 0.05 M). Purified as a brown oil (142 mg, 26% yield). ^1^H NMR (500 MHz, CDCl_3_) δ 7.77–7.75 (m, 1H), 7.61 (dd, *J* = 8.0, 1 Hz, 1H), 7.35 (t, *J* = 7.0 Hz, 1H), 7.18–7.13 (m, 2H), 6.89 (dd, *J* = 5.0, 3.5 Hz, 1H), 6.81 (d, *J* = 3.0 Hz, 1H), 6.33 (dd, *J* = 17.5, 11.0 Hz, 1H), 5.73 (t, *J* = 5.2 Hz, 1H), 4.86–4.78 (m, 2H), 4.32 (d, *J* = 4.5 Hz, 1H), 4.06 (m, 1H), 3.76–3.69 (m, 3H),), 3.49 (d, *J* = 16.5 Hz, 1H), 2.82–2.78 (m, 1H), 2.54–2.48 (m, 1H), 1.89 (d, *J* = 4.5 Hz, 1H). ^13^C NMR (126 MHz, CDCl_3_) δ 143.16, 140.60, 140.15, 139.11, 133.48, 129.10, 129.01, 127.95, 127.59, 126.49, 125.74, 125.65, 124.91, 111.14, 73.83, 72.22, 53.85, 48.61, 32.30. HRMS-ESI (*m*/*z*): calculated for [M + H]^+^ C_19_H_21_BrNOS, 390.0522; found, 390.0522.

#### (2*R*,3*R*)-6-(Prop-1-en-2-yl)-2-(thiophen-2-yl)-1-(thiophen-2-ylmethyl)-2,3,4,7-tetrahydro-1*H*-azepin-3-ol (**8d**)

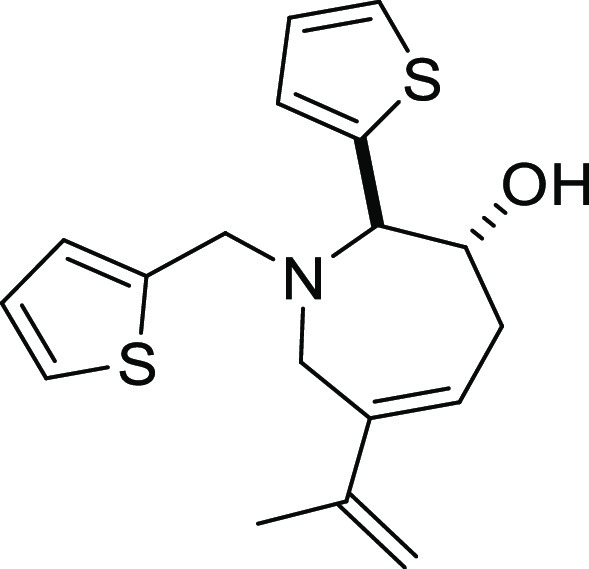



Using the general procedure for the preparation of **8a**–**8d**, **8d** was obtained from **7d** (540.00 mg, 1.63 mmol), using Grubbs first generation catalyst (134.1425 mg, 0.163 mmol) in DCM (32 mL, 0.05 M). Purified as a brown oil (143 mg, 27% yield). ^1^H NMR (500 MHz, CDCl_3_) δ 7.31–7.30 (m, 1H), 7.21 (d, *J* = 5.0 Hz, 1H), 7.04–7.02 (m, 2H), 6.92–6.90 (m, 1H), 6.88 (d, *J* = 3.3 Hz, 1H), 5.91 (t, *J* = 6 Hz, 1H), 4.78 (s, 1H), 4.70 (s, 1H), 4.15 (d, *J* = 7.5 Hz, 1H), 4.12–4.07 (m, 1H), 3.96 (d, *J* = 14.0 Hz, 1H), 3.90 (d, *J* = 14.5 Hz, 1H), 3.77 (d, *J* = 16.5 Hz, 1H), 3.45 (d, *J* = 16.5 Hz, 1H), 2.87–2.83 (m, 1H), 2.64 (dd, *J* = 16.0, 7.5 Hz, 1H), 1.88 (s, 3H). ^13^C NMR (126 MHz, CDCl_3_) δ 144.23, 144.05, 143.20, 141.47, 126.83, 126.48, 126.40, 125.53, 125.36, 125.15, 123.80, 111.13, 74.43, 69.18, 54.61, 49.15, 34.46, 21.63. HRMS-ESI (*m*/*z*): calculated for [M + H]^+^ C_18_H_22_NOS_2_, 332.1137; found, 332.1139.

#### ((7*R*,8*R*,9a*S*)-8-Hydroxy-7-(thiophen-2-yl)-6-(thiophen-2-ylmethyl)-5,6,7,8,9,9a-hexahydro-1*H*-pyridazino[4,3-*c*]azepine-1,2(3*H*)-diyl)bis(piperidin-1-ylmethanone) (**9a**)

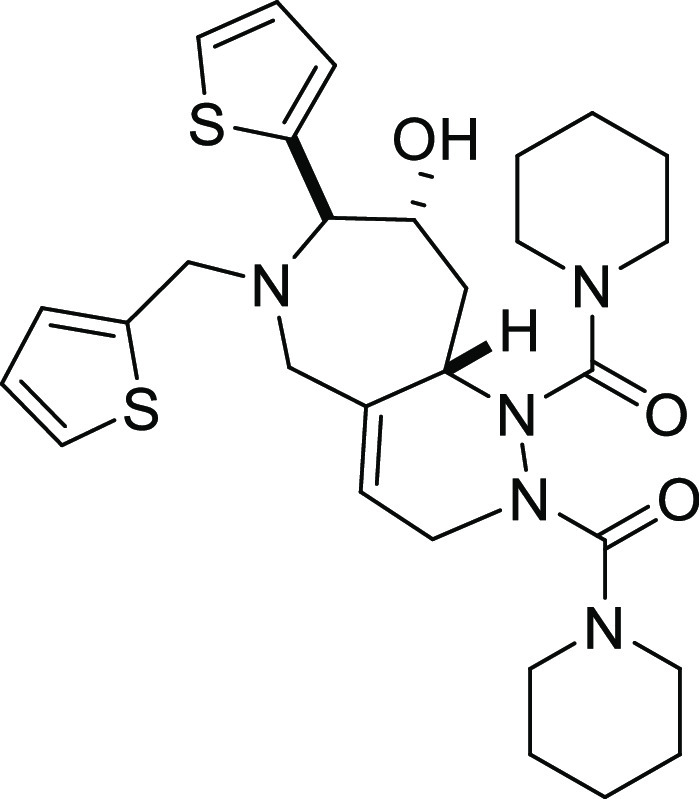



PR/RCM product **8a** (80.3 mg, 0.25 mmol, 1 equiv) was dissolved in toluene (0.5 M) and then 1,1′-(azodicarbonyl)dipiperidine (70.20 mg, 0.28 mmol, 1.1 equiv) was added. The reaction was stirred in reflux overnight. The solvent was evaporated and purified as a colorless crystal in 69% yield (*dr* 9:1) by column chromatography using ethyl acetate in petroleum ether 20–45%. The major diastereomer: ^1^H NMR (500 MHz, CDCl_3_) δ 7.29–7.28 (m, 1H), 7.19 (m, 1H), 7.07 (m, 1H), 7.00 (m, 1H), 6.90–6.86 (m, 2H), 5.58 (m, 1H), 4.50 (t, *J* = 8.5, 1H), 4.20 (m, 2H), 4.11 (m, 1H), 4.04 (m, 1H), 3.91 (d, *J* = 14.0 Hz, 1H), 3.69 (d, *J* = 11.5 Hz, 1H), 3.57 (d, *J* = 15.5 Hz, 1H), 3.43–3.47 (m, 2H), 3.33–3.31 (m, 2H), 3.30–3.28 (m, 4H), 2.71–2.70 (m, 1H), 2.10 (m, 1H), 1.69–1.67 (m, 5H), 1.57–1.53 (m, 8H). ^13^C NMR (126 MHz, CDCl_3_) δ 162.78, 162.47, 146.30, 143.55, 134.30, 127.07, 126.60, 125.90, 125.80, 125.13, 124.98, 119.27, 73.82, 69.80, 58.11, 53.81, 52.96, 47.28, 47.22, 43.41, 36.94, 26.05, 25.96, 24.71. HRMS-ESI (*m*/*z*): calculated for [M + H]^+^ C_29_H_40_N_5_O_3_S_2_, 570.2567; found, 570.2557.

#### ((7*S*,8*R*,9a*S*)-7-(Furan-3-yl)-8-hydroxy-6-(thiophen-2-ylmethyl)-5,6,7,8,9,9a-hexahydro-1H-pyridazino[4,3-*c*]azepine-1,2(3*H*)-diyl)bis(piperidin-1-ylmethanone) (**9b**)

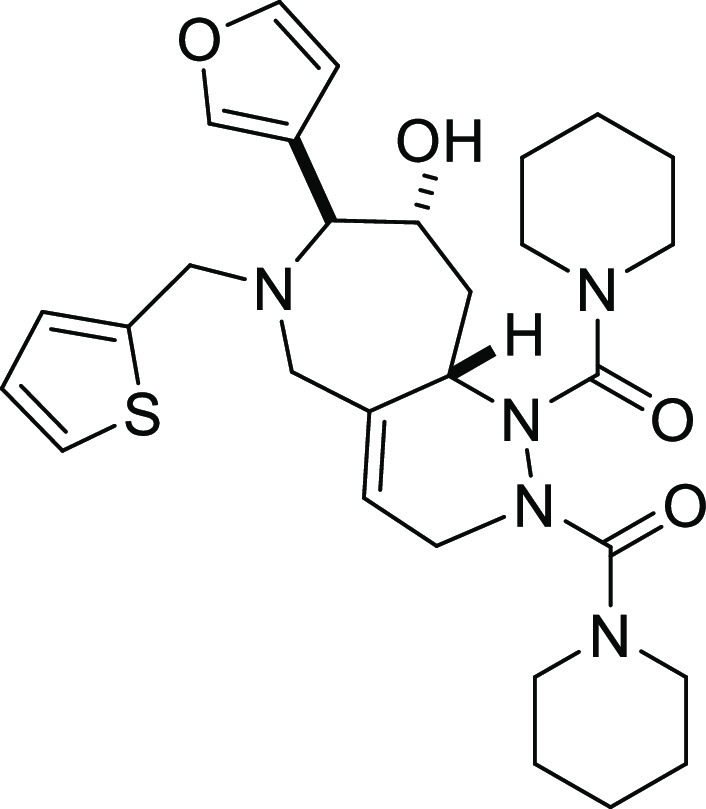



PR/RCM product **8b** (123.3 mg, 0.41 mmol, 1 equiv) was dissolved in toluene (0.5 M) and then 1,1′-(azodicarbonyl)dipiperidine (113.54 mg, 0.45 mmol, 1.1 equiv) was added. The reaction mixture was stirred under the reflux condition overnight. The solvent was evaporated and the product was purified as an off-white powder in 60% yield (*dr* 1:9) by column chromatography using ethyl acetate in petroleum ether 0%:15%:25% then methanol in DCM 0%:2%:4%. The major diastereomer: ^1^H NMR (500 MHz, CDCl_3_) δ 7.49 (m, 1H), 7.44 (m, 1H), 7.17 (d, *J* = 4.5 Hz, 1H), 6.89 (t, *J* = 3.5 Hz, 1H), 6.81 (m, 1H), 6.48 (m, 1H), 5.54 (m, 1H), 4.36 (t, *J* = 7.0 Hz, 1H), 4.12 (m, 2H), 4.00 (m, 1H), 3.92 (d, *J* = 14.5 Hz, 1H), 3.78 (d, *J* = 5.0 Hz, 1H), 3.67 (d, *J* = 14.0 Hz, 1H), 3.53 (d, *J* = 15.5 Hz, 1H), 3.43–3.35 (m, 2H), 3.31 (m, 2H), 3.28–3.25 (m, 4H), 2.59–2.56 (m, 1H), 2.48 (bs, 1H), 2.08 m (1H), 1.66 (m, 4H), 1.55–1.53 (m, 9 H). ^13^C NMR (126 MHz, CDCl_3_) δ 162.50, 144.38, 143.78, 140.76, 126.72, 126.70, 125.33, 125.24, 124.60, 119.18, 109.97, 72.89, 65.38, 57.91, 54.25, 54.21, 47.23, 47.21, 37.85, 25.94, 24.70. HRMS-ESI (*m*/*z*): calculated for [M + H]^+^ C_29_H_40_N_5_O_4_S, 554.2796; found, 554.2788.

#### ((7*R*,8*R*,9a*S*)-8-Hydroxy-7-(thiophen-2-yl)-6-(thiophen-2-ylmethyl)-5,6,7,8,9,9a-hexahydro-1H-pyridazino[4,3-*c*]azepine-1,2(3H)-diyl)bis(morpholinomethanone) (**9c**)

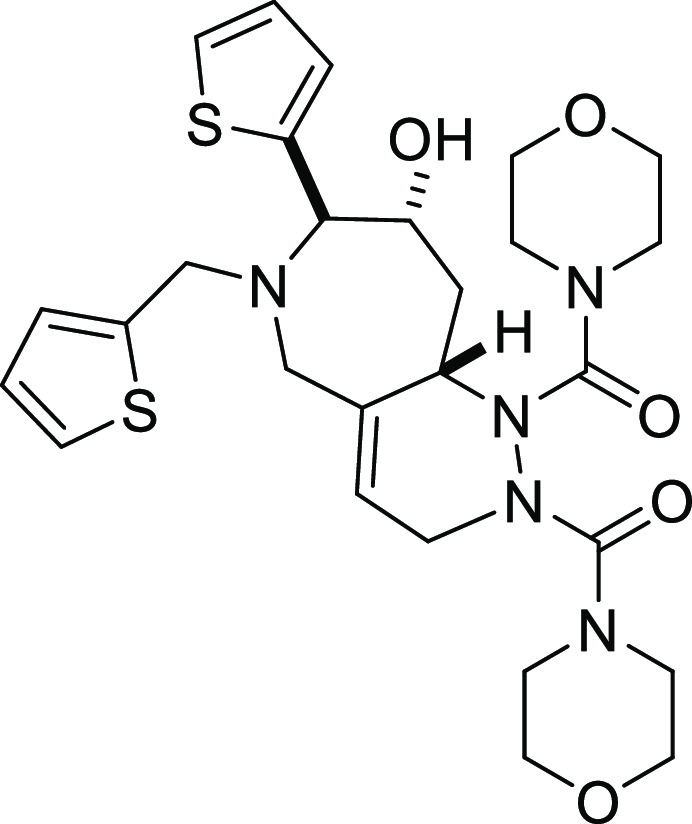



PR/RCM product **8b** (70 mg, 0.22 mmol, 1 equiv) was dissolved in toluene (0.5 M) and then azodicarboxylic dimorpholide (62.15, 0.24 mmol, 1.1 equiv) was added. The reaction mixture was stirred under the reflux condition overnight. The solvent was evaporated and the product was purified as an off-white powder in 73% yield (*dr* 2:1) by column chromatography using 70% ethyl acetate in petroleum ether (isocratic flow). The major diastereomer: ^1^H NMR (700 MHz, CDCl_3_) δ 7.29 (m, 1H), 7.21 (m, 1H), 7.06–6.86 (m, 4H), 5.58 (m, 1H), 4.59 (m, 1H), 4.18 (m, 2H), 4.06 (m, 1H), 3.93 (d, *J* = 13.8 Hz, 1H), 3.85–3.83 (m, 2H), 3.73–3.69 (m, 3H), 3.66–3.69 (m, 5H), 3.59–3.56 (m, 3H), 3.46–3.43 (m, 1H), 3.37–3.32 (m, 6H), 2.65 (m, 1H), 2.11–2.07 (m, 1H). ^13^C NMR (151 MHz, CDCl_3_) δ 162.40, 161.89, 145.66, 143.27, 134.75, 127.21, 126.69, 125.97, 125.12, 125.10, 118.63, 73.61, 69.48, 66.84, 57.78, 53.84, 52.91, 46.65, 43.80, 36.90. HRMS-ESI (*m*/*z*): calculated for [M + H]^+^ C_27_H_36_N_5_O_5_S_2_, 574.2152; found, 574.2142.

#### ((7*S*,8*R*,9a*S*)-7-(Furan-3-yl)-8-hydroxy-6-(thiophen-2-ylmethyl)-5,6,7,8,9,9a-hexahydro-1*H*-pyridazino[4,3-*c*]azepine-1,2(3H)-diyl)bis(morpholinomethanone) (**9d**)

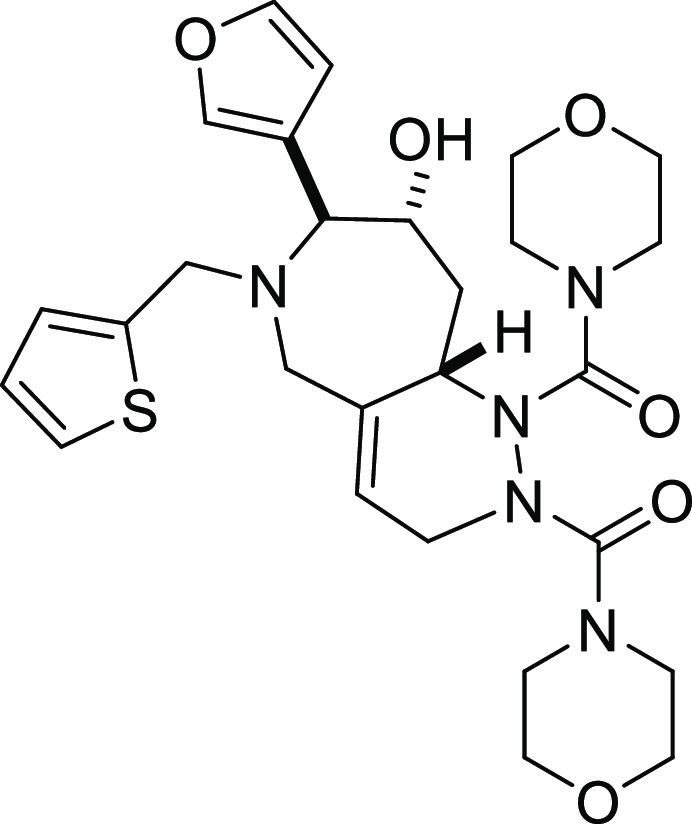



PR/RCM product **8a** (80 mg, 0.27 mmol, 1 equiv) was dissolved in toluene (0.5 M) and then azodicarboxylic dimorpholide (74.82 mg, 0.29 mmol, 1.1 equiv) was added. The reaction was stirred in reflux overnight. The solvent was evaporated and then purified as an off-white powder in 66% yield (*dr* 2:1) by column chromatography using 70% ethyl acetate in petroleum ether (isocratic flow). The major diastereomer: ^1^H NMR (700 MHz, CDCl_3_) δ 7.48 (m, 1H), 7.46 (m, 1H), 7.19 (d, *J* = 4.9 Hz, 1H), 6.89 (t, *J* = 3.5 Hz, 1H), 6.81 (m, 1H), 6.45 (m, 1H), 5.55 (m, 1H), 4.38 (dd, *J* = 8.4, 4.2 Hz, 1H), 4.17 (m, 2H), 4.02 (m, 1H), 3.93 (d, *J* = 14.4 Hz, 1H), 3.80 (d, *J* = 5.8 Hz, 1H), 3.78–3.73 (m, 2H), 3.69–3.64 (m, 7H), 3.54 (d, *J* = 15.4 Hz, 1H), 3.50–3.48 (m, 2H), 3.38–3.29 (m, 7H), 2.52 (d, *J* = 13.4 Hz, 1H), 2.39 (bs, 1H), 2.07 (m, 1H). ^13^C NMR (176 MHz, CDCl_3_) δ 162.32, 161.95, 144.27, 144.00, 140.80, 126.80, 125.42, 124.70, 124.53, 118.96, 111.42, 109.83, 72.36, 66.83, 66.75, 64.93, 57.72, 54.44, 53.28, 46.68, 46.57, 44.00, 38.33. HRMS-ESI (*m*/*z*): calculated for [M + H]^+^ C_27_H_36_N_5_O_6_S, 558.2381; found, 558.2371.

#### Di-*tert*-butyl (7*R*,8*R*,9a*S*)-8-hydroxy-7-(thiophen-2-yl)-6-(thiophen-2-ylmethyl)-5,6,7,8,9,9a-hexahydro-1*H*-pyridazino[4,3-*c*]azepine-1,2(3*H*)-dicarboxylate (**9e**)

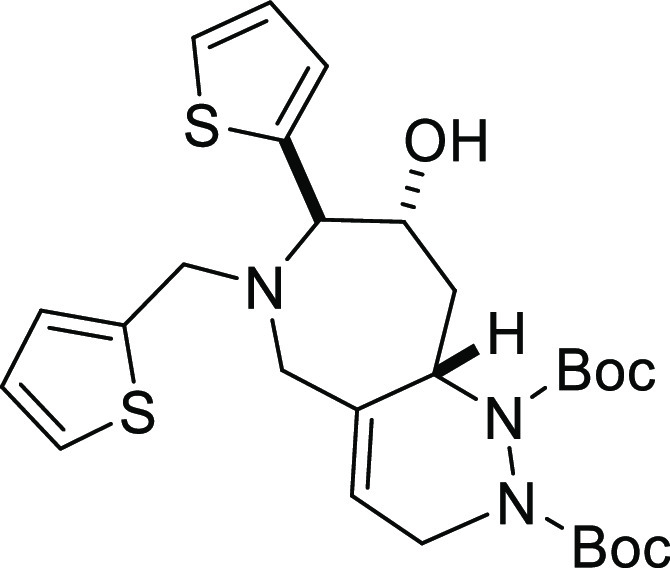



PR/RCM product **8b** (100 mg, 0.31 mmol, 1 equiv) was dissolved in DCM (0.5 M) followed by di-*tert*-butyl azodicarboxylate (79.78 mg, 0.35 mmol, 1.1 equiv) and stirred it in reflux for 2.5 h. The solvent was evaporated and purified as a brown powder in 70.1% yield (6:1 *d.r*) by column chromatography using ethyl acetate in petroleum ether (25% isocratic flow). The major diastereomer: ^1^H NMR (700 MHz, CDCl_3_) δ 7.29 (m, 1H), 7.21 (m, 1H), 7.05–7.00 (m, 2H), 6.90–6.87 (m, 2H), 5.50–5.46 (m, 1H), 4.78 (m, 1H), 4.49–4.29 (m, 1H), 4.09–3.98 (m, 3H), 3.87 (d, *J* = 11.2 Hz, 1H), 3.74–3.72 (m, 1H), 3.49–3.34 (m, 2H), 2.58–2.47 (m, 1H), 2.08 (bs, 1H), 2.01 (m, 1H), 1.50 (s, 9H), 1.47 (s, 9H). ^13^C NMR (176 MHz, CDCl_3_) δ 154.52, 154.27, 146.32, 143.51, 135.26, 126.98, 126.49, 126.15, 125.78, 125.44, 125.34, 120.58, 81.39, 81.10, 72.50, 69.12, 54.22, 53.14, 51.85, 42.32, 39.59, 28.47. HRMS-ESI (*m*/*z*): calculated for [M + H]^+^ C_27_H_38_N_3_O_5_S_2_, 548.2247; found, 548.2236.

#### Di-*tert*-butyl (7*S*,8*R*,9a*S*)-7-(Furan-3-yl)-8-hydroxy-6-(thiophen-2-ylmethyl)-5,6,7,8,9,9a-hexahydro-1*H*-pyridazino[4,3-*c*]azepine-1,2(3*H*)-dicarboxylate (**9f**)

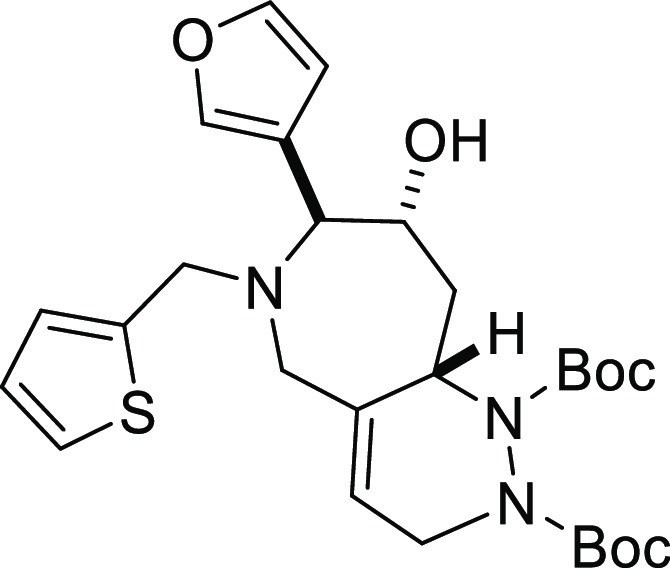



PR/RCM product **8a** (100 mg, 0.33 mmol, 1 equiv) was dissolved in DCM (0.5 M) followed by di-*tert*-butyl azodicarboxylate (84.04 mg, 0.36 mmol, 1.1 equiv) and stirred it in reflux for 3 h. The solvent was evaporated and purified as a brown powder in 65% yield (6:1 *d.r*) by column chromatography using ethyl acetate in petroleum ether (25% isocratic flow). The major diastereomer: ^1^H NMR (700 MHz, CDCl_3_) δ 7.53–7.42 (m, 2 H), 7.24 (m, 1H), 6.94 (m, 2H), 6.55 (m, 1H), 5.49 (m, 1H), 4.82 (m, 1H), 4.43 (d, 17 Hz, 1H), 4.30–3.96 (m, 4H), 3.83–3.71 (m, 1H), 3.61 (d, 18.5 Hz, 1H), 3.47 (m, 1H), 2.47–2.45 (m, 1H), 2.00–1.96 (m, 2H), 1.48 (s, 9H), 1.46 (s, 9H). ^13^C NMR (126 MHz, CDCl_3_) δ 154.40, 154.18, 144.12, 143.90, 143.75, 134.28, 126.67, 125.50, 110.26, 81.42, 81.09, 71.79, 64.92, 53.68, 52.85, 42.21, 37.22, 31.95, 29.72, 28.32. HRMS-ESI (*m*/*z*): calculated for [M + H]^+^ C_27_H_38_N_3_O_6_S, 532.2476; found, 532.2467.

#### Diisopropyl (7*R*,8*R*,9a*S*)-8-Hydroxy-7-(thiophen-2-yl)-6-(thiophen-2-ylmethyl)-5,6,7,8,9,9a-hexahydro-1*H*-pyridazino[4,3-*c*]azepine-1,2(3*H*)-dicarboxylate (**9g**)

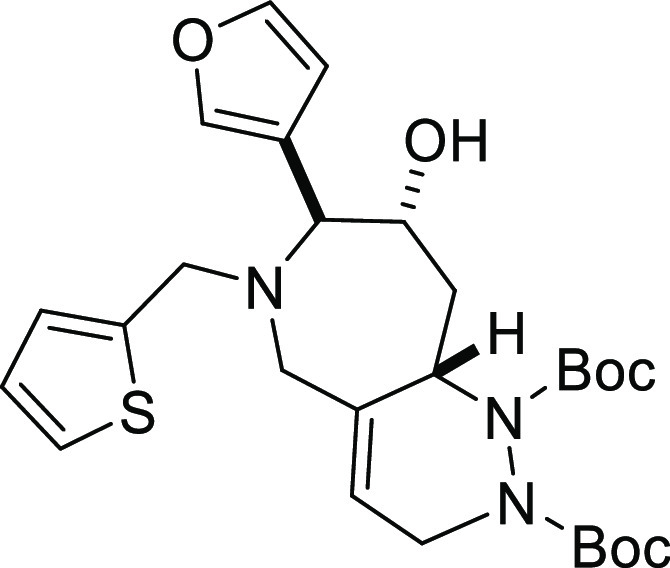



PR/RCM product **8b** (73.0 mg, 0.23 mmol, 1 equiv) was dissolved in DCM (0.5 M) and followed by that diisopropyl azodicarboxylate (51.15 mg, 0.25 mmol, 1.1 equiv) was added. The reaction was stirred in reflux for 4 h. The solvent was evaporated and purified as a brown powder in 80% yield (5:1 *dr*) by column chromatography using 60% ethyl acetate in petroleum ether (isocratic flow). The major diastereomer: ^1^H NMR (500 MHz, CDCl_3_) δ 7.31–7.28 (m, 1H), 7.202 (dd, *J* = 5.0 Hz, 1.0 Hz, 1H), 7.05 (d, *J* = 3.0 Hz, 1H), 6.99 (m, 1H), 6.94–6.86 (m, 2H), 5.49 (m, 1H), 4.99–4.94 (m, 2H), 4.90–4.88 (m, 1H), 4.53–4.33 (m, 1H), 4.21–4.09 (m, 1H), 3.99–3.85 (m, 2H), 3.71–3.64 (m, 1H), 3.49 (d, *J* = 15.0 Hz, 1H), 3.36–3.27 (m, 1H), 2.51–2.43 (m, 1H), 2.16–1.97 (m, 2H), 1.26 (m, 6H), 1.23–1.25 (m, 6H). ^13^C NMR (126 MHz, CDCl3) δ 155.22, 146.19, 143.43, 135.35, 126.95, 126.44, 126.10, 125.75, 125.41, 120.00, 116.70, 72.39, 70.19, 69.99, 69.12, 54.42, 51.75, 42.67, 39.29, 22.23, 22.07. HRMS-ESI (*m*/*z*): calculated for [M + H]^+^ C_25_H_34_N_3_O_5_S_2_, 520.1934; found, 520.1924.

#### (9*R*,10*R*,11a*S*)-10-Hydroxy-2-phenyl-9-(thiophen-2-yl)-8-(thiophen-2-ylmethyl)-7,8,9,10,11,11a-hexahydro-1*H*,5*H*-[1,2,4]triazolo[1′,2′:1,2]pyridazino[4,3-*c*]azepine-1,3(2*H*)-dione (**9h**)

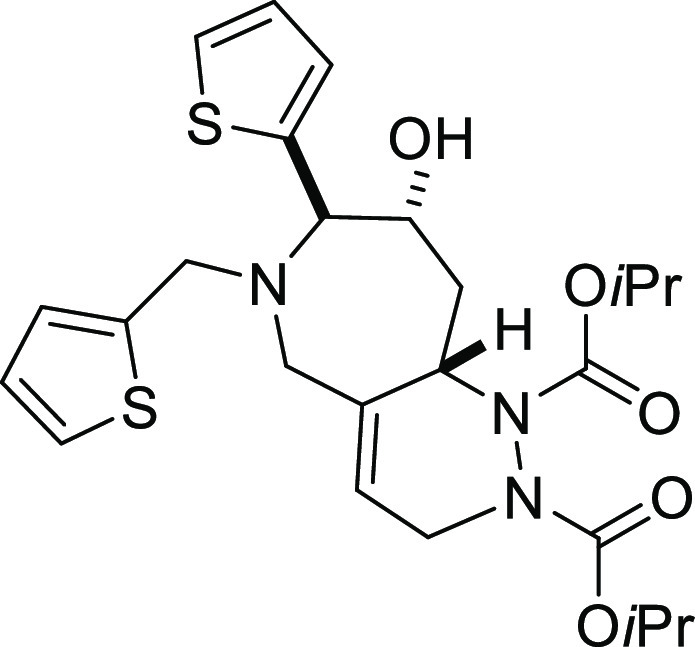



PR/RCM product **8b** (90 mg, 0.28 mmol, 1 equiv) was dissolved in DCM (0.5 M) followed by 4-phenyl-1,2,4-triazoline-3,5-dione (54.80 mg, 0.31 mmol, 1.1 equiv) and stirred it in reflux for 30 min. The solvent was evaporated and purified as a white powder in 55% yield (9:1 *d.r*) using 30% ethyl acetate in petroleum ether. Major diastereomer: ^1^H NMR (500 MHz, CDCl_3_) δ 7.55–7.53 (m, 2H), 7.49–7.46 (m, 2H), 7.39–7.35 (m, 2H), 7.29 (d, *J* = 5.6 Hz, 1H), 7.07 (dd, *J* = 5.0, 3.5 Hz, 1H), 6.97–6.95 (m, 3H), 5.73 (m, 1H), 5.14 (m, 1H), 4.38 (d, *J* = 16.3 Hz, 1H), 4.24–4.23 (m, 2H), 3.95–3.92 (m, 2H), 3.71 (d, *J* = 10.5 Hz, 1H), 3.51–3.48 (m, 2H), 2.68–2.62 (m, 1H), 2.23 (bs, 1H), 2.0–1.98 (1H, m). ^13^C NMR (126 MHz, CDCl_3_) δ 153.43, 151.83, 131.31, 129.26, 128.24, 127.14, 126.81, 125.97, 125.84, 125.45, 114.73, 72.57, 67.42, 54.41, 52.71, 50.96, 43.65, 37.33. HRMS-ESI (*m*/*z*): calculated for [M + H]^+^ C_25_H_25_N_4_O_3_S_2_, 493.1363; found, 493.1352.

#### ((7*R*,8*R*,9a*S*)-8-Hydroxy-4-methyl-7-(thiophen-2-yl)-6-(thiophen-2-ylmethyl)-5,6,7,8,9,9a-hexahydro-1*H*-pyridazino[4,3-*c*]azepine-1,2(3*H*)-diyl)bis(morpholinomethanone) (**9i**)

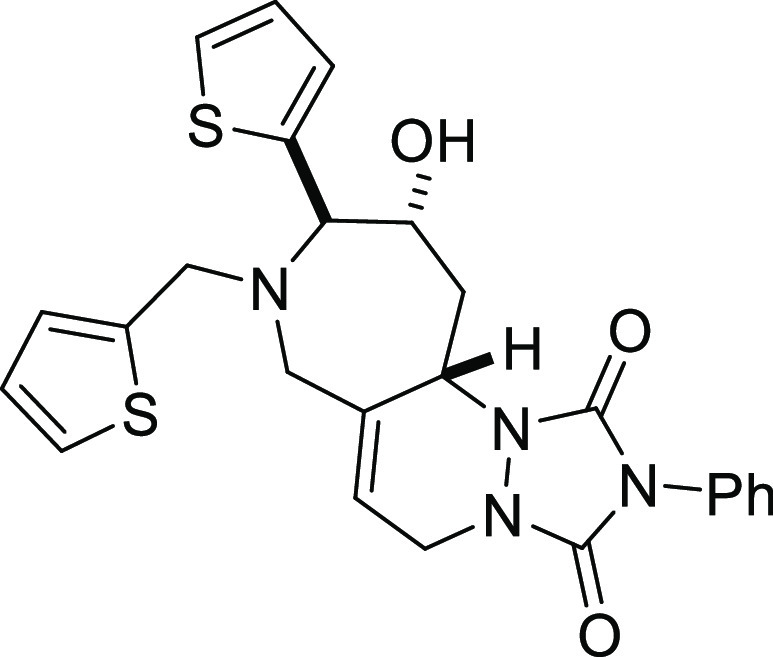



PR/RCM product **8d** (123 mg, 0.37 mmol, 1 equiv) was dissolved in toluene (0.5 M) and then azodicarboxylic dimorpholide (104.59 mg, 0.41 mmol, 1.1 equiv) was added. The reaction was stirred in reflux overnight. The solvent was evaporated and purified as a yellow powder in 72% yield (4:1 *dr*) by column chromatography using 70% ethyl acetate in petroleum ether (isocratic flow). NMR of 5:1 diastereomeric mixture, reporting the major diastereomer. ^1^H NMR (500 MHz, CDCl_3_) δ 7.27–7.26 (m, 1H), δ 7.21 (d, *J* = 4.9 Hz, 1H), 7.06 (d, *J* = 4.9 Hz, 1H), 7.02–6.99 (m, 1H), 6.91 (dd, *J* = 5.0, 3.0 Hz, 1H), 6.87 (m, 1H), 4.92 (t, *J* = 8.2 Hz, 1H), 4.19 (m, 1H), 4.12 (d, *J* = 16.9 Hz, 1H), 3.99–3.91 (m, 5H), 3.78–3.74 (m, 2H), 3.66–3.62 (m, 6H), 3.57 (m, 1H), 3.54–3.53 (m, 2H), 3.38–3.35 (m, 6H), 2.69 (m, 1H), 2.50 (bs, 1H), 2.01–1.94 (m, 1H), 1.55 (s, 3H). ^13^C NMR (126 MHz, CDCl_3_) δ 162.46, 161.76, 146.19, 143.22, 128.04, 127.21, 126.75, 125.85, 125.64, 124.96, 124.71, 123.83, 74.77, 70.26, 66.86, 56.61, 54.79, 51.65, 47.28, 46.55, 34.88, 15.61. HRMS-ESI (*m*/*z*): calculated for [M + H]^+^ C_28_H_38_N_5_O_5_S_2_, 588.2309; found, 588.2297.

#### ((7*S*,8*R*,9a*S*)-7-(2-Bromophenyl)-8-hydroxy-6-(thiophen-2-ylmethyl)-5,6,7,8,9,9a-hexahydro-1*H*-pyridazino[4,3-*c*]azepine-1,2(3*H*)-diyl)bis(morpholinomethanone) (**9j**)

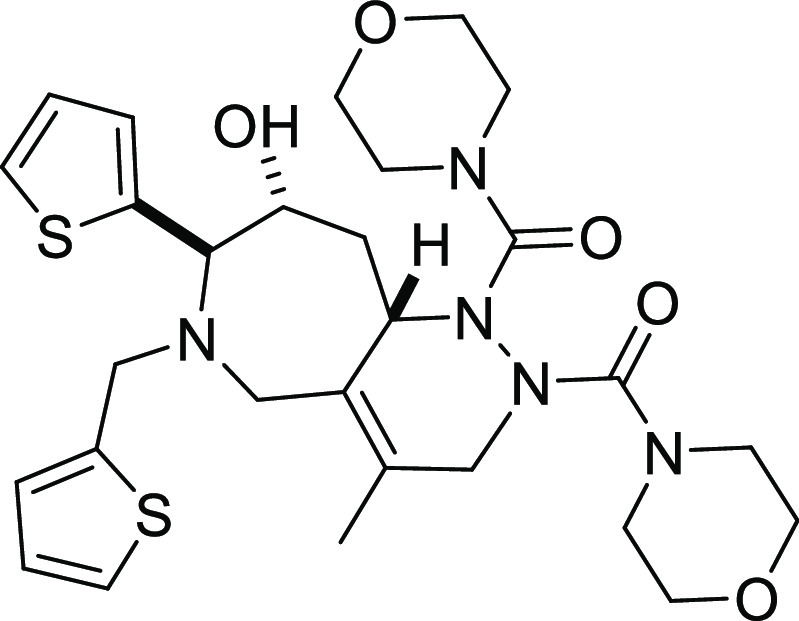



PR/RCM product **8c** (122 mg, 0.31 mmol, 1 equiv) was dissolved in toluene (0.5 M) and then azodicarboxylic dimorpholide (88.10 mg, 0.34 mmol, 1.1 equiv) was added. The reaction was stirred in reflux overnight. The solvent was evaporated and purified as a colorless crystals in 67% yield (*dr* 6:1) by column chromatography using 70% ethyl acetate in petroleum ether (isocratic flow). The major diastereomer: ^1^H NMR (500 MHz, CDCl_3_) δ 7.79 (d, *J* = 7.5 Hz, 1H), 7.62 (dd, *J* = 8.0 Hz, 1 Hz, 1H), 7.39–7.37 (m, 1H), 7.19–7.17 (m, 2H), 6.90–6.88 (m, 1H), 6.80–6.79 (m, 1H), 5.60 (m, 1H), 4.72 (t, *J* = 7.5 Hz, 1H), 4.27 (d, *J* = 4.5 Hz, 1H), 4.21 (m, 2H), 4.10–3.09 (m, 1H), 3.90- 3.86 (m, 2H), 3.77–3.73 (m, 2H), 3.69–3.70 (m, 1H), 3.66–3.64 (m, 5H), 3.61 (m, 1H), 3.59–3.53 (m, 3H), 3.38–3.38 (m, 6H), 2.51–2.46 (m, 1H), 2.06–2.0 (m, 2H). ^13^C NMR (126 MHz, CDCl_3_) δ 162.59, 161.96, 143.55, 139.86, 135.65, 133.87, 129.41, 128.63, 127.80, 126.92, 125.78, 125.62, 124.71, 117.40, 71.93, 71.40, 66.84, 57.30, 53.68, 53.09, 46.65, 43.73, 36.85. HRMS-ESI (*m*/*z*): calculated for [M + H]^+^ C_29_H_37_BrN_5_O_5_S, 646.1693; found, 646.1689.

#### Bis(7-(λ^5^-chloraneyl)hepta-2,4,6-triyn-1-yl) (7*R*,8*R*,9a*S*)-8-hydroxy-7-(thiophen-2-yl)-6-(thiophen-2-ylmethyl)-5,6,7,8,9,9a-hexahydro-1*H*-pyridazino[4,3-*c*]azepine-1,2(3*H*)-dicarboxylate (**9k**)

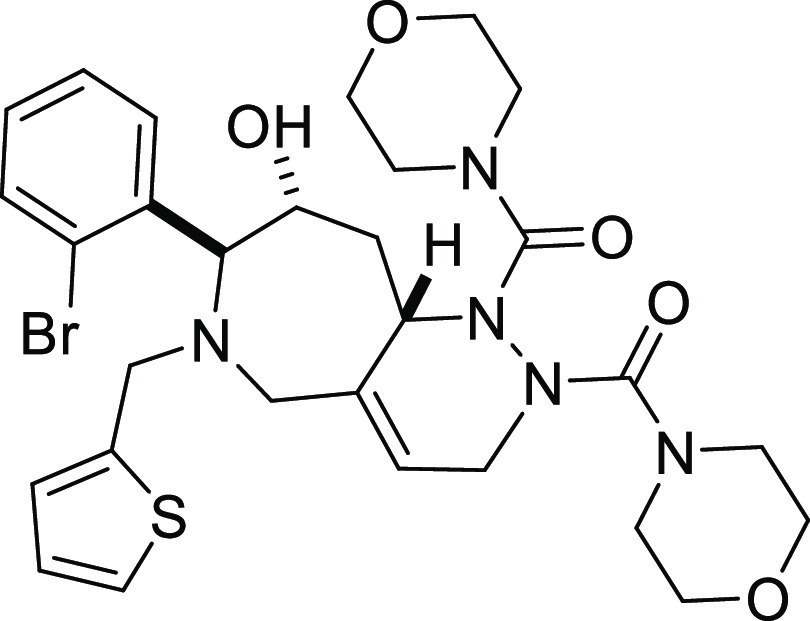



PR/RCM product **8b** (59 mg, 0.19 mmol, 1 equiv) was dissolved in DCM (0.5M) and then di(4-chlorobenzyl) azodicarboxylate (75.06 mg, 0.20 mmol, 1.1 equiv) was added. The reaction was stirred in reflux for 0.5 h. The solvent was vaporated and purified as a brown oily liquid in 59% yield (4:1 *d.r*) by column chromatography using 30% ethyl acetate in petroleum ether (isocratic flow). The major diastereomer: ^1^H NMR (700 MHz, CDCl_3_) δ 7.32–7.10 (m, 10H), 7.14 (m, 1H), 7.03–6.93 (m, 3H), 5.50 (m, 1H), 5.20–5.04 (m, 4H), 4.92 (m, 1H), 4.54–4.41 (m, 1H), 4.12–3.80 (m, 4H), 3.71–3.43 (m, 2H), 2.49–2.64 (m, 1H), 1.90–1.68 (m, 2H). ^13^C NMR (176 MHz, CDCl_3_) δ 155.14, 134.52, 134.38, 134.27, 129.64, 129.56, 129.33, 128.94, 128.89, 128.84, 127.20, 126.73, 126.53, 125.81, 69.07, 67.51, 67.30, 54.64, 53.12, 51.79, 43.28, 29.84. HRMS-ESI (*m*/*z*): calculated for [M + H]^+^ C_33_H_32_Cl_2_N_3_O_5_S_2_, 684.1155; found, 684.1148.

#### Bis(2-methoxyethyl) (7*R*,8*R*,9a*S*)-8-hydroxy-7-(thiophen-2-yl)-6-(thiophen-2-ylmethyl)-5,6,7,8,9,9a-hexahydro-1*H*-pyridazino[4,3-*c*]azepine-1,2(3*H*)-dicarboxylate (**9l**)

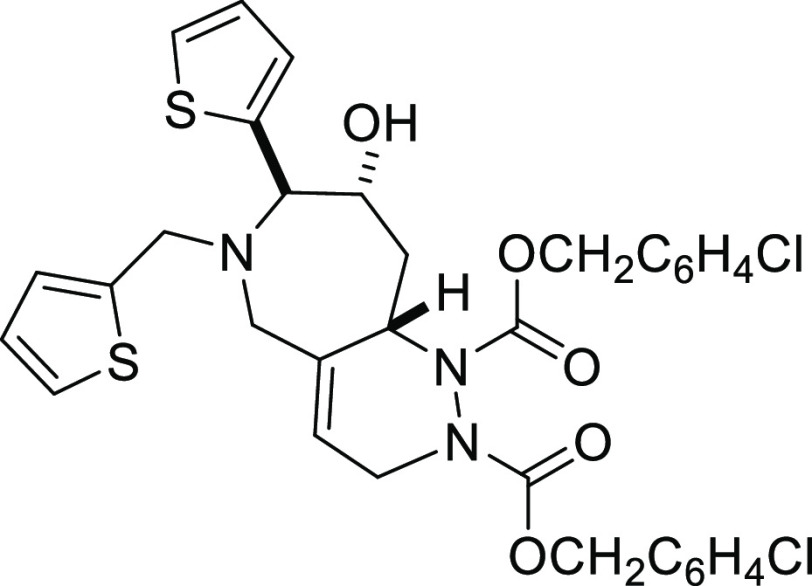



PR/RCM product **8b** (75.5 mg, 0.24 mmol, 1 equiv) was dissolved in DCM (0.5 M) and followed by that di-2-methoxyethyl azodicarboxylate (61.27 mg, 0.26 mmol, 1.1 equiv) was added. Stirred the reaction in reflux for 0.5 h. Evaporated the solvent, purified as a brown oil in 50.3% yield (*dr* 4:1) by column chromatography using 60% ethyl acetate in petroleum ether (Isocratic flow). ^1^H NMR (600 MHz, CDCl_3_) δ 7.28–7.27 (m, 1H), 7.21 (d, *J* = 4.8 Hz, 1H), 7.04 (m, 1H), 7.00–6.98 (m, 1H), 6.90–6.89 (m, 1H), 6.87–6.84 (m, 1H), 5.48–5.44 (m, 1H), 4.90–4.86 (m, 1H), 4.53 (d, *J* = 16.8 Hz, 1H), 4.44–4.39 (m, 1H), 4.29–4.28 (m, 4H), 4.22–4.18 (m, 1H), 4.11 (m, 1H), 4.06–4.01 (m, 1H), 3.90–3.88 (m, 1H), 3.64 (m, 1H), 3.60–3.58 (m, 4H), 3.49–3.46 (m, 1H), 3.40 (m, 1H), 3.37–3.35 (m, 6H), 2.59–2.52 (1H, m), 2.06–2.01 (m, 1H). ^13^C NMR (151 MHz, CDCl_3_) δ 156.56, 155.43, 146.29, 143.55, 134.97, 126.96, 126.46, 125.95, 125.73, 125.33, 125.21, 119.81, 77.37, 77.16, 76.95, 72.45, 70.61, 69.00, 65.61, 65.18, 59.02, 54.70, 53.19, 51.61, 38.98. HRMS-ESI (*m*/*z*): calculated for [M + H]^+^ C_25_H_34_N_3_O_7_S_2_, 552.1828; found, 552.1833.

#### Dibenzyl (7*R*,8*R*,9a*S*)-8-Hydroxy-7-(thiophen-2-yl)-6-(thiophen-2-ylmethyl)-5,6,7,8,9,9a-hexahydro-1*H*-pyridazino[4,3-*c*]azepine-1,2(3*H*)-dicarboxylate (**9m**)

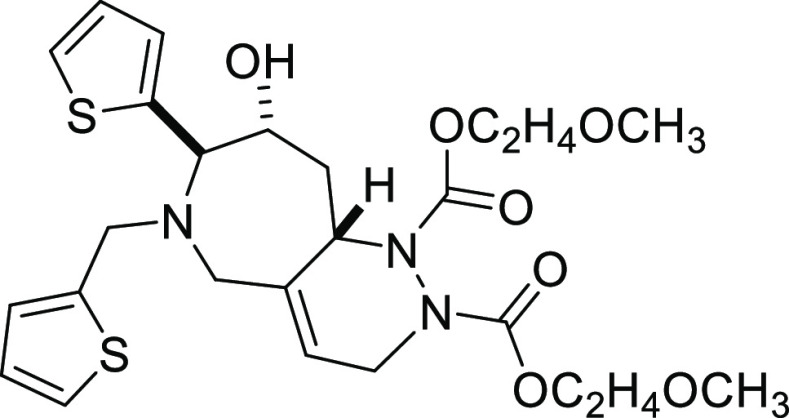



PR/RCM product **8b** (59 mg, 0.19 mmol, 1 equiv) was dissolved in DCM (0.5 M) and followed by that dibenzyl azodicarboxylate (60.98 mg, 0.20 mmol, 1.1 equiv) was added. The reaction was stirred in reflux for 0.5 h. The solvent was evaporated and purified as a white powder in 64% yield (4:1 *dr*) by column chromatography using ethyl acetate in petroleum ether 30% (isocratic flow). Major diastereomeric product: ^1^H NMR (500 MHz, CDCl_3_) δ 7.35–7.29 (m, 10 H), 7.22 (m, 2H), 7.07 (m, 1H), 7.00 (m, 1H), 6.90 (m, 2H), 5.47 (m, 1H), 5.23–5.08 (m, 4H), 4.92 (m, 1H), 4.49 (dd, *J* = 63.9, 16.5 Hz, 1H), 4.16–4.08 (m, 2H), 3.91–3.71 (m, 3H), 3.55–3.34 (m, 2H), 2.50–2.47 (m, 1H), 1.95–1.93 (m, 1H). ^13^C NMR (126 MHz, CDCl3) δ 155.38, 155.21, 135.90, 128.71, 128.64, 128.61, 128.44, 128.36, 128.29, 128.21, 128.14, 127.83, 127.82, 127.06, 126.56, 125.63, 125.61, 125.60, 125.55, 72.04, 69.09, 68.27, 68.04, 54.65, 53.12, 51.68, 43.17. HRMS-ESI (*m*/*z*): calculated for [M + H]^+^ C_33_H_34_N_3_O_5_S_2_, 616.1934; found, 616.1924.

## Supporting Information Available

Additional synthetic procedures and compound characterization; copies of ^1^H and ^13^C NMR spectra for all new small molecules (PDF)
X-ray crystallographic data for compound **5a** (CCDC: 2191710) (CIF)
X-ray crystallographic data for compound **9a** (CCDC: 2191711) (CIF)
X-ray crystallographic data for compound **9j** (CCDC: 2191712) (CIF)

## Supplementary Material

ao2c06585_si_001.pdf

ao2c06585_si_002.cif

ao2c06585_si_003.cif

ao2c06585_si_004.cif

## Abbreviations

3C-PR: three-component Petasis reaction
IMDA: intramolecular Diels–Alder
RCM: ring-closing metathesis reaction
ROM: ring-opening metathesis reaction
