# Supplementary material for: Petasis Sequence Reactions for the Scaffold-Diverse Synthesis of Bioactive Polycyclic Small Molecules
Source: ACS Omega. 2022 Dec 16;8(1):1168–81. doi: 10.1021/acsomega.2c06585 (PMC9835185; doi:10.1021/acsomega.2c06585)
Supplement: Supplementary file 1 — ao2c06585_si_001.pdf [file ao2c06585_si_001.pdf]

SUPPORTING INFORMATION

Petasis sequence reactions for the scaffold-diverse synthesis of bioactive polycyclic small molecules

Amrutha K. Avathan Veettil,[†,‡,§] Jan-Lukas Kirchhoff,[§] Lukas Brieger,[§] Carsten Strohmann,[§] and Peng Wu[†,‡,*]

[†]*Chemical Genomics Centre, Max Planck Institute of Molecular Physiology, Dortmund 44227, Germany*

[‡]*Department of Chemical Biology, Max Planck Institute of Molecular Physiology, Dortmund 44227, Germany*

[§]*Faculty of Chemistry and Chemical Biology, TU Dortmund University, Dortmund 44227, Germany*

*Correspondence: peng.wu@mpi-dortmund.mpg.de

Contents

Additional synthetic procedures and compound characterization……….………………….S02

X-ray crystallographic data for **5a** ……………………………………………………….... S06

X-ray crystallographic data for **9a** ……………………………………………………....... S 0 8

X-ray crystallographic data for **9j**…………………………………………………………S10

Predicited Molecular properties..……………………………………………………………S13

Predicted ADME properties…………………………………………………………………S14

Antiproliferative MTT assay protocol………………………………………………………S16

^{1}H and ^{13}C NMR spectra………………………………………………………….........S17

References……………………………………………………………………………….…..….S65

General methods

All commercially available solvents and reagents are purchased from Sigma Aldrich, TCI Chemical, or Fischer Scientific and used without further purification. All reactions were monitored by thin layer chromatography (TLC) and LC-MS Agilent 1260 II Infinity system equipped with a mass detector (column: InfinityLab Poroshell 120 EC-C18, 2.1x150, 2.7 µm). Appropriate gradient systems were applied by mixing H2O (+ 0.1% TFA) and acetonitrile (+ 0.1% TFA). Analytical thin-layer chromatography was carried out using Merck silica gel aluminum plates with F-254 indicator, visualized under UV light (at 254 nm), Iodine stain or dipping in potassium permanganate stain (1.5 g KMnO4, 10 g K2CO3, 1.25 mL of 10% aqueous NaOH solution and 200 mL of water). Starting materials **1c**, **2**, **6a**, **6b** were prepared following the known literature procedures and were in agreement with the literature data. The products were purified by column chromatography over silica gel (Merck 60 particle size 0.040 - 0.063 mm). Solvents for chromatography were laboratory grade. All ^{1}H and ^{13}C NMR spectra were recorded on a Bruker DRX400 (400 MHz), DRX500 (500MHz), DRX600 (600 MHz) and DRX700 (700 MHz) spectrometers in $CDCl_3$ and $(CD_3)_2SO$. Data are reported in the following order: chemical shift in ppm; multiplicities are indicated s (singlet), d (doublet), t (triplet), q (quartet), dd (doublet of doublet), ddt (doublet of doublet of triplets) and m (multiplet). Coupling constants (*J*) are given in Hertz (Hz). High resolution mass spectra were recorded on an LTQ Orbitrap mass spectrometer coupled to an Accela HPLC System (HPLC column: Hypersyl GOLD, 50 mm × 1 mm, 1.9 µm). Chemical yields refer to isolated pure substances.

Synthetic procedures and compound characterization

A. Synthesis of olefinated building blocks

N-Allyl-*N*-(2-furylmethyl) amine (1c)

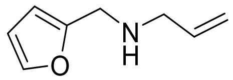

The compound was synthesized according to procedure from reported literature.[1] Using, allylamine (0.98 g, 17.17 mmol), furfural (1.50 g, 15.61 mmol) and $MgSO_4$ (1.88 g) in DCM (12 mL). The crude imine formed from the reaction was then used in next step in 30 mL methanol, and $NaBH_4$ (0.42 g, 11.10 mmol). The resultant crude amine was purified by column chromatography to afford pure *N*-allyl-*N*-(2-furylmethyl) amine (**1c**), 1.65 g, 81% yield. ^{1}H NMR (600 MHz, DMSO-*d* ^{6}) δ 7.54 (m, 1H), 6.37 (dd, *J* = 3.0, 1.8 Hz, 1H), 6.21 (d, *J* = 3.0 Hz, 1H). 5.85-5.78 (m, 1H), 5.16-5.03 (m, 2H), 3.63 (s, 2H), 3.12-3.11 (m, 2H), 2.12 (bs, 1H). Spectroscopic data were in accordance with the reported literature.[1]

5-Allyl-2,2-dimethyl-1,3-dioxolan-4-ol (2)

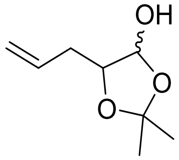

5-Allyl-2,2-dimethyl-1,3-dioxolan-4-ol (**2**) was synthesized according to literature procedure.[2] First, 2-hydroxypent-4-enoic acid was obtained from glyoxylic acid (3.00 g, 32.59 mmol), allyl bromide (6.81 g, 52.15 mmol) and fine flakes of freshly cut indium (3.78 g, 0.174 mmol) in a mixture of THF: H2O (2:1) (60 mL). The resulting 2 -hydroxypent-4-enoic acid was used in the next step without further purification (2.23 g, 59% yield). ^{1}H NMR (400 MHz, $CDCl_3$) δ 5.87-5.77

(m, 1H), 5.23-5.17 (m, 2H), 4.36 (dd, J = 6.8, 4.8 Hz, 1H), 2.68-2.46 (m, 2H). Spectroscopic data were in accordance with the reported literature.[2]

Then, 5-allyl-2,2-dimethyl-1,3-dioxolan-4-one was synthesized from 2-hydroxypent-4-enoic acid (2.21 g, 19.07 mmol), 2,2-dimethoxypropane (15.87 g, 152.54 mmol) and pyridine p-toluenesulfonate (0.96 g, 3.8 mmol) in acetone (124 mL). Purified the crude reaction mixture to get the pure product which was used for the next step (2.3 g, 77% yield). ^{1}H NMR (400 MHz, $CDCl_3$) δ 5.82 (ddt, J = 17.1, 10.2, 6.9 Hz, 1H), 5.33 – 5.14 (m, 2H), 4.47 (dd, J = 6.7, 4.3 Hz, 1H), 2.71 – 2.59 (m, 1H), 2.56 – 2.43 (m, 1H), 1.61 (s, 3H), 1.55 (s, 3H). Spectroscopic data were in accordance with the reported literature.[2]

Finally, 5-allyl-2,2-dimethyl-1,3-dioxolan-4-ol (**2**) was synthesized from a solution of 5-allyl-2,2-dimethyl-1,3-dioxolan-4-one (2.30 g, 14.73 mmol) in toluene (60 mL) using DIBAL-H (18.59 mL, 17.52 mmol, 25% DIBAL-H in toluene). The final product (**2**) was immediately used for reactions without further purification (2.08 g, 89 % yield).

***N*-Propargyl-thiophenemethylamine (6a)**

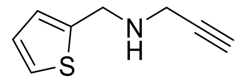

The compound was synthesized according to reported procedure.[3] Using, thiophene methylamine (2.0 g, 17.67 mmol), propargylbromide 80% (2.63 g, 17.67 mmol) and K_2CO_3 in THF. Purified the crude amine by column chromatography to afford the pure product (1.63 g, 60.91% yield). ^{1}H NMR (500 MHz, $CDCl_3$) δ 7.24 (dd, J = 5.0, 1.0 Hz, 1H), 6.99-6.98 (m, 1H), 6.96 (dd, J = 5.0, 3.5 Hz, 1H), 4.11 (d, J = 0.7 Hz, 2H), 3.47 (d, J = 2.5 Hz, 2H), 2.27 (t, J = 2.5 Hz, 1H). Spectroscopic data were in accordance with the reported literature.[3]

N-(Thiophen-2-ylmethyl)but-2-yn-1-amine (6b)

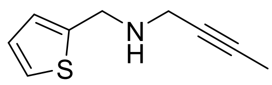

The compound was synthesized by procedure adapted from reported literature.[3] Thiophene methylamine 0.70 g (6.18 mmol) and K_2CO_3 1.734 g (12.37 mmol) was added to THF 42 mL, cool down the mixture to zero degree. A solution of 1-bromo-2-butyne 822.487 (6.18 mmol) in THF 42 mL was added drop-wise to the mixture over a period of 2 h at 0 ˚C. Stirred the reaction mixture at room temperature for 12 h. The mixture was concentrated *in vacuo*. The crude amine was purified by column chromatography on silica gel using petroleum ether and ethyl acetate (355 mg, 35% yield). ^{1}H NMR (400 MHz, $CDCl_3$) δ 7.22 (dd, J = 4.8, 1.2 Hz, 1H), 6.97-6,.94 (m, 2H), 4.07 (s, 2H), 3.41 (q, J = 2.3 Hz, 2H), 1.84 (t, J = 2.4 Hz, 3H), 1.77 (s, 1H).

X-Ray diffraction parameters and data for 5a, 9a, 9j:

1. **5a (**CCDC number: 2191710)

Single crystals of **5a** were obtained by crystallization from dichloromethane and diethyl ether at room temperature. The asymmetric unit contains two independent molecules. Disorder at the thiophene substituent was solved in structure **5a** (Figure S1).

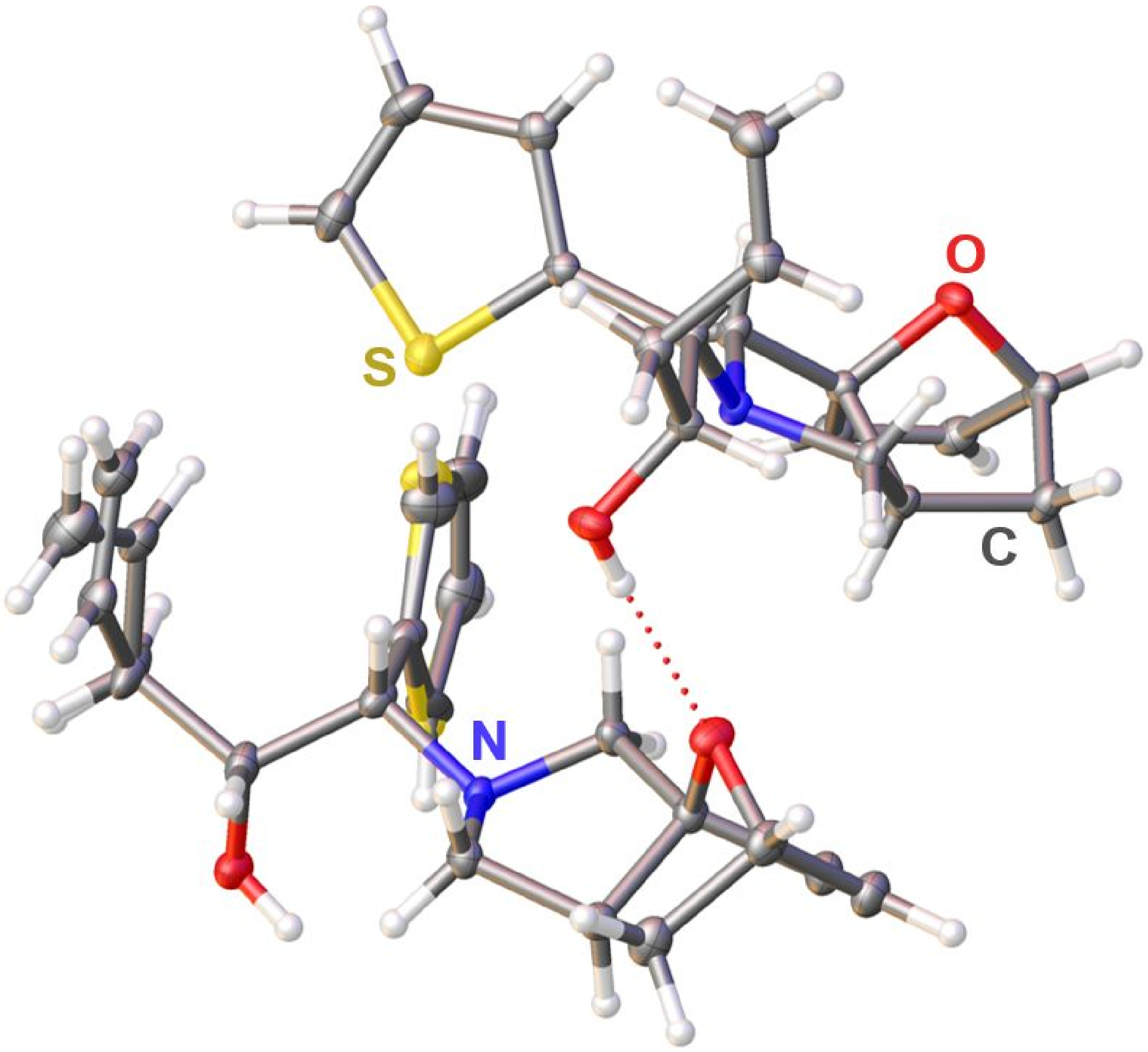

Figure S1. Single crystal X-ray structure of **5a**.

**Compound ID**	**mo_B2684**
Formula	$C_{17}H_{21}NO_2S$
$D_{calc.}$/ g cm^{-3}	1.327
m/mm^{-1}	0.217
Formula Weight	303.41
Colour	colourless
Shape	plate
Size/mm^3	0.66×0.28×0.12
T/K	100.00
Crystal System	monoclinic
Space Group	$P2_1/c$
a/Å	9.9071(4)
b/Å	15.6821(5)
c/Å	19.5899(7)
a/°	90
b/°	93.5510(10)
g/°	90
V/Å^3	3037.72(19)
Z	8
Z'	2
Wavelength/Å	0.71073
Radiation type	MoK_a
Q_{min}/°	2.060
Q_{max}/°	31.610
Measured Refl.	202138
Independent Refl.	10174
Reflections with I > 2(I)	8849
R_{int}	0.0560
Parameters	419
Restraints	0
Largest Peak	0.811
Deepest Hole	-0.502
GooF	1.057
wR_2 (all data)	0.1137
wR_2	0.1082

R_1 (all data)	0.0486
R_1	0.412

Note regarding the crystal structure of 5a:

The structure of compound **5a** shows two level B alerts of the "Hirshfeld Test Diff" type, indicating an unusual bond length between carbon atoms C10 and C11, as well as C11 and C12. The comparison with the other molecule in the unit cell shows that the sulfur atom of the thiophene substituent exhibits a disorder. This is probably also present in the other molecule in the unit cell in small parts, which cannot be further refined crystallographically with the remaining electron density. Therefore, the two C=C-bonds are slightly longer, causing the above-mentioned level B alerts, which do not interfere with the structural verification for compound **5a**.

2. **9a** (CCDC number: 2191711)

Single crystals of **9a** were obtained from crystallization in dichloromethane and acetonitrile at room temperature. Disorder at the thiophene substituent was solved in structure **9a** (Figure S2).

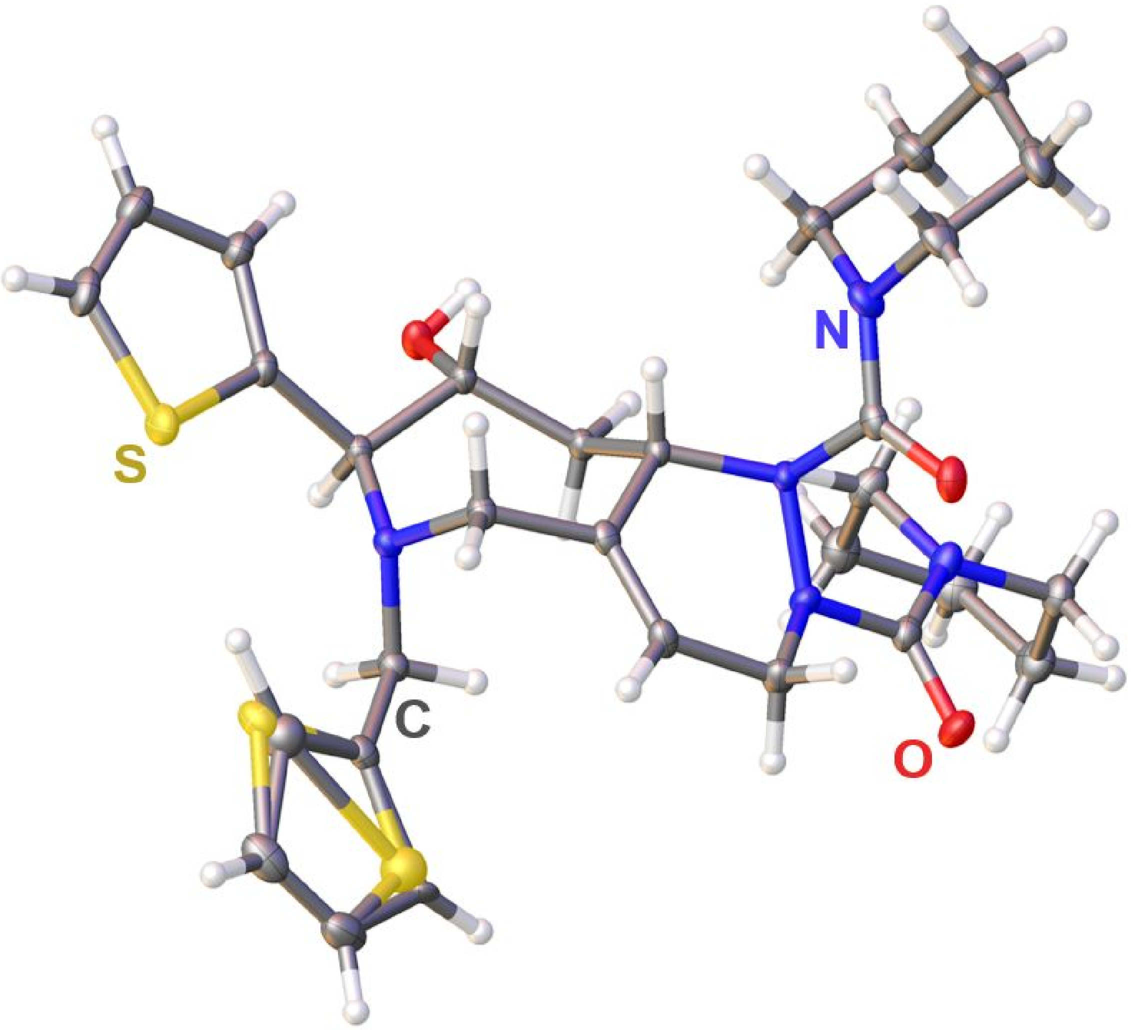

Figure S2. Single crystal X-ray structure of **9a**.

**Compound ID**	**mo_B2666_0m**
Empirical formula	$C_{29}H_{39}N_5O_3S_2$

Formula weight	569.77
Temperature/K	100.00
Crystal system	monoclinic
Space group	$P2_1/n$
a/Å	9.725(2)
b/Å	13.562(3)
c/Å	22.111(5)
α/°	90
β/°	98.704(10)
γ/°	90
Volume/Å^3	2882.7(11)
Z	4
ρ_{calc}g/cm^3	1.313
μ/mm^{-1}	0.224
F(000)	1216.0
Crystal size/mm^3	0.216 × 0.192 × 0.061
Radiation	MoKα (λ = 0.71073)
2Θ range for data collection/°	3.728 to 60.192
Index ranges	$-13 \leq h \leq 13$, $-18 \leq k \leq 19$, $-31 \leq l \leq 31$
Reflections collected	62861
Independent reflections	8474 [R_{int} = 0.0458, R_{sigma} = 0.0296]
Data/restraints/parameters	8474/0/377
Goodness-of-fit on F^2	1.062
Final R indexes [I>=2σ (I)]	R_1 = 0.0417, wR_2 = 0.0920
Final R indexes [all data]	R_1 = 0.0578, wR_2 = 0.1013
Largest diff. peak/hole / e Å^{-3}	0.31/-0.47

3. **9j** (CCDC number: 2191712)

Single crystals of **9j** were obtained from crystallization in dichloromethane and acetonitrile at room temperature. Disorder at the morpholine substituent was solved in structure **9j** (Figure S3).

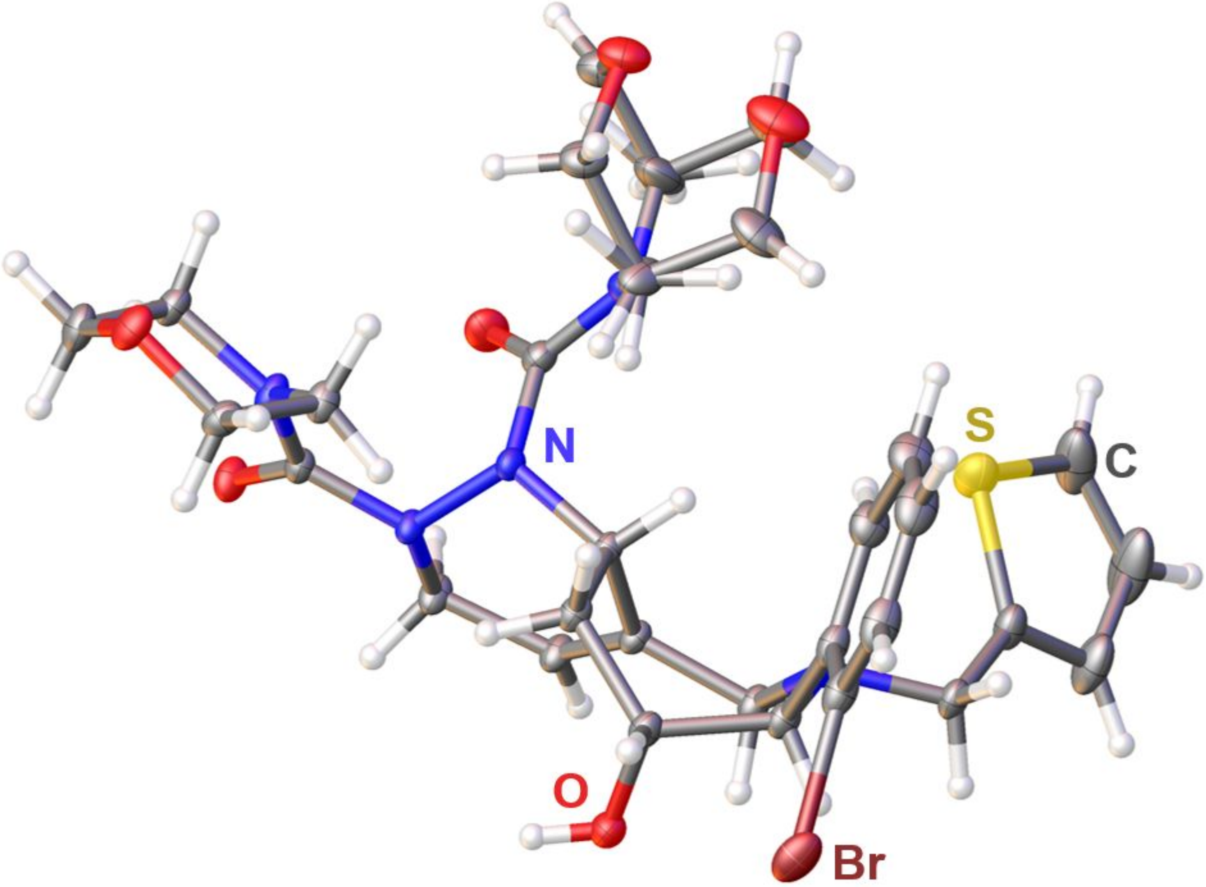

Figure S3. Single crystal X-ray structure of **9j**.

**Compound ID**	**mo_B2498_0m**
Empirical formula	$C_{29}H_{35}BrN_5O_5S$
Formula weight	645.59
Temperature/K	100.00
Crystal system	triclinic
Space group	P-1
a/Å	10.1270(3)
b/Å	12.4110(5)
c/Å	12.9814(5)
α/°	94.3570(10)
β/°	105.8450(10)
γ/°	109.4430(10)
Volume/Å^3	1454.83(9)
Z	2
ρ_{calc}g/cm^3	1.474
μ/mm^{-1}	1.533
F(000)	670.0
Crystal size/mm^3	0.264 × 0.193 × 0.125
Radiation	MoKα (λ = 0.71073)
2Θ range for data collection/°	3.54 to 66.552
Index ranges	$-15 \leq h \leq 14, -19 \leq k \leq 19, -19 \leq l \leq 19$
Reflections collected	79554
Independent reflections	10929 [R_{int} = 0.0447, R_{sigma} = 0.0304]
Data/restraints/parameters	10929/0/399
Goodness-of-fit on F^2	1.025
Final R indexes [I>=2σ (I)]	R_1 = 0.0400, wR_2 = 0.1019
Final R indexes [all data]	R_1 = 0.0529, wR_2 = 0.1088
Largest diff. peak/hole / e Å^{-3}	1.27/-0.87

Table S1. Predicted molecular properties*:

**Compound**	**5a**	**5b**	**5c**	**9a**	**9c**	**9e**	**9g**	**9h**	**9j**	**9l**
Molecular Weight	303.42	311.425	297.47	569.78	573.73	547.73	519.68	492.61	646.60	551.67
LogP	2.87	3.42	2.97	4.55	2.43	5.02	4.13	2.76	3.29	2.69
Rotatable bonds	5	5	5	3	3	7	7	1	3	11
tPSA	32.70	32.70	32.70	70.56	89.03	82.55	82.55	72.41	89.03	101.02

Table S2. Predicted ADME properties*:

Compound	5a	5b	5c	9a	9c	9e	9g	9h	9j	9l
**Absorption**										
Water solubility log mol/L	-2.31	-2.37	-2.41	-4.98	-4.46	-5.82	-5.47	-4.11	-4.67	-4.78
Caco2 permeability log Papp in 10^{-6} cm/s	1.52	1.36	1.52	0.99	0.91	0.54	0.57	0.83	1.03	0.84
Intestinal absorption (human) % Absorbed	89.38	90.64	90.53	88.04	91.23	85.34	87.25	92.58	91.77	85.50
Skin Permeability log Kp	-2.79	-2.78	-2.75	-2.86	-2.86	-2.80	-2.86	-2.75	-2.82	-2.81
**Distribution**										
VDss (human)	0.88	1.10	1.08	0.86	0.36	0.20	0.32	0.57	0.48	0.23
Fraction unbound (human)	0.62	0.59	0.60	0.08	0.19	0	0.03	0.10	0.15	0.18
BBB permeability	0.67	0.18	0.21	-1.09	-1.22	-0.48	-0.62	-0.45	-1.26	-1.24
CNS permeability	-2.86	-2.8	-2.77	-1.86	-2.49	-1.43	-1.86	-2.05	-2.30	-3.22
**Metabolism**										
CYP2D6 substrate	No	No	No	Yes	Yes	No	No	No	Yes	No
CYP3A4 substrate	Yes	Yes	Yes	Yes	Yes	Yes	Yes	Yes	Yes	Yes

CYP1A2 inhibitior	No	Yes	Yes	No	No	No	No	Yes	No	No
CYP2C19 inhibitior	No	No	No	No	No	No	No	No	No	No
CYP2C9 inhibitior	No	No	No	No	No	Yes	Yes	No	No	No
CYP2D6 inhibitior	Yes	Yes	Yes	No	No	No	No	Yes	No	No
CYP3A4 inhibitior	No	No	No	Yes	No	Yes	Yes	Yes	No	Yes
**Excretion**										
Total Clearance log ml/min/kg	1.18	1.06	1.05	0.48	0.45	0.15	0.51	0.69	0.22	0.63
Renal OCT2 substrate	No	Yes	No	Yes	Yes	Yes	Yes	No	Yes	Yes

*Molecular and ADME were predicted using pkCSM tool https://biosig.lab.uq.edu.au/pkcsm/prediction[4] and molinspiration cheminformatics free web services.

Anti-proliferative MTT assay on cancer cells:

An MTT ((3-(4,5-dimethylthiazol-2-yl)-2,5-diphenyltetrazolium bromide) assay was performed to evaluate the anti-proliferative activities of synthesized compounds. Cancer cells (obtained from DSMZ, German Collection of Microorganisms and Cell Cultures, Braunschweig) were seeded in 96-well plates (4000 cells/well) and incubated for 24 h. The old medium was then discarded and the media with compounds were added to the 96-well plates. Data were normalized to the medium with 1% DMSO. After incubating for 72 h, MTT solution (5 mg/mL, 20 µL) was added per well in dark and the resulting cells were incubated for 4 h. The old medium with MTT solution was then removed and 150 µL DMSO was added per well, the absorbance of which was measured at 492 nm by a TECAN plate reader.

^{1}H and ^{13}C NMR spectra:

Figure S4. ^{1}H NMR (600 MHz, DMSO-d_6) of *N*-Allyl-*N*-(2-Furylmethyl) amine (**1a**)

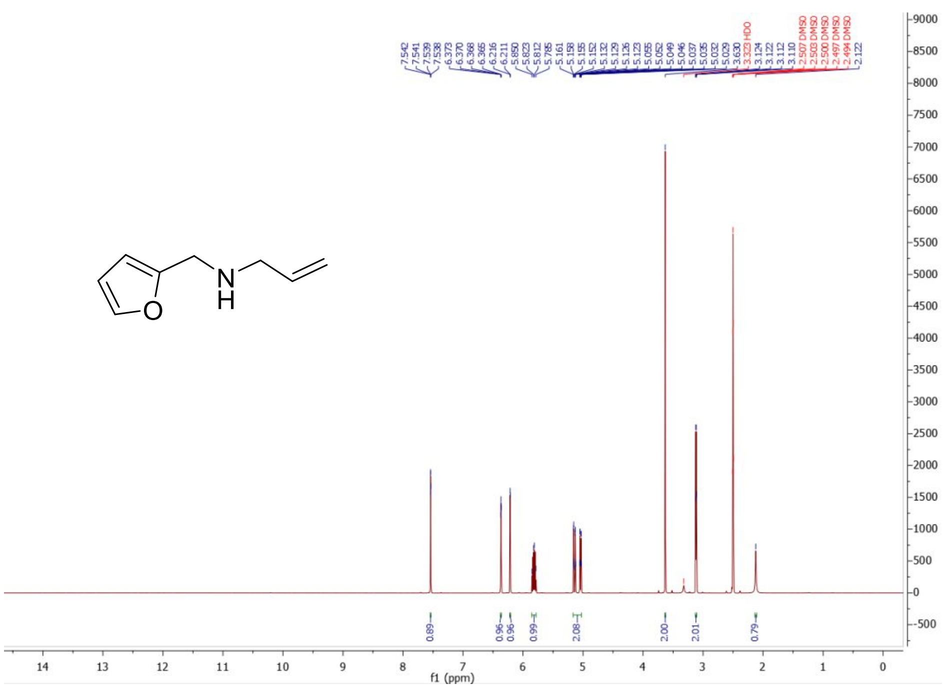

Figure S5. ^{1}H NMR (400 MHz, $CDCl_3$) of 2-Hydroxypent-4-enoic acid

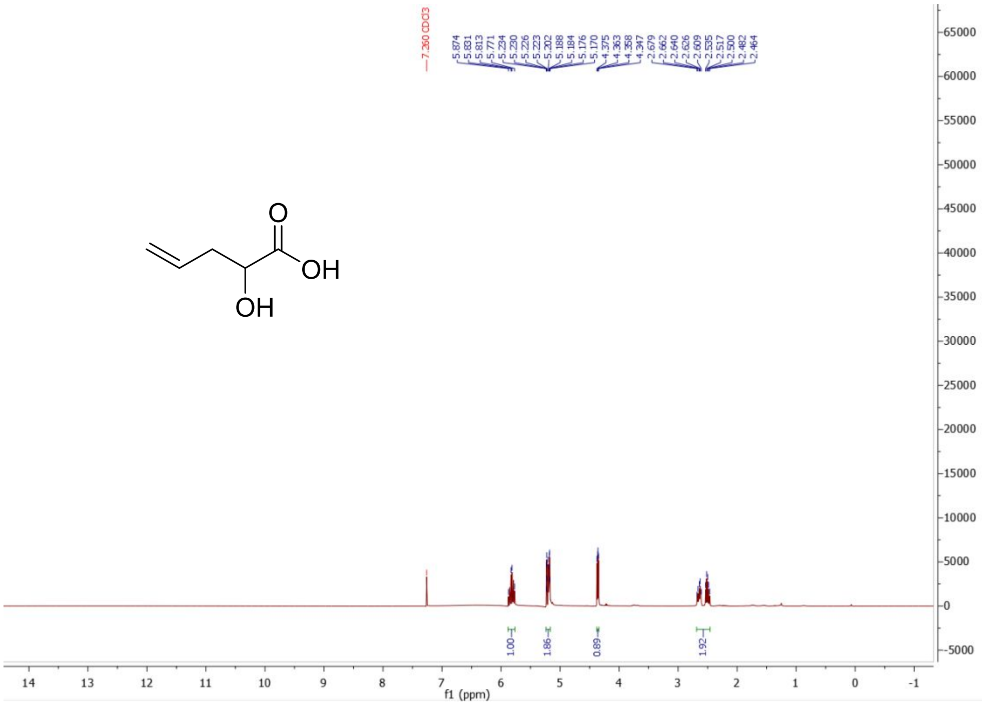

Figure S6. ^{1}H NMR (400 MHz, $CDCl_3$) of 5-Allyl-2,2-dimethyl-1,3-dioxolan-4-one

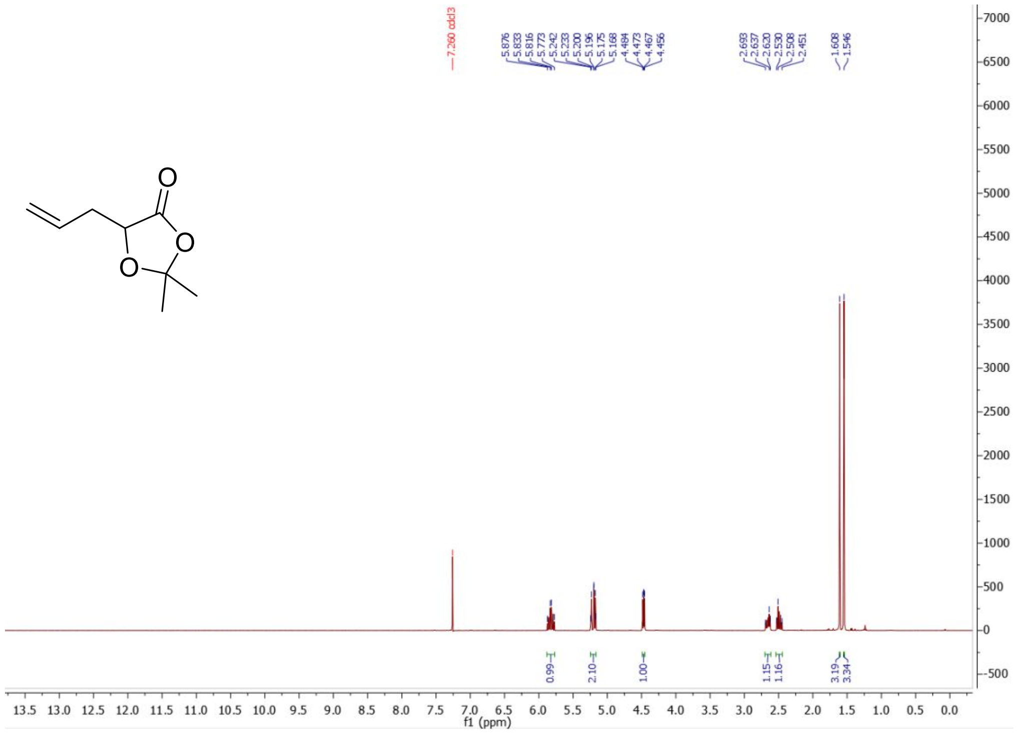

Figure S7. ^{1}H NMR (500 MHz, $CDCl_3$) of *N*-Propargyl-thiophenemethylamine (**6a**)

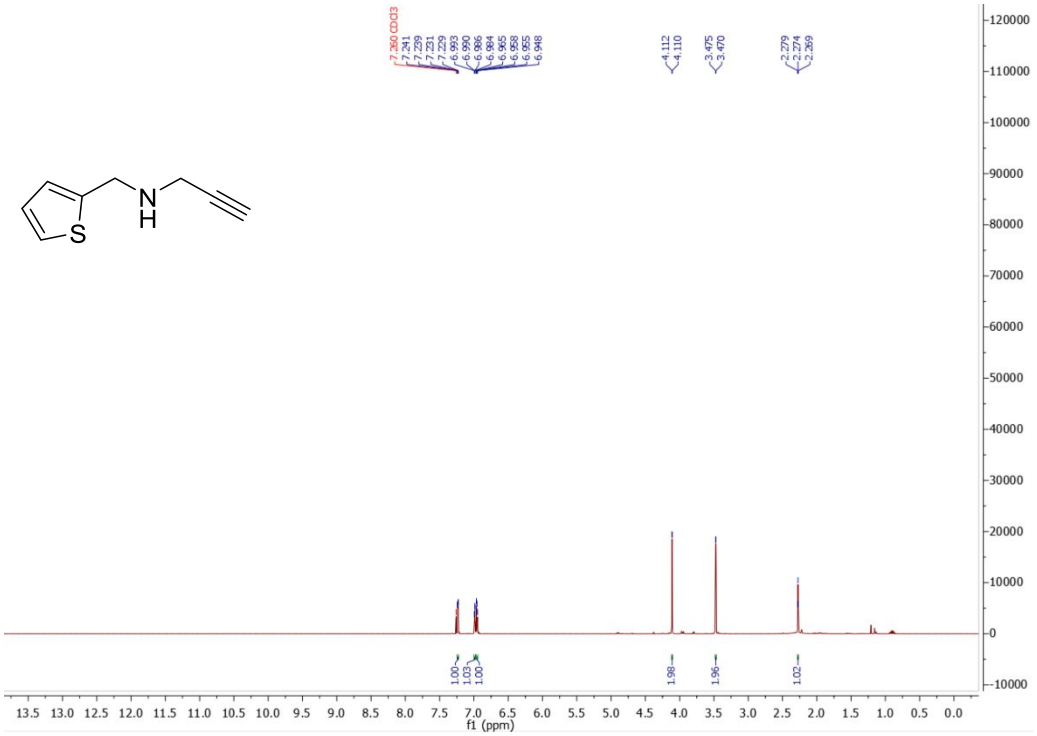

Figure S8. ^{1}H NMR (400 MHz, $CDCl_3$) of *N*-(Thiophen-2-ylmethyl)but-2-yn-1-amine (**6b**)

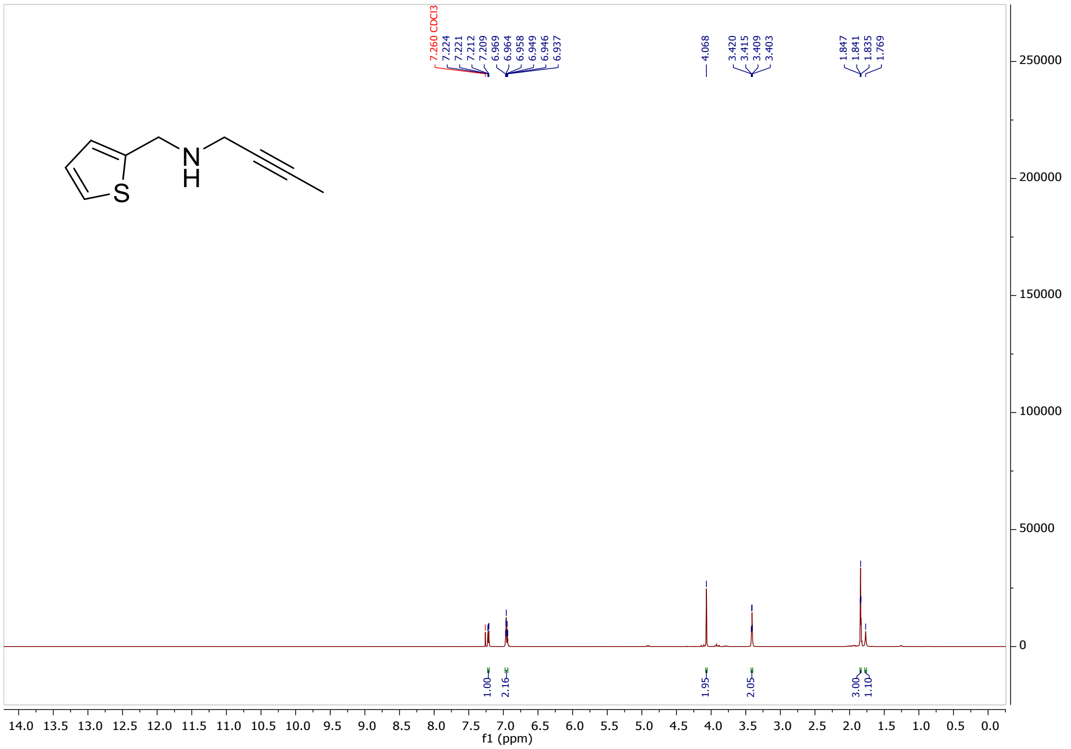

Figure S9. ^{1}H NMR (500 MHz, $CDCl_3$) of **4a**

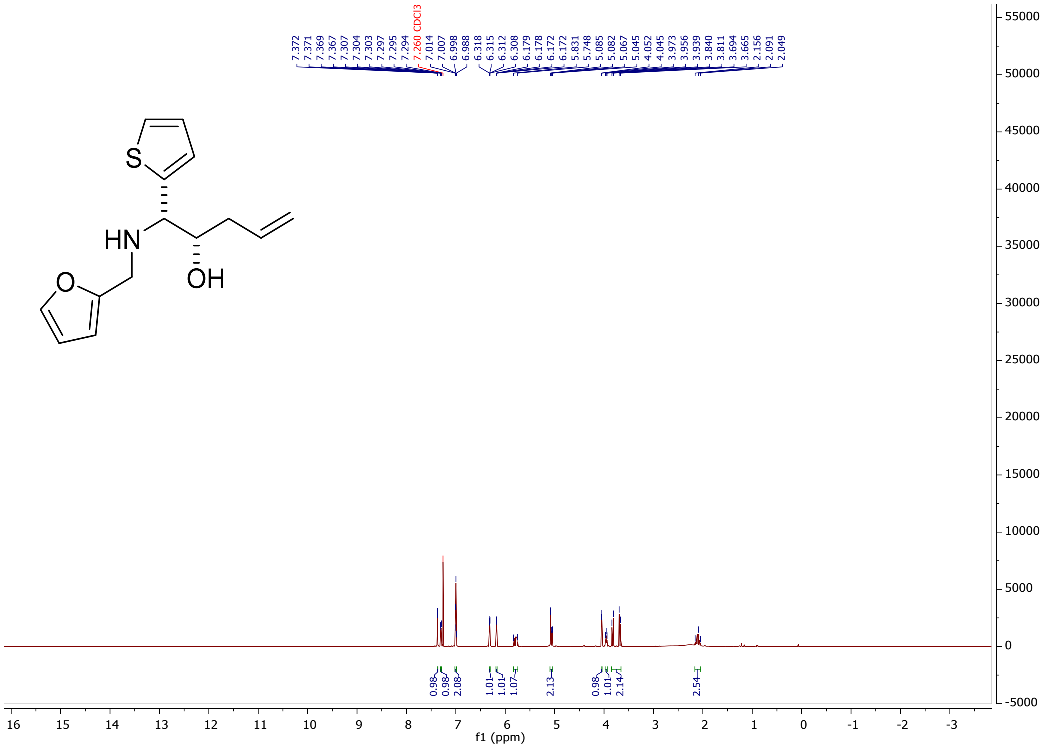

Figure S10. Expanded ^{1}H NMR (500 MHz, $CDCl_3$) of **4a**

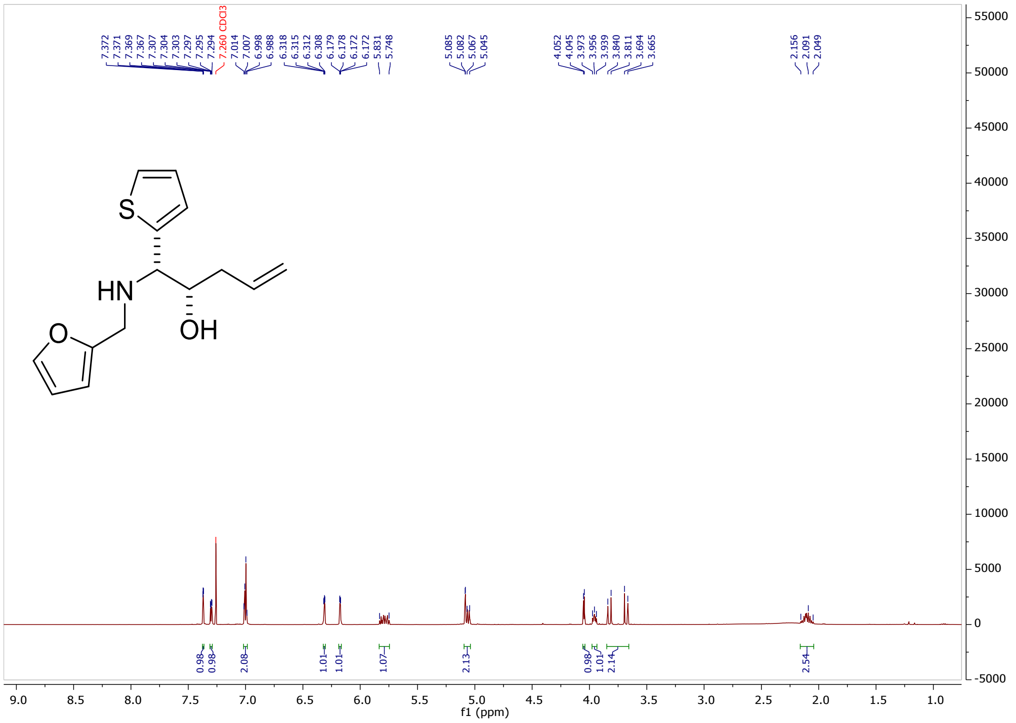

Figure S11. ^{13}C NMR (126 MHz, $CDCl_3$) of **4a**

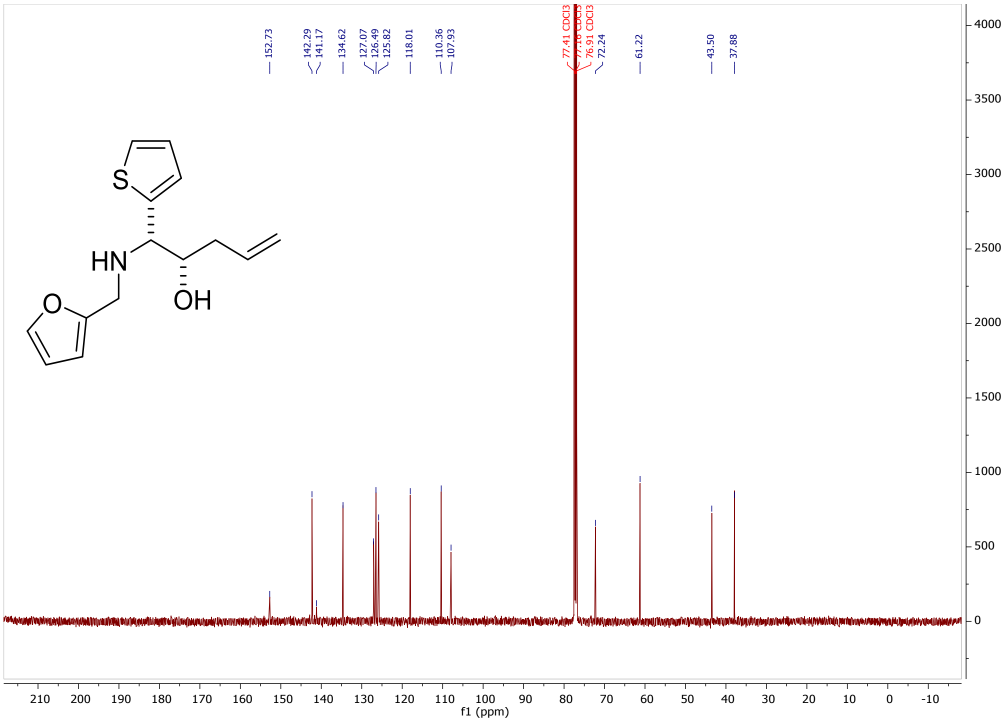

Figure S12. ^{1}H NMR (600 MHz, $CDCl_3$) of **4b**

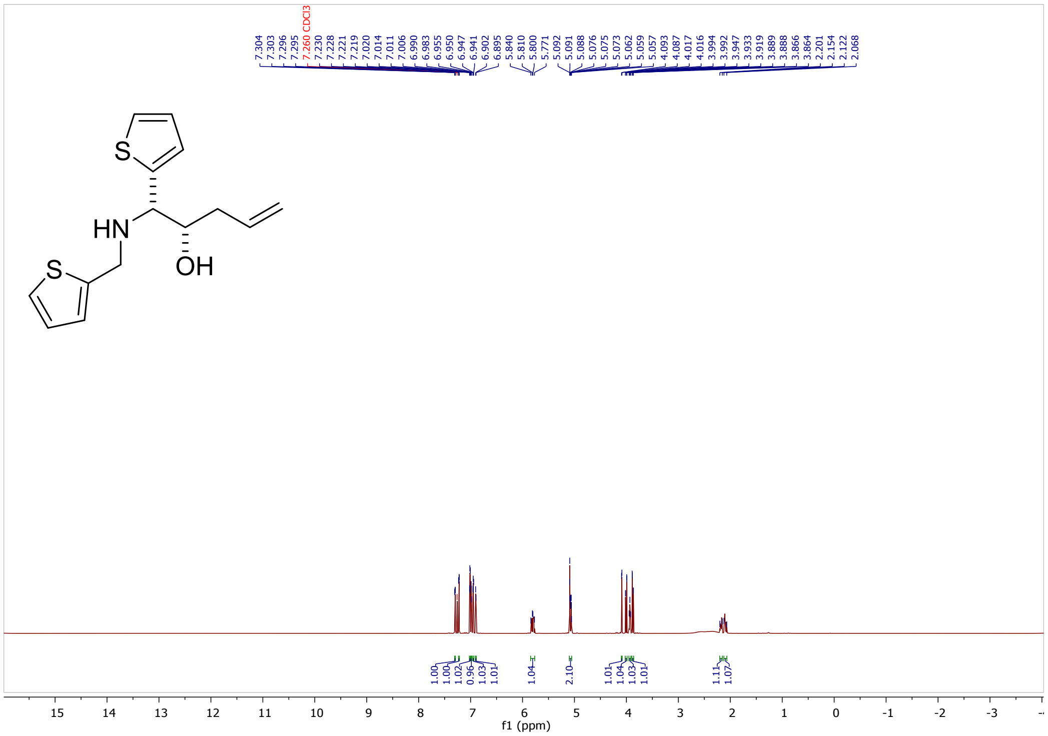

Figure S13. Expanded ^{1}H NMR (600 MHz, $CDCl_3$) of **4b**

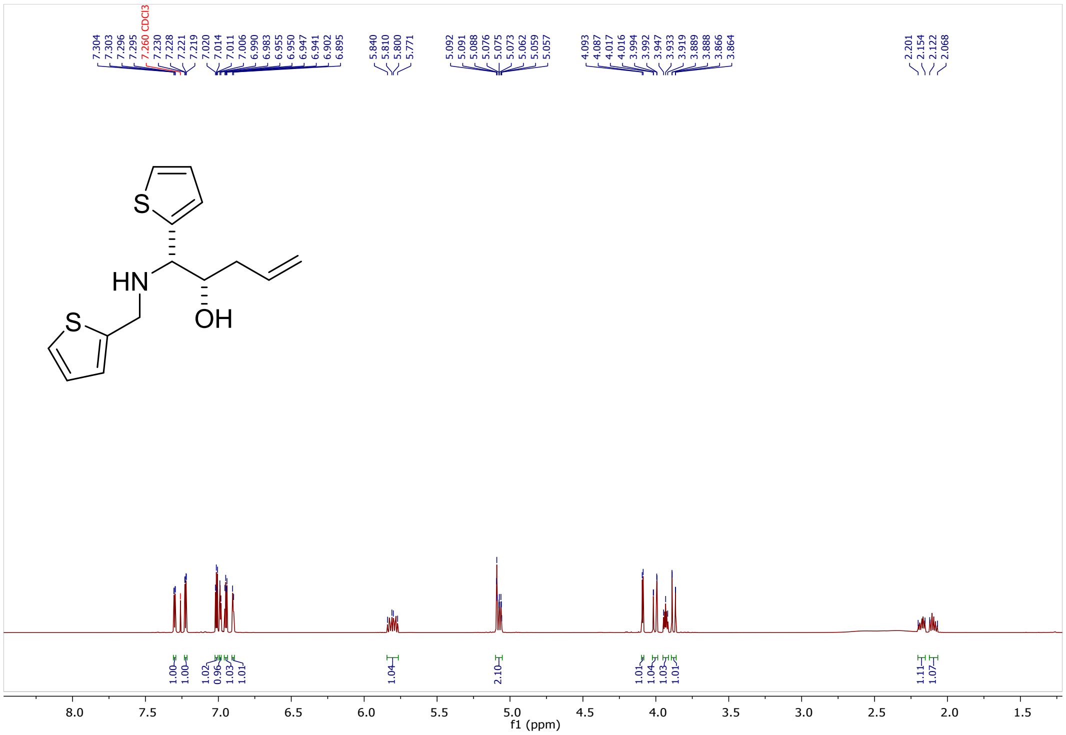

Figure S14. ^{13}C NMR (151 MHz, $CDCl_3$) of **4b**

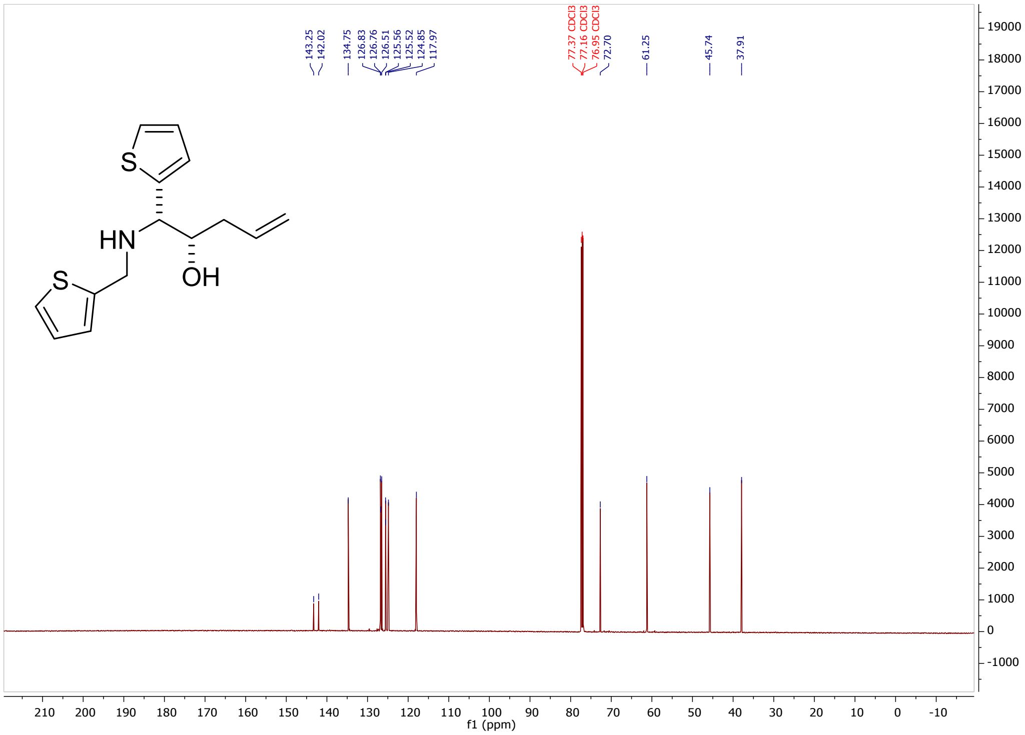

Figure S15. ^{1}H NMR (700 MHz, DMSO-d_6) of **4c**

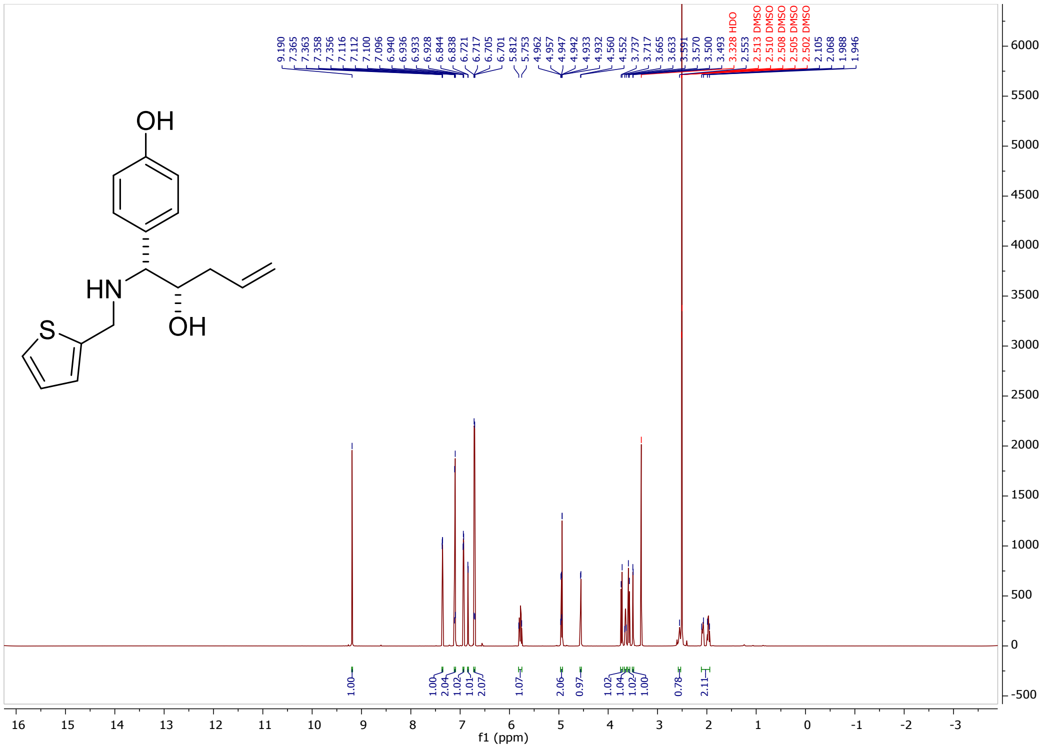

Figure S16. Expanded ^{1}H NMR (700 MHz, DMSO-d_6) of **4c**

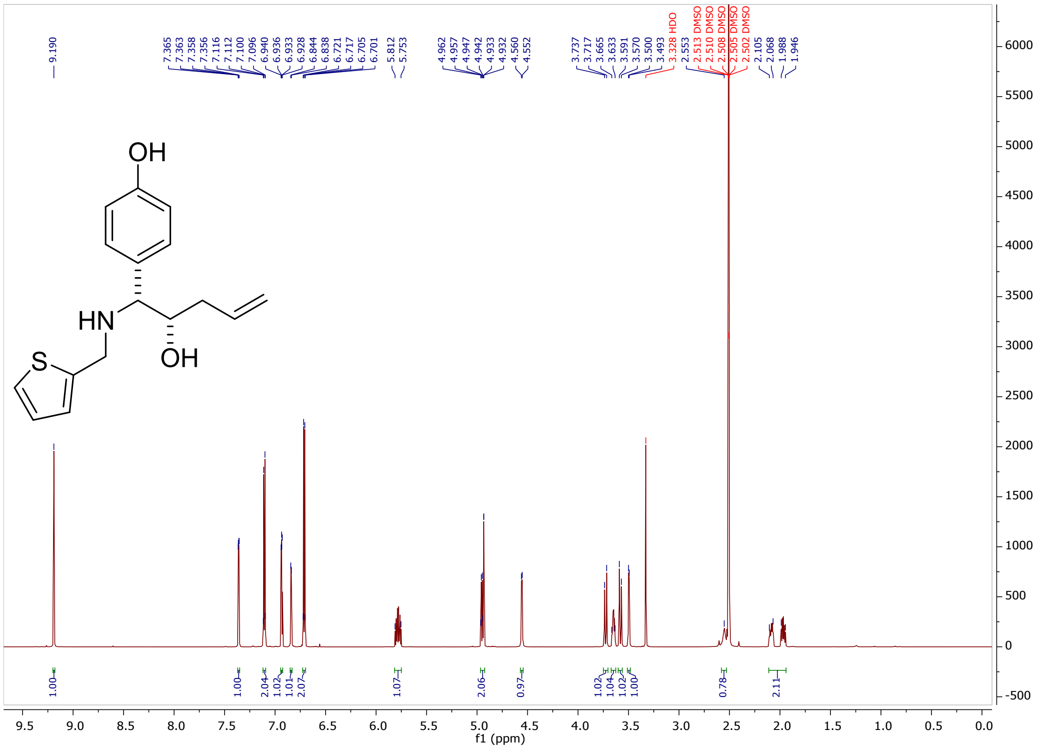

Figure S17. ^{13}C NMR (176 MHz, DMSO-d_6) of **4c**

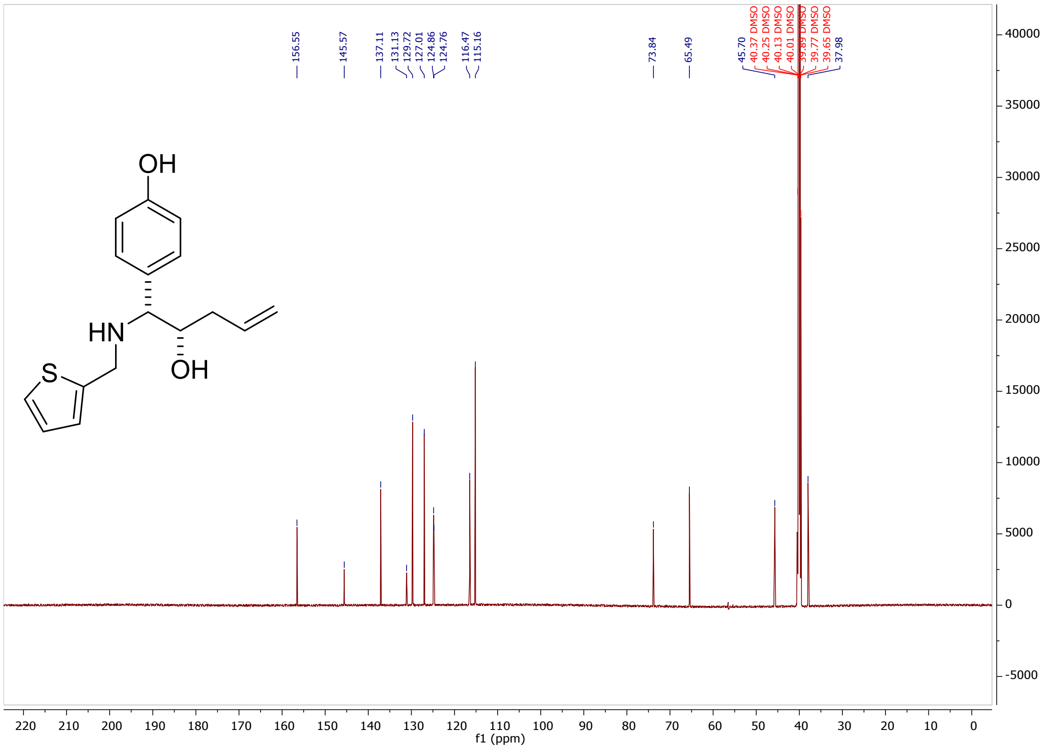

Figure S18. ^{1}H NMR (500 MHz, $CDCl_3$) **(4d)**

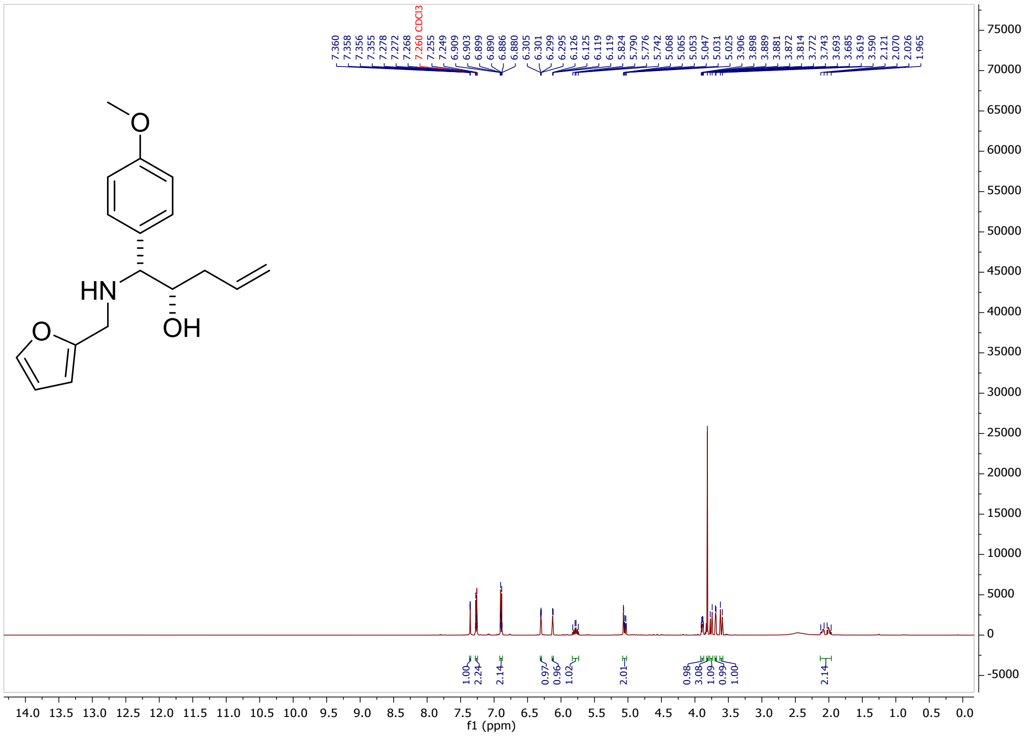

Figure S19. Expanded ^{1}H NMR (500 MHz, $CDCl_3$) **(4d)**

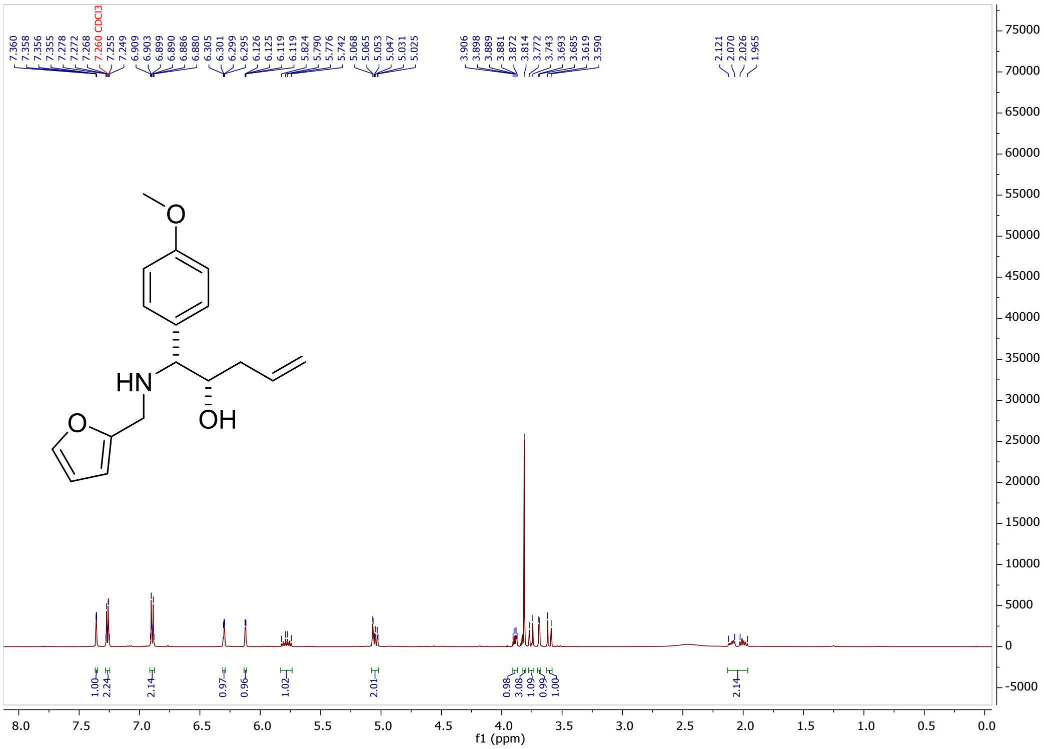

Figure S20. ^{13}C NMR (126 MHz, $CDCl_3$) **(4d)**

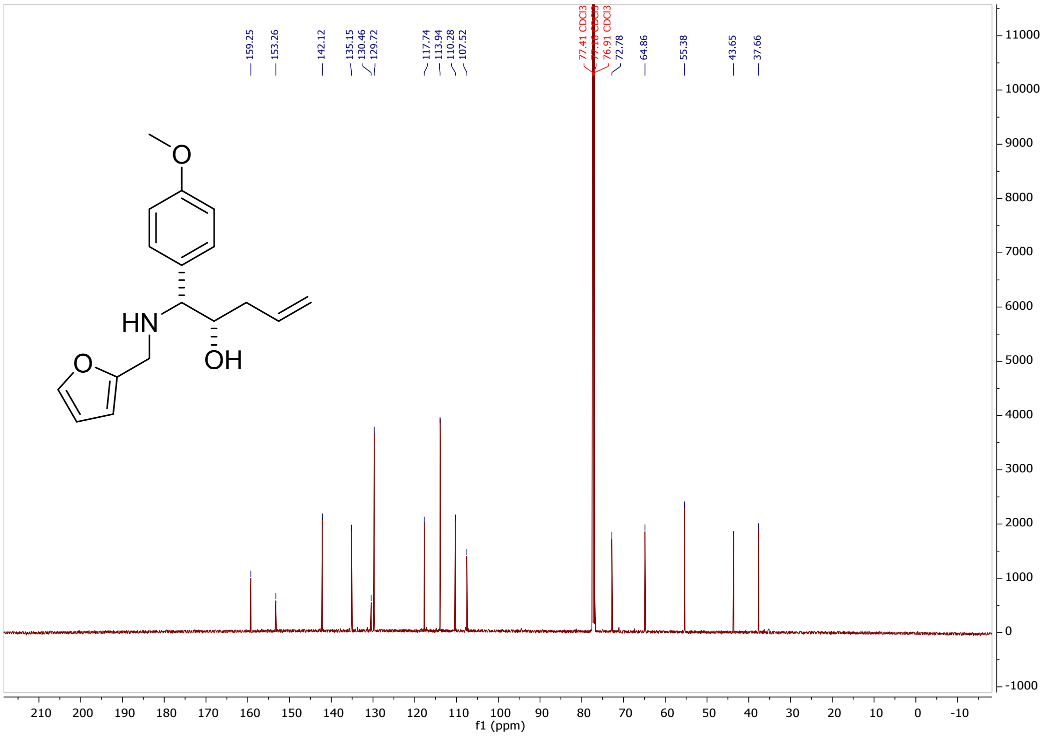

Figure S21. ^{1}H NMR (500 MHz, $CDCl_3$) **(4e)**

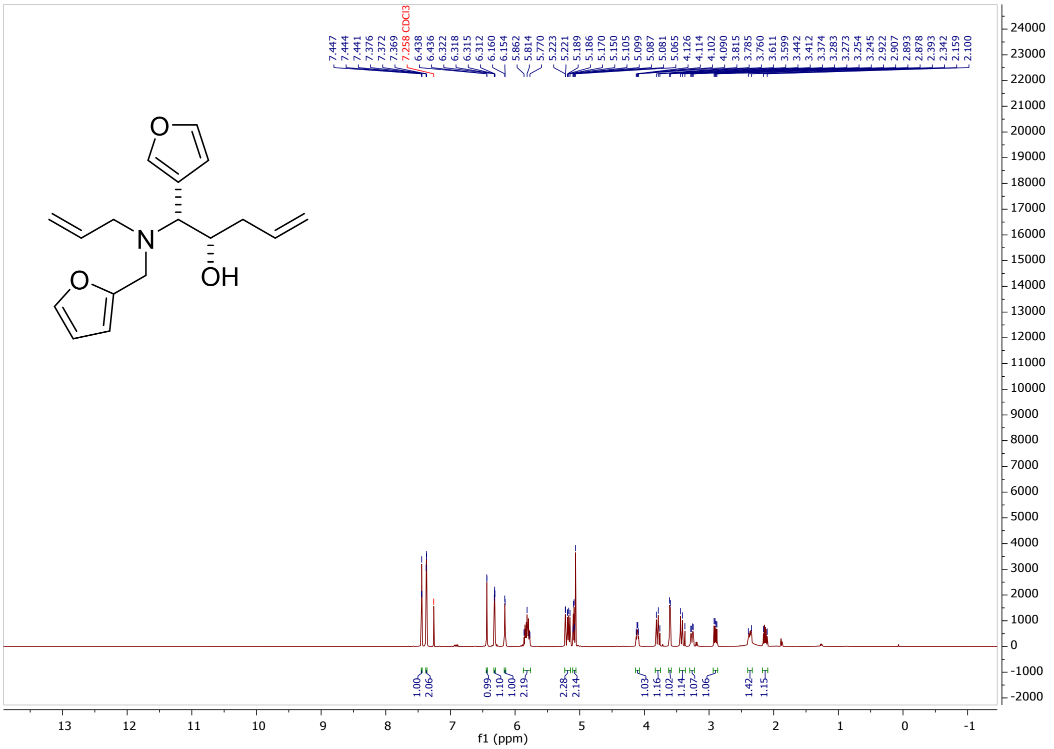

Figure S22. Expanded ^{1}H NMR (500 MHz, $CDCl_3$) **(4e)**

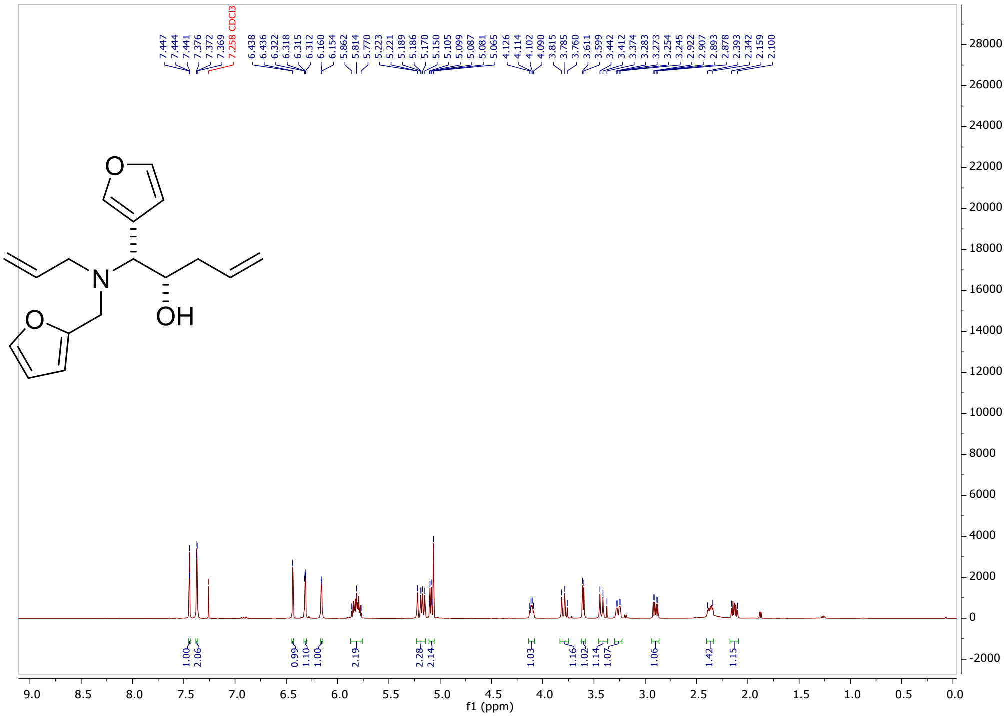

Figure S23. ^{13}C NMR (126 MHz, $CDCl_3$) **(4e)**

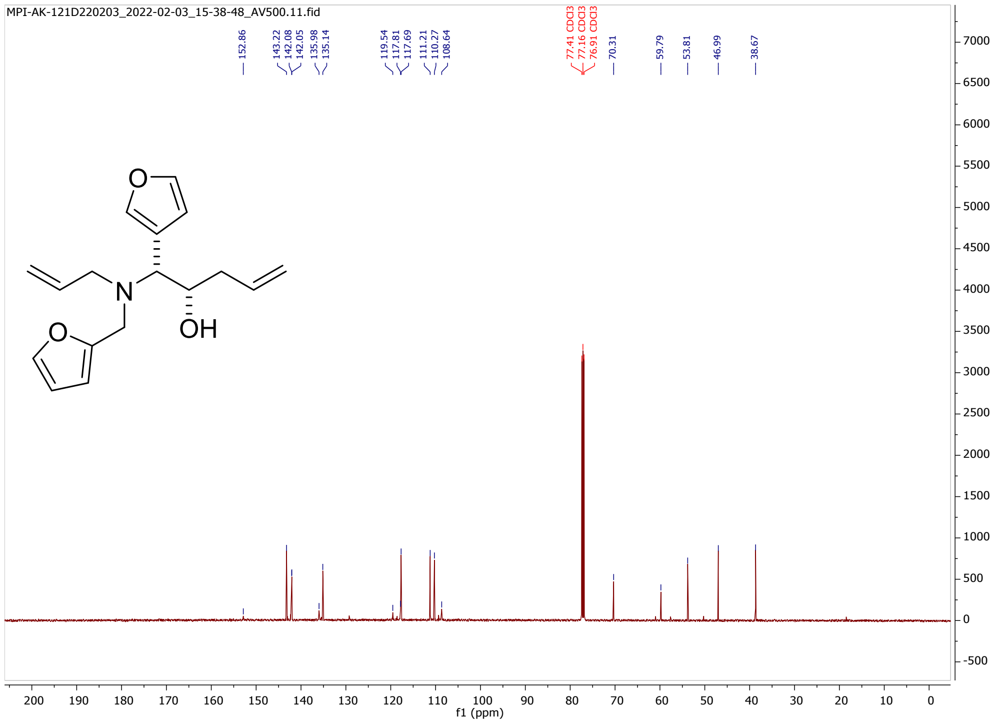

Figure S24. ^{1}H NMR (600 MHz, $CDCl_3$) of **(4f)**

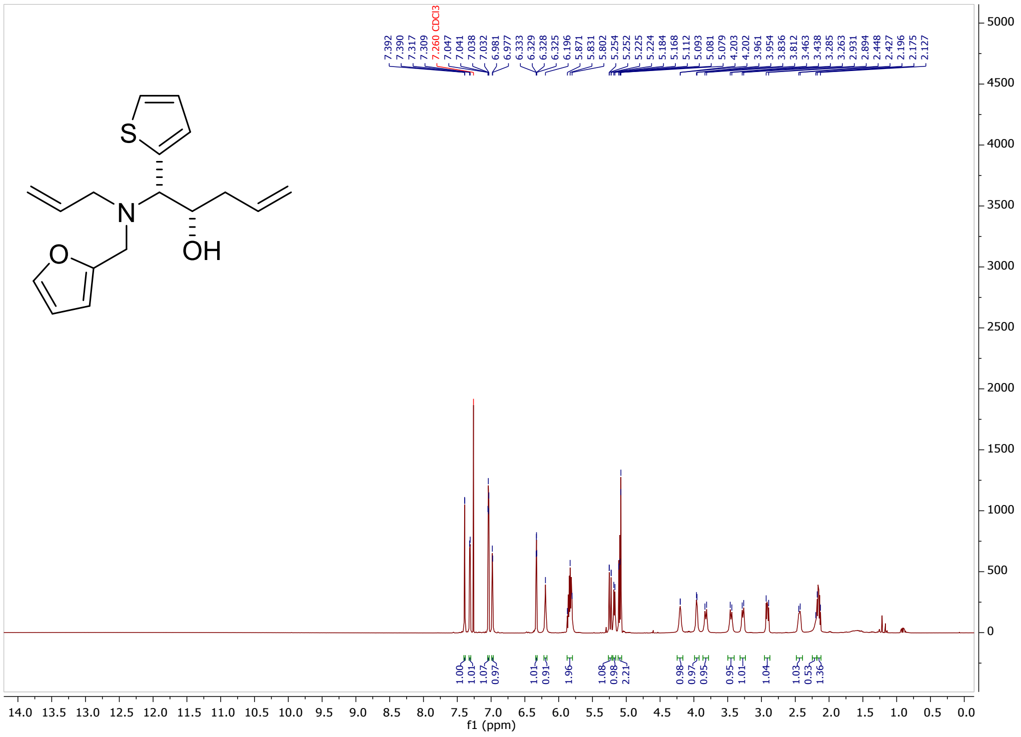

Figure S25. Expanded ^{1}H NMR (600 MHz, $CDCl_3$) of **(4f)**

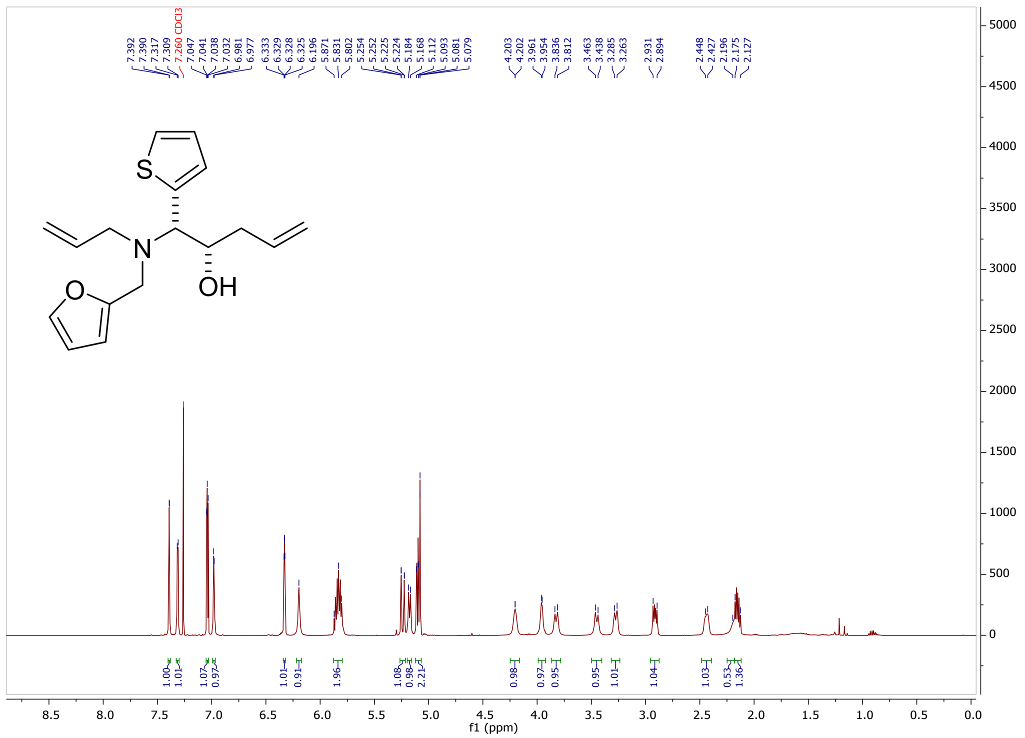

Figure S26. ^{13}C NMR (151 MHz, $CDCl_3$) of **(4f)**

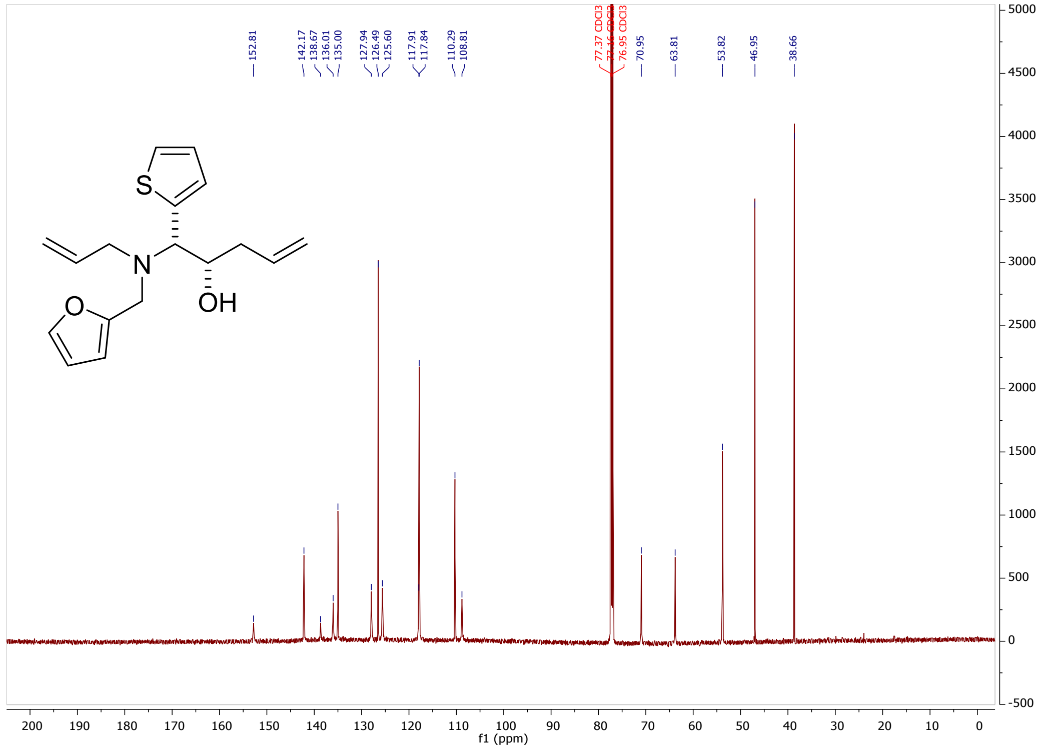

Figure S27. ^{1}H NMR (700 MHz, $CDCl_3$) of (**4g**)

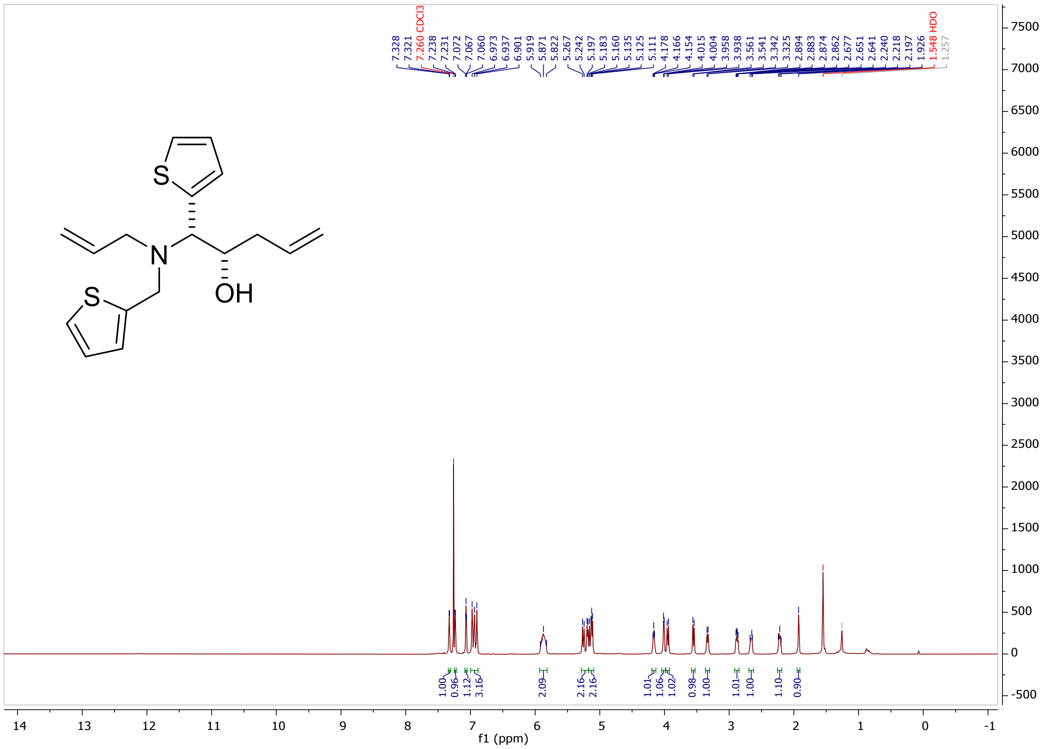

Figure S28. Expanded ^{1}H NMR (700 MHz, $CDCl_3$) of (**4g**)

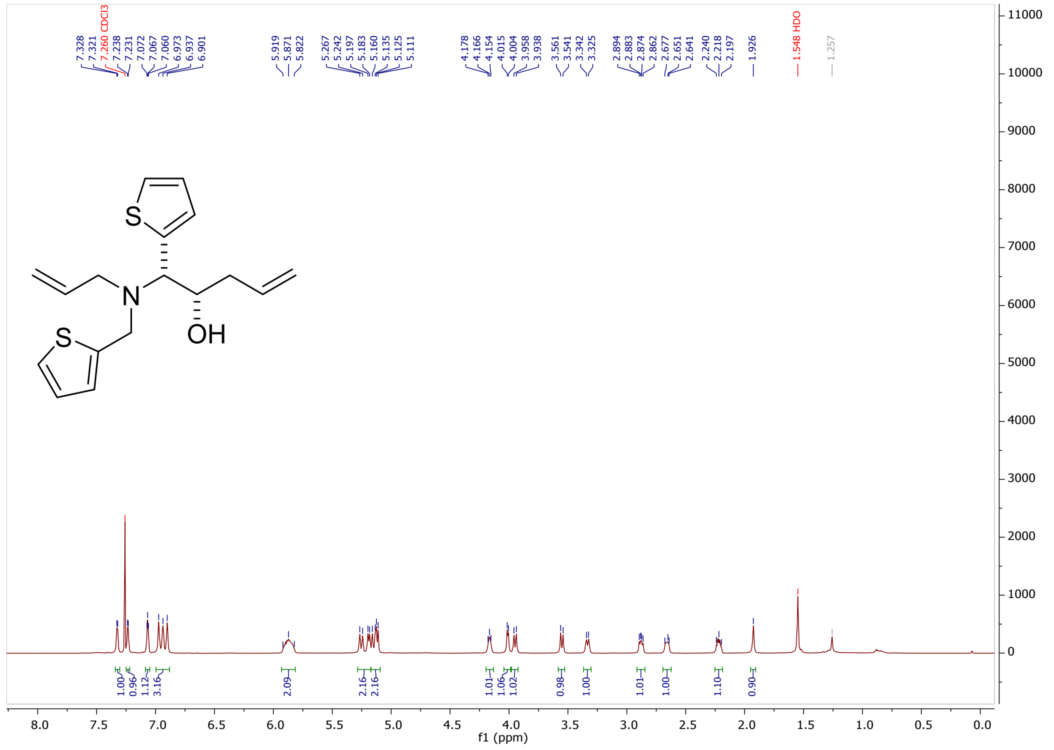

Figure S29. ^{13}C NMR (176 MHz, $CDCl_3$) of (**4g**)

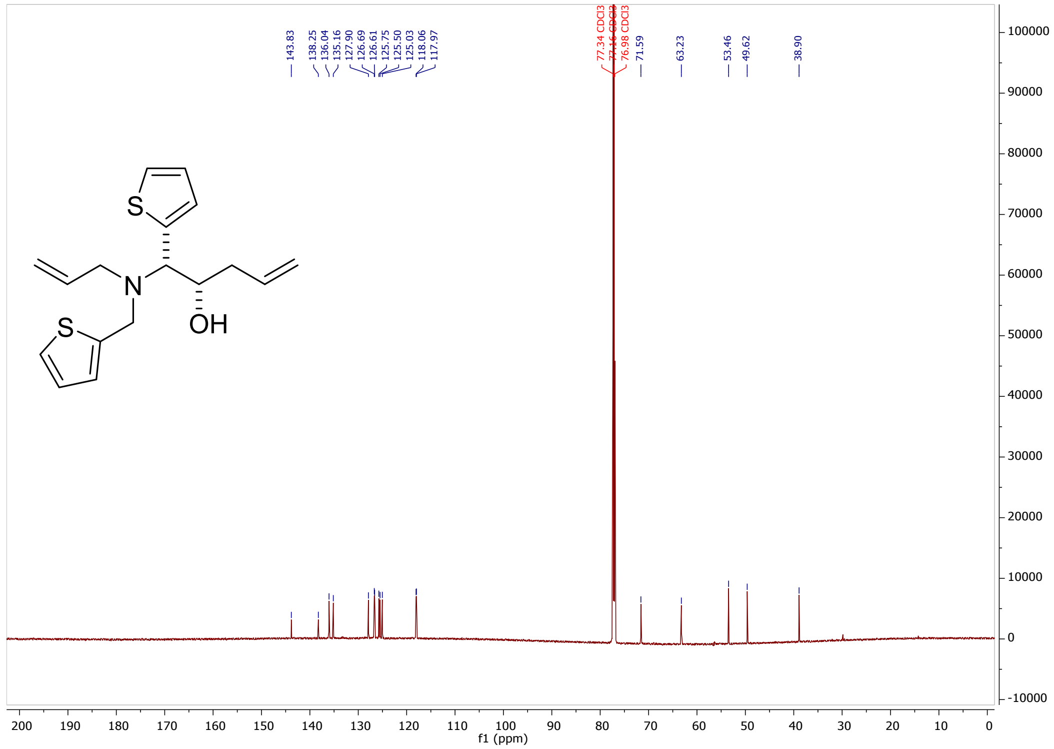

Figure S30. ^{1}H NMR (400 MHz, $CDCl_3$) of **4h**

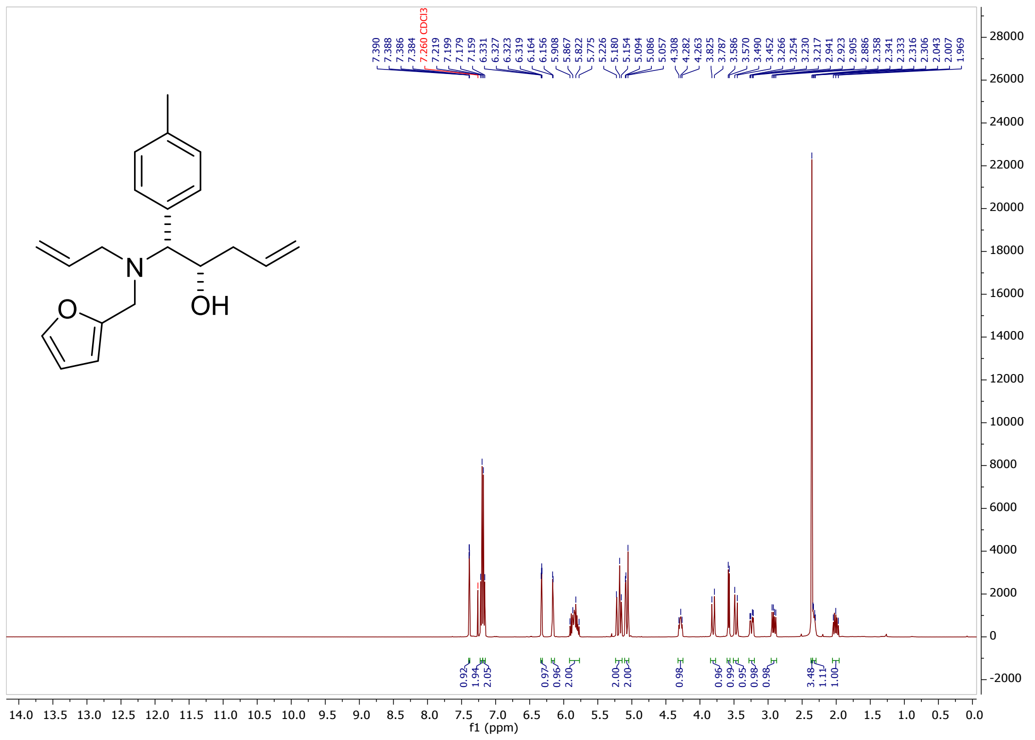

Figure S31. Expanded ^{1}H NMR (400 MHz, $CDCl_3$) of **4h**

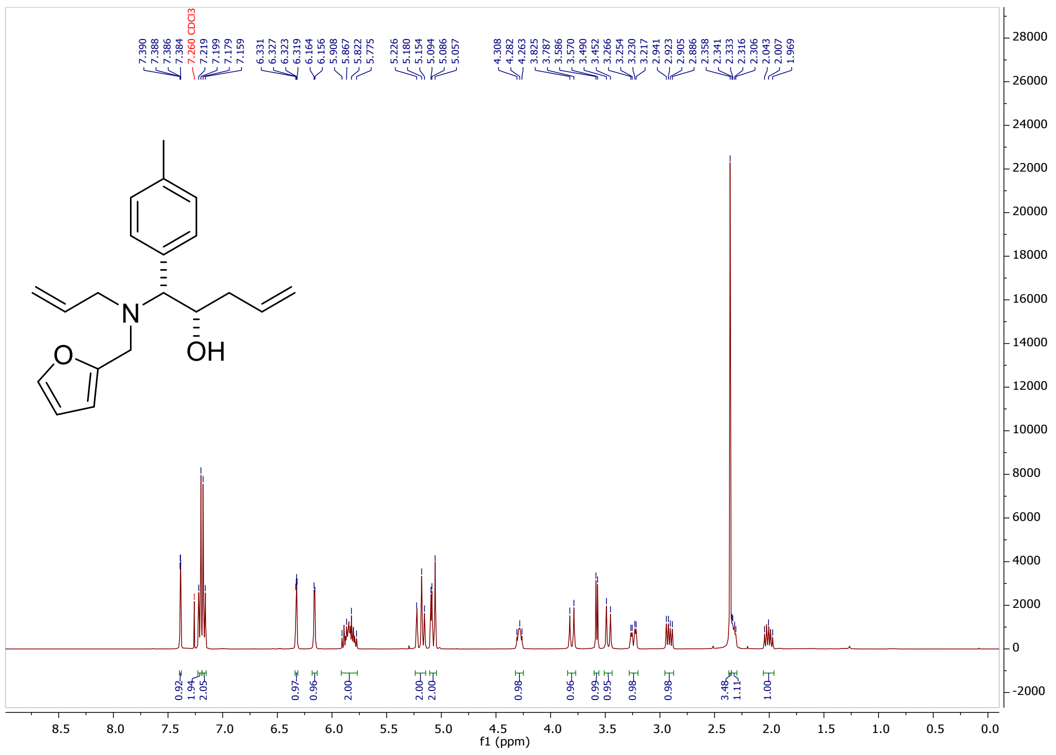

Figure S32. ^{13}C NMR (101 MHz, CDCl3) of **4h**

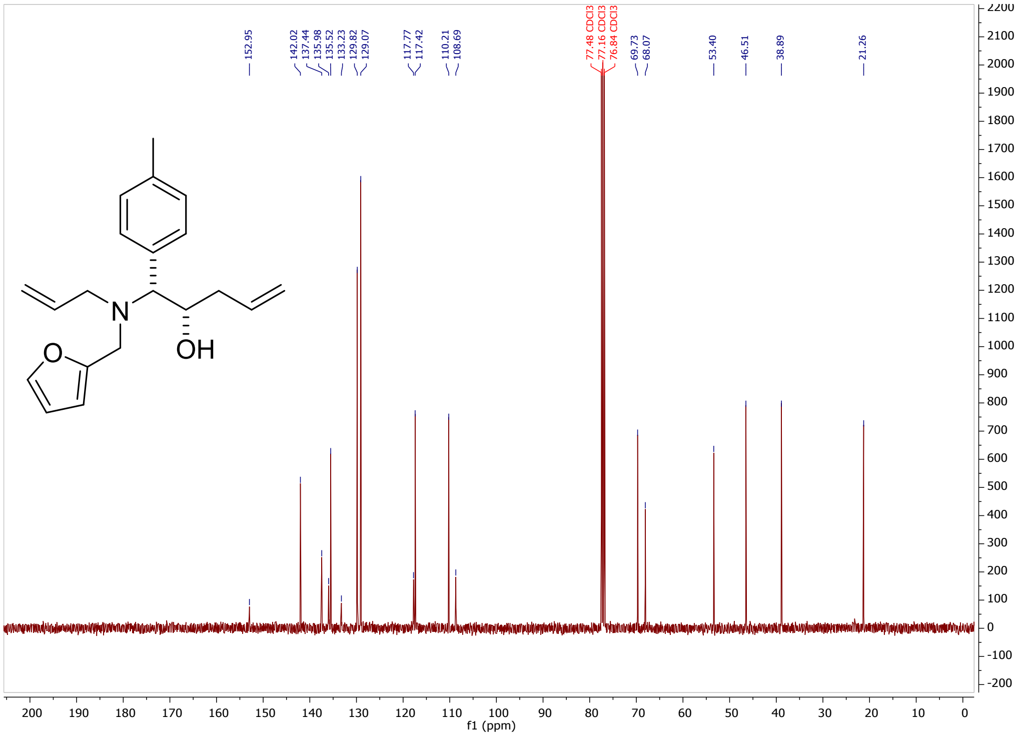

Figure S33. ^{1}H NMR (600 MHz, $CDCl_3$) of **4i**

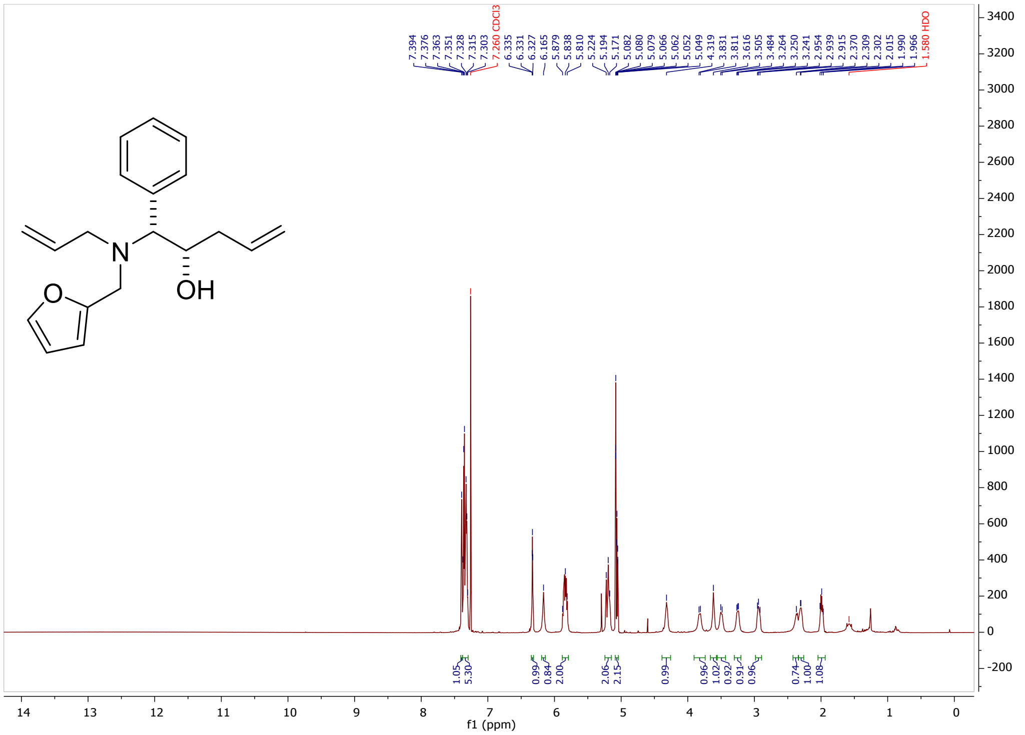

Figure S34. ^{13}C NMR (151 MHz, $CDCl_3$) of **4i**

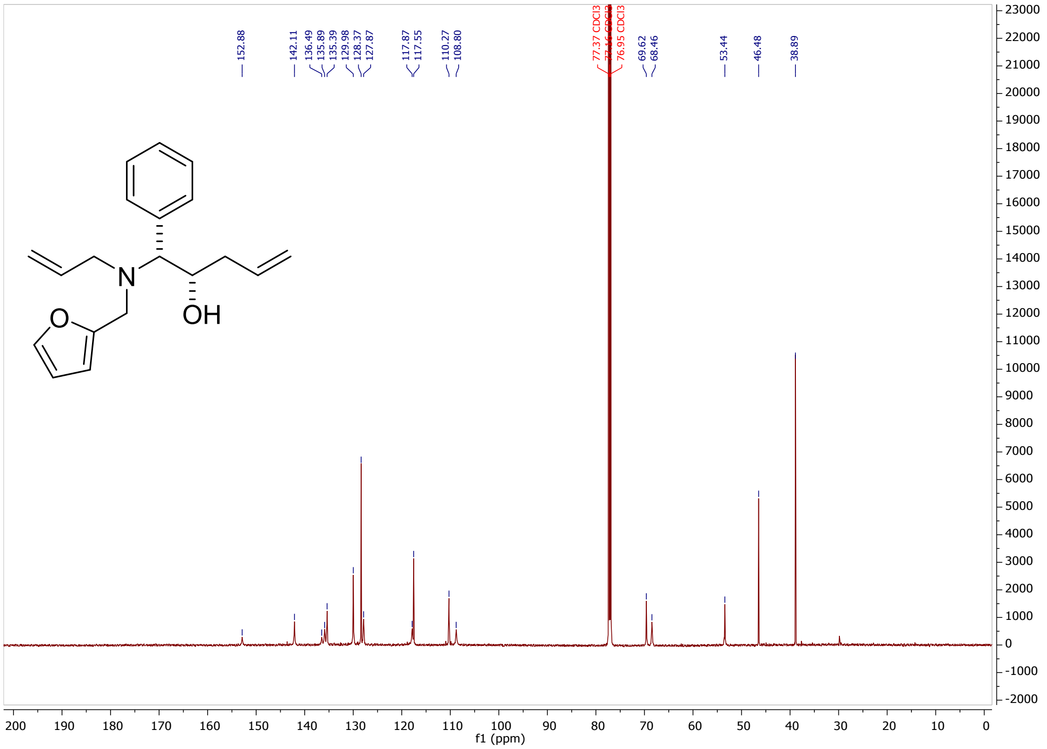

Figure S35. ^{1}H NMR (400 MHz, $CDCl_3$) of **5a**

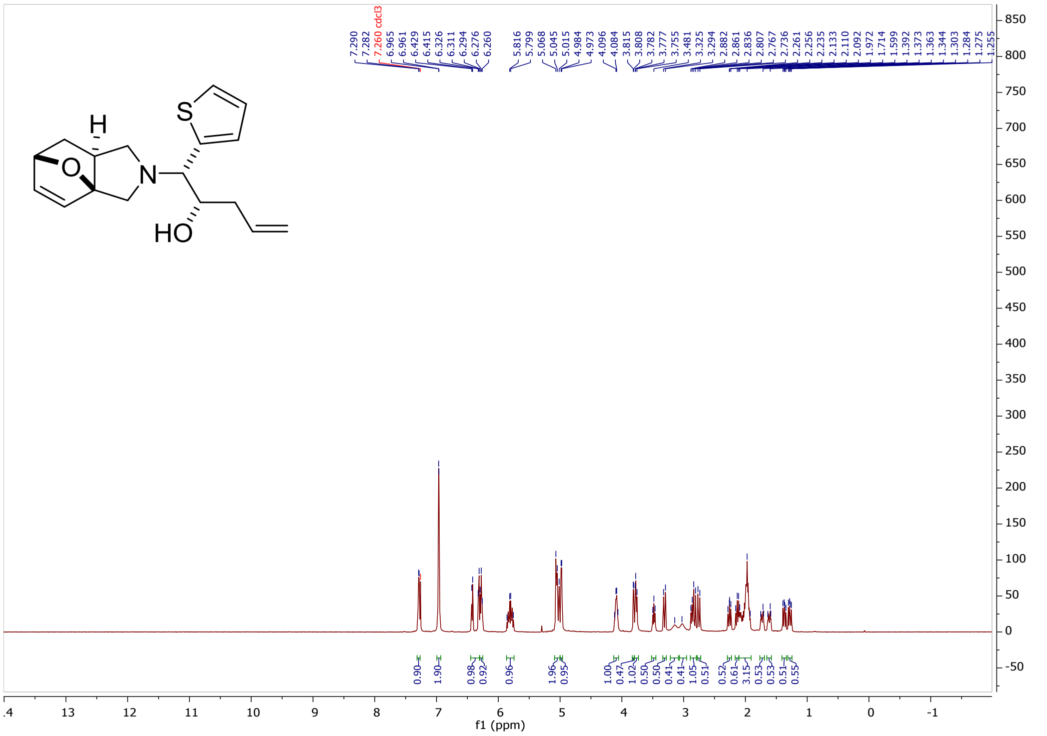

Figure S36. Expanded ^{1}H NMR (400 MHz, $CDCl_3$) of **5a**

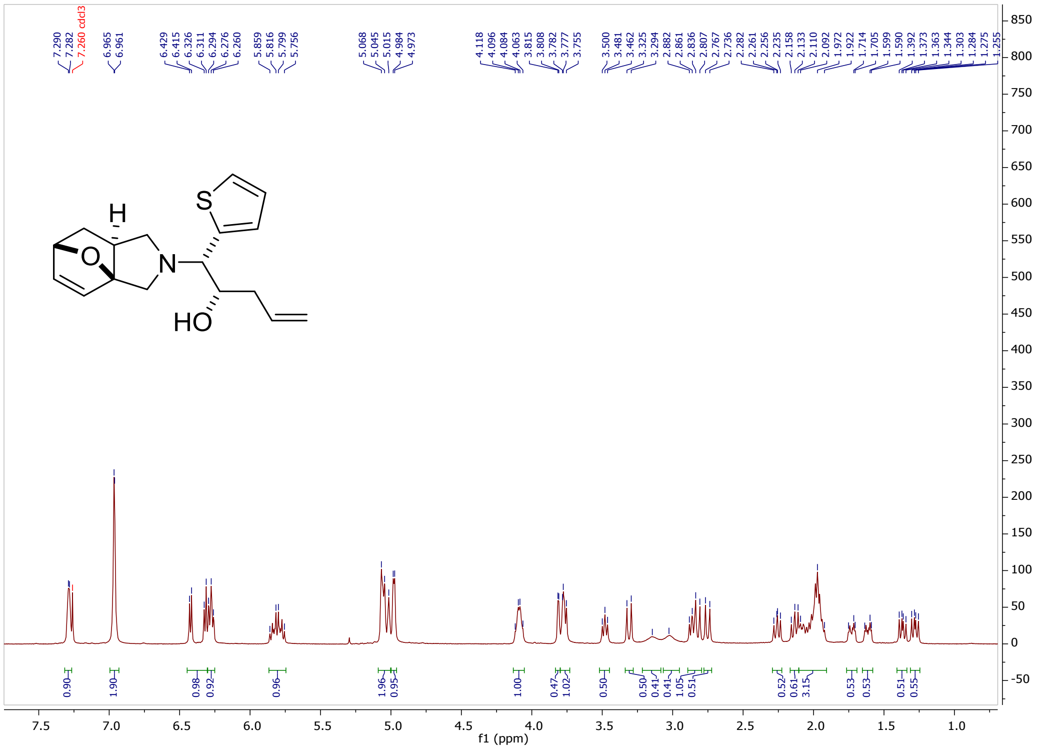

Figure S37. ^{13}C NMR (176 MHz, $CDCl_3$) of **5a**

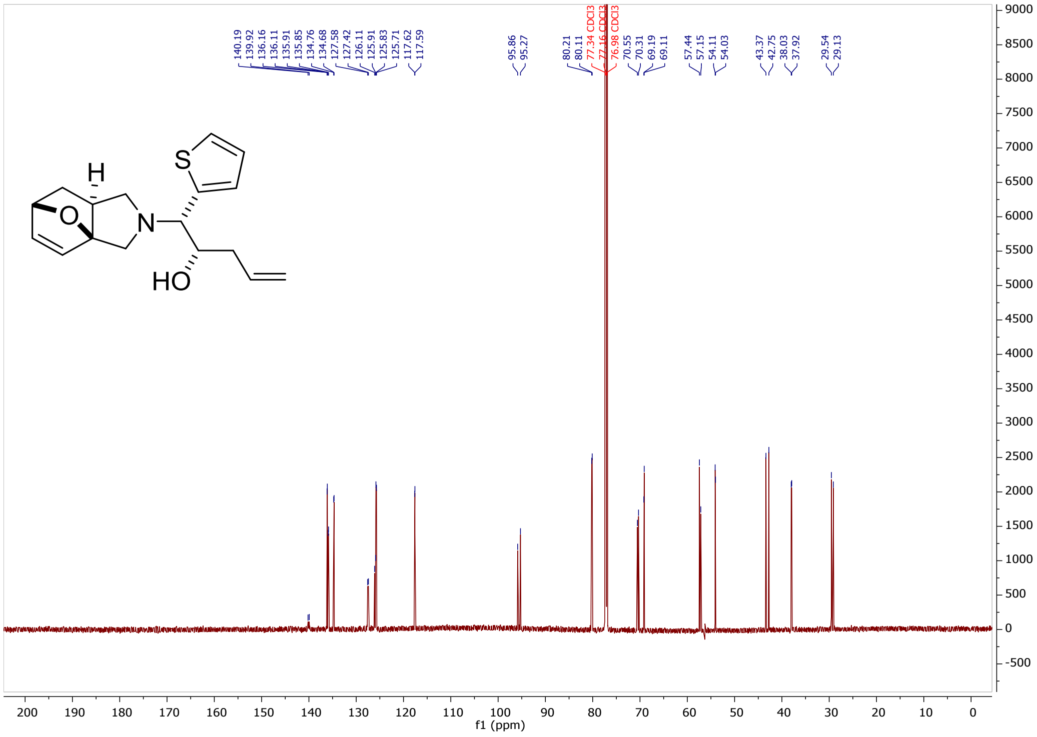

Figure S38. ^{1}H NMR (400 MHz, $CDCl_3$) of **5b**

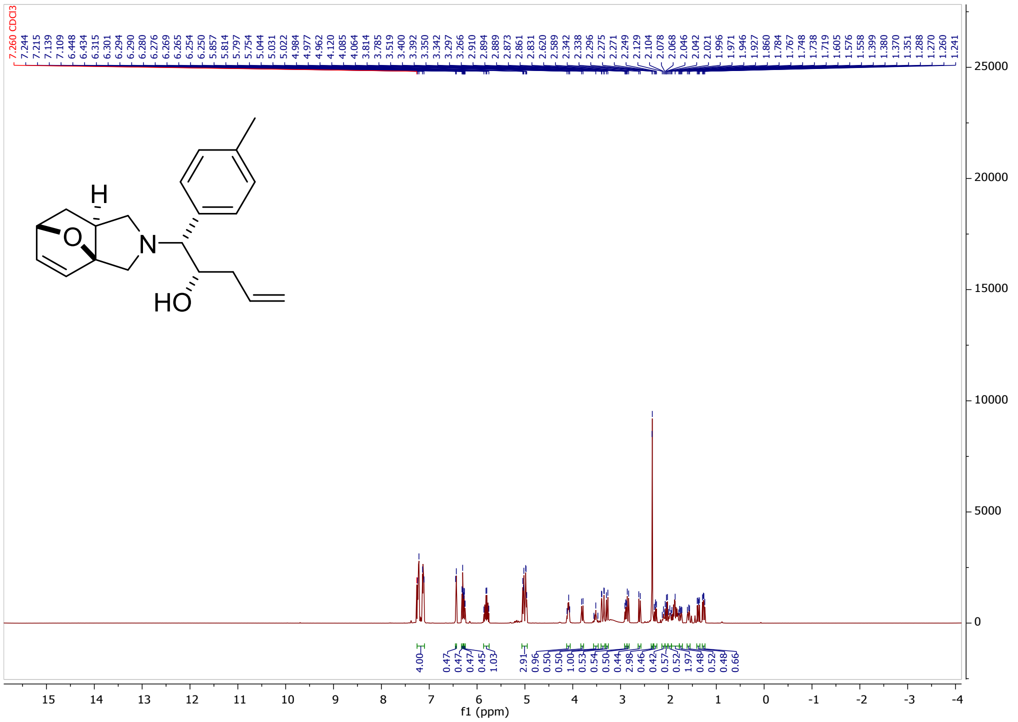

Figure S39. Expanded ^{1}H NMR (400 MHz, $CDCl_3$) of **5b**

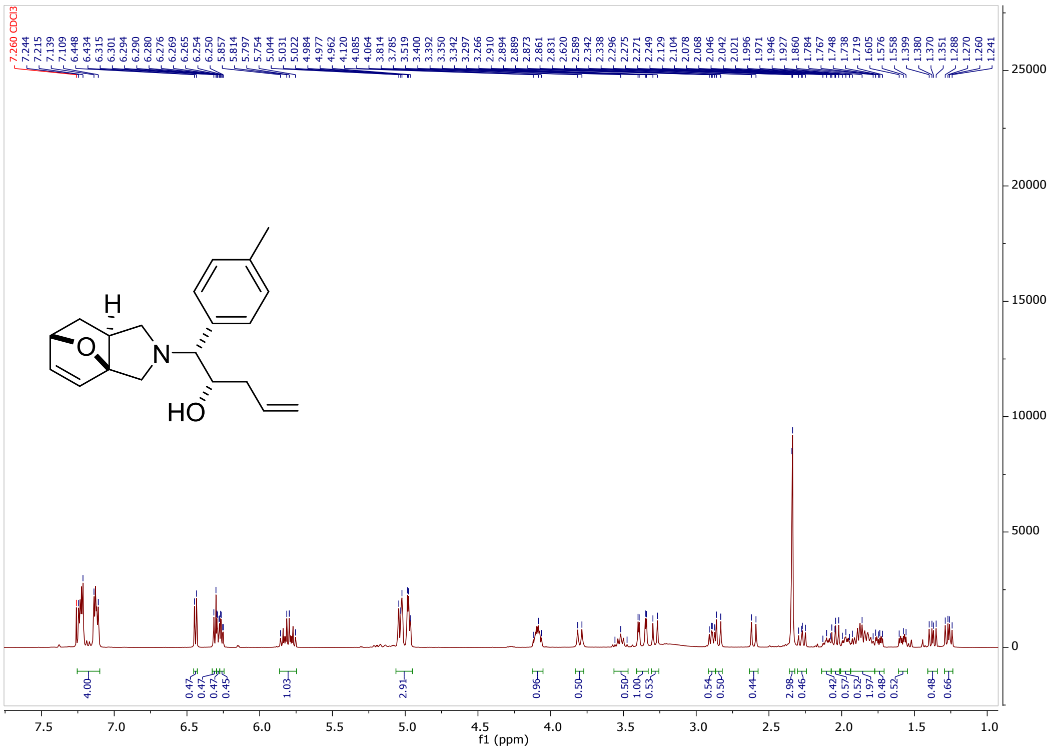

Figure S40. ^{13}C NMR (101 MHz, $CDCl_3$) of **5b**

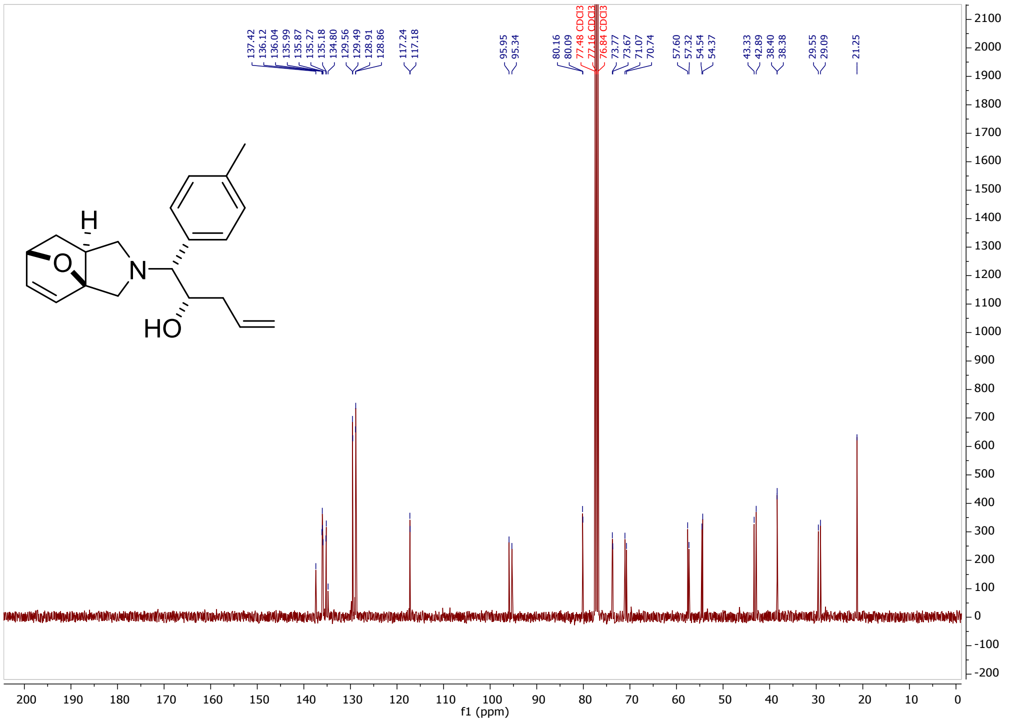

Figure S41. ^{1}H NMR (400 MHz, $CDCl_3$) of **5c**

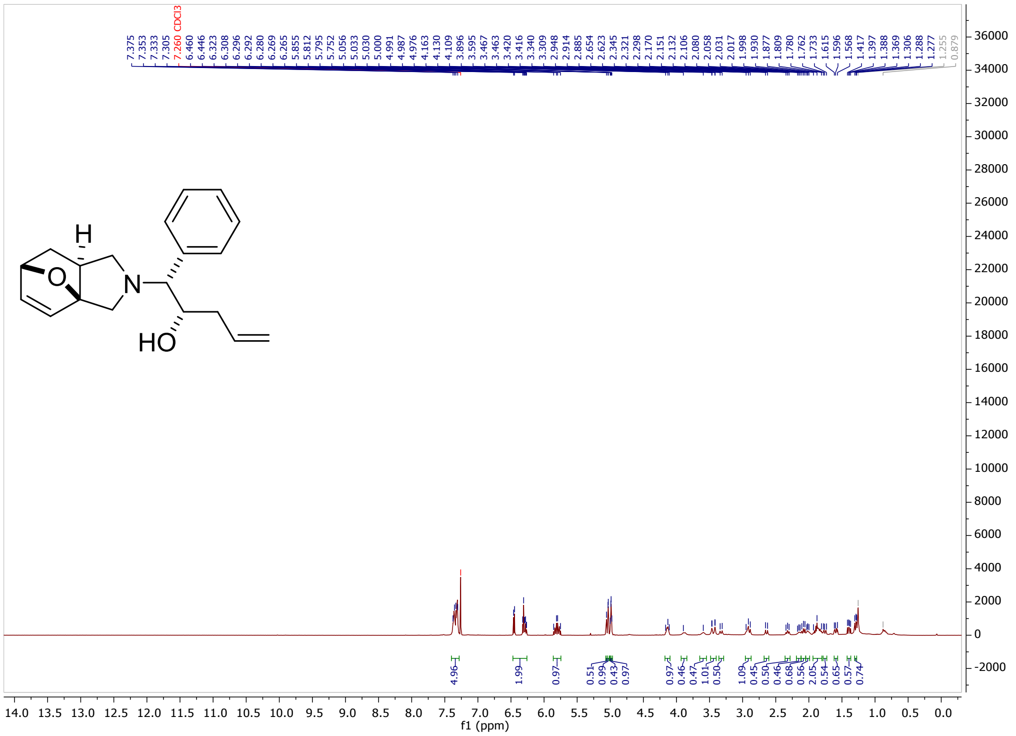

Figure S42. Expanded ^{1}H NMR (400 MHz, $CDCl_3$) of **5c**

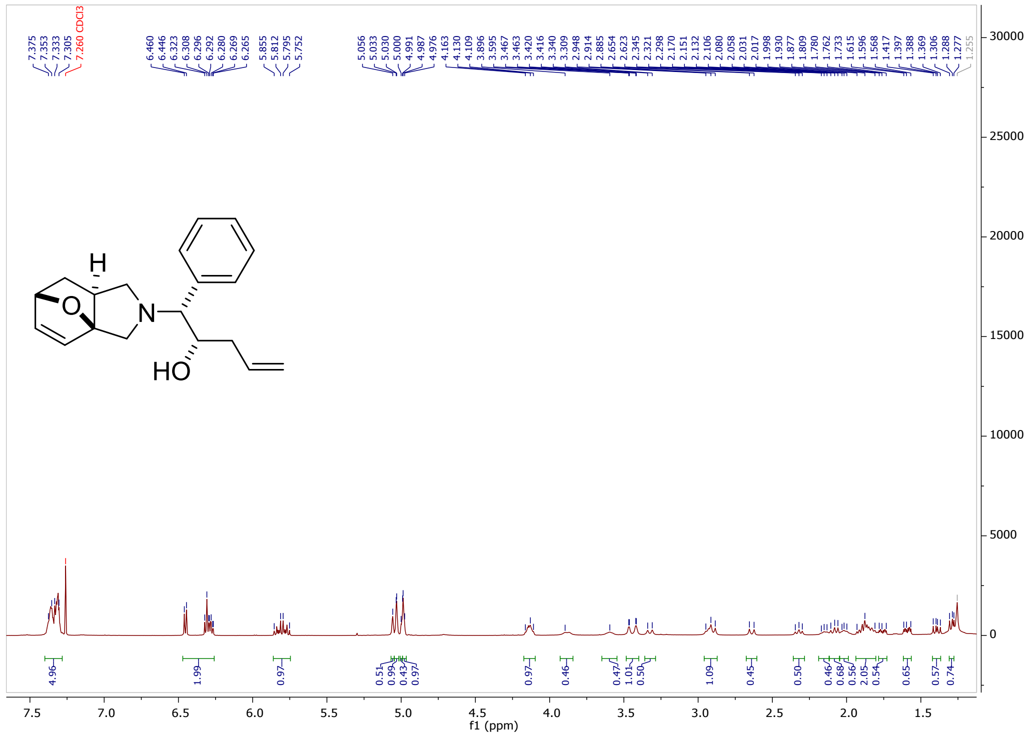

Figure S43. ^{13}C NMR (101 MHz, $CDCl_3$) of **5c**

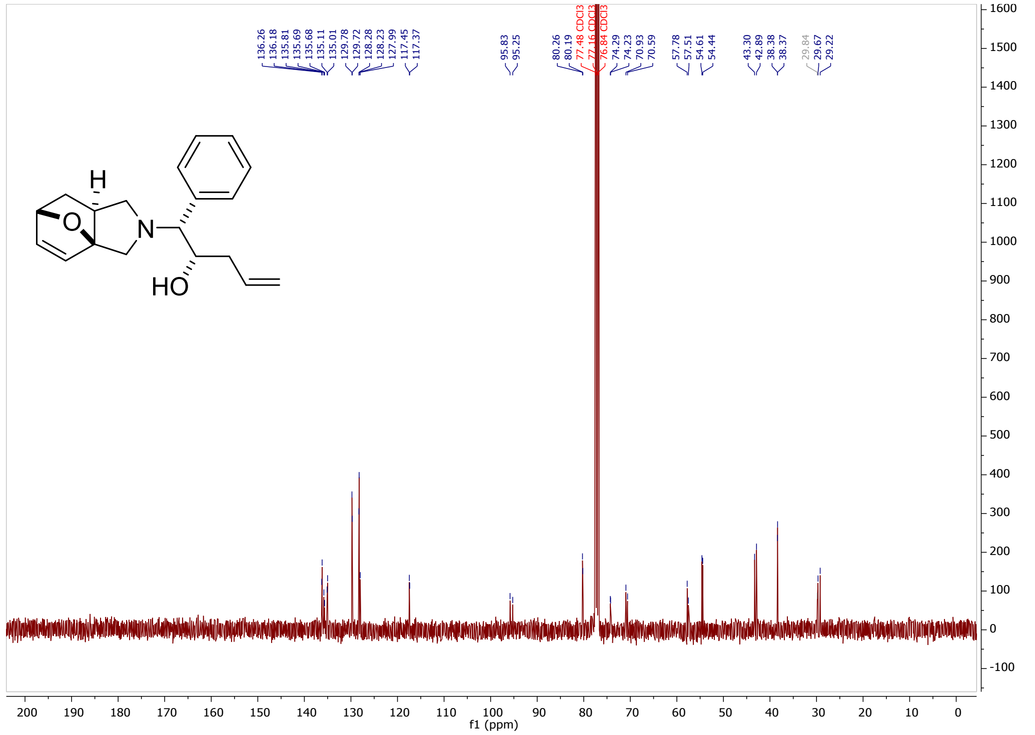

Figure S44. ^{1}H NMR (500 MHz, $CDCl_3$) of **7a**

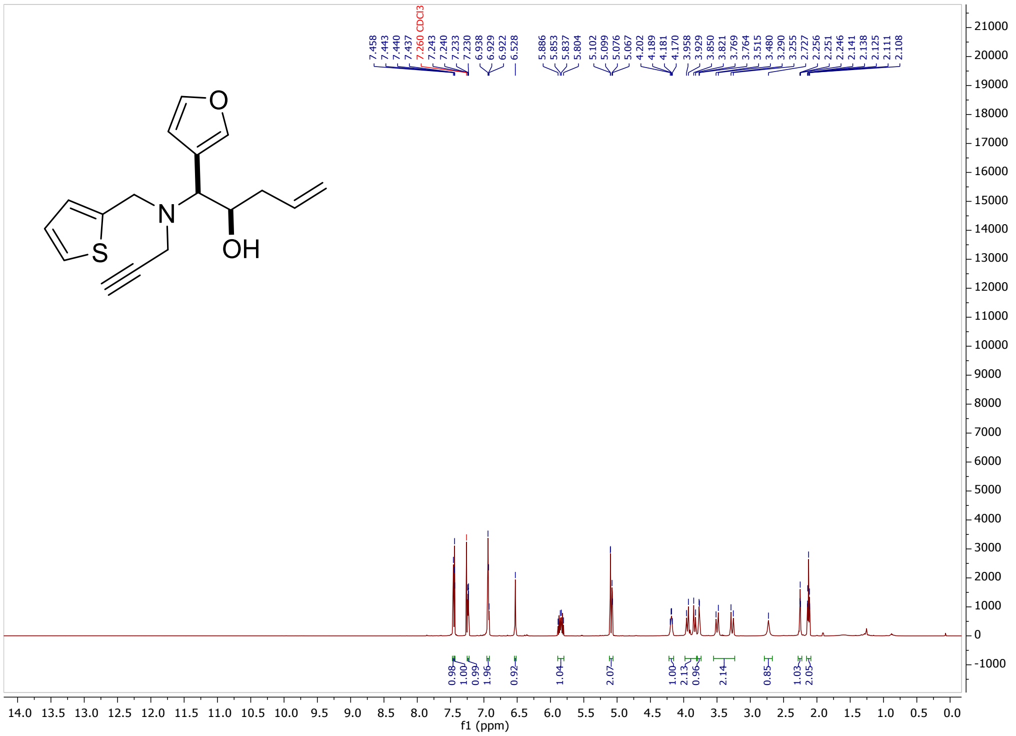

Figure S45. Expanded ^{1}H NMR (500 MHz, $CDCl_3$) of **7a**

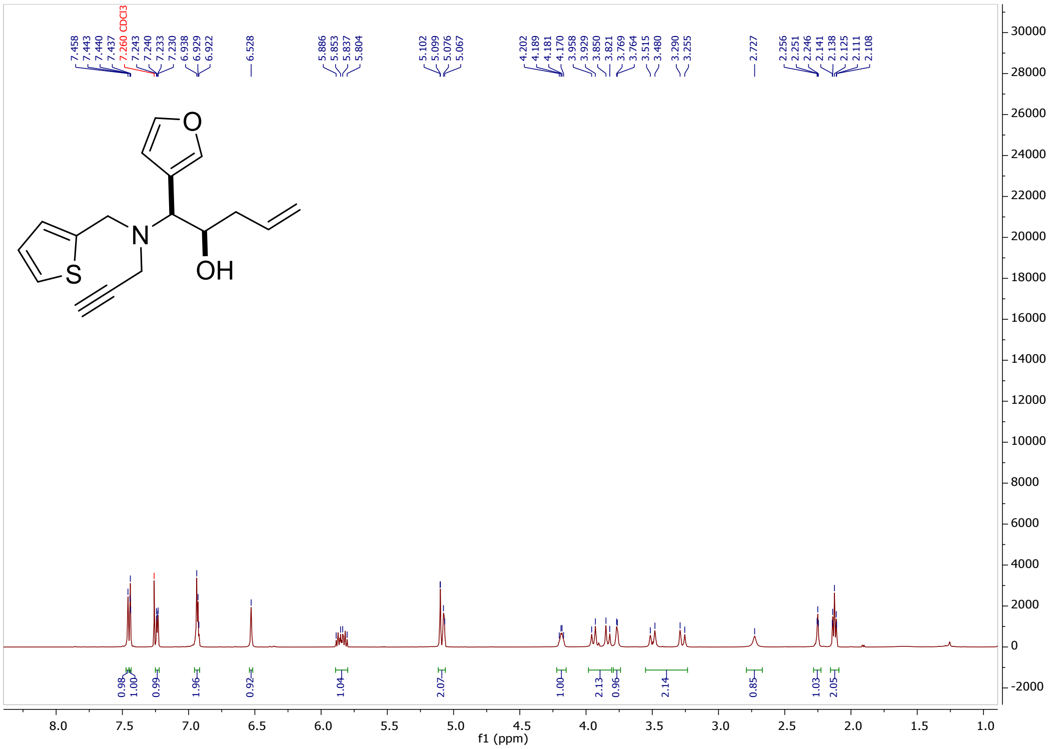

Figure S46. ^{13}C NMR (126 MHz, $CDCl_3$) of **7a**

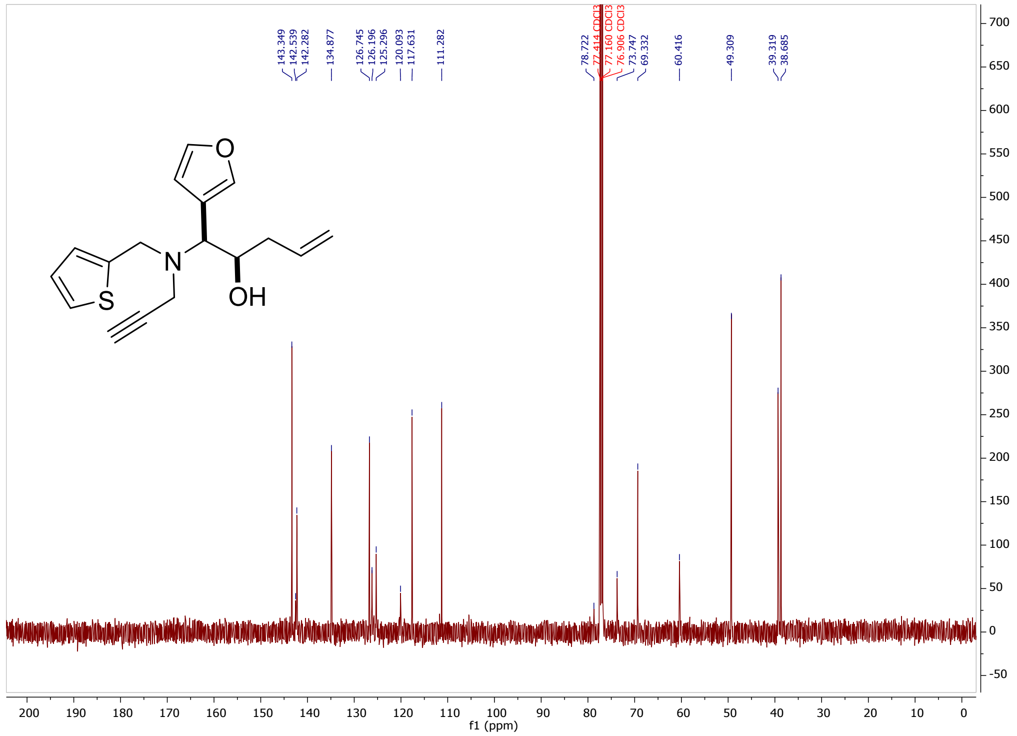

Figure S47. ^{1}H NMR (600 MHz, $CDCl_3$) of **7b**

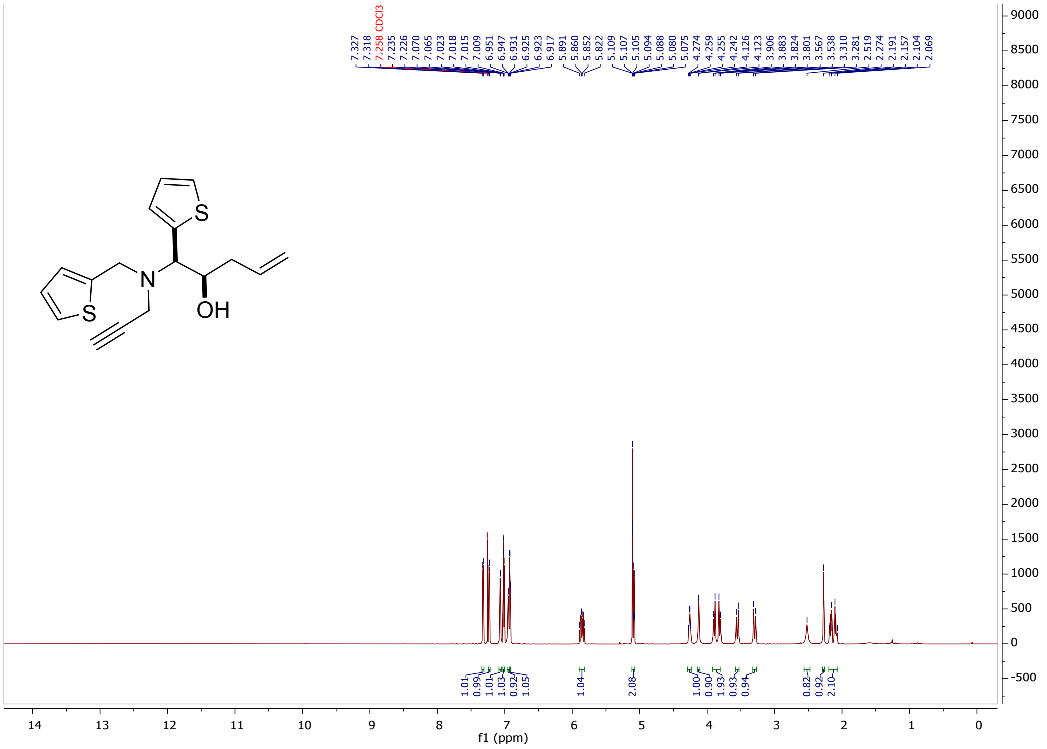

Figure S48. Expanded ^{1}H NMR (600 MHz, $CDCl_3$) of **7b**

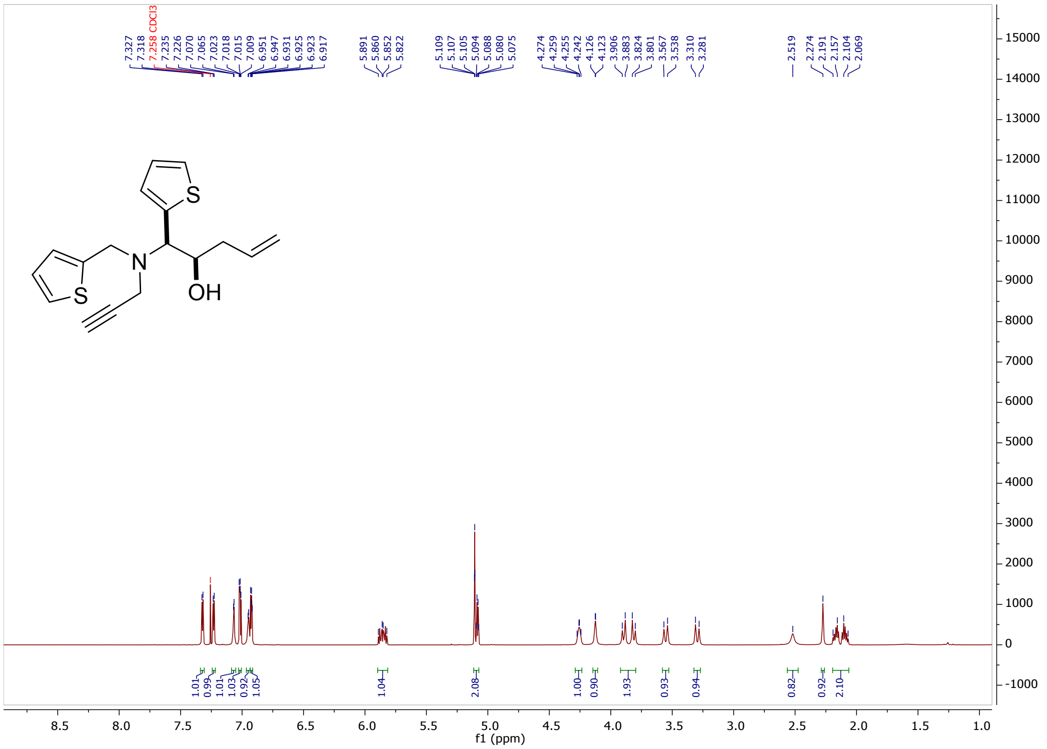

Figure S49. ^{13}C NMR (151 MHz, $CDCl_3$) of **7b**

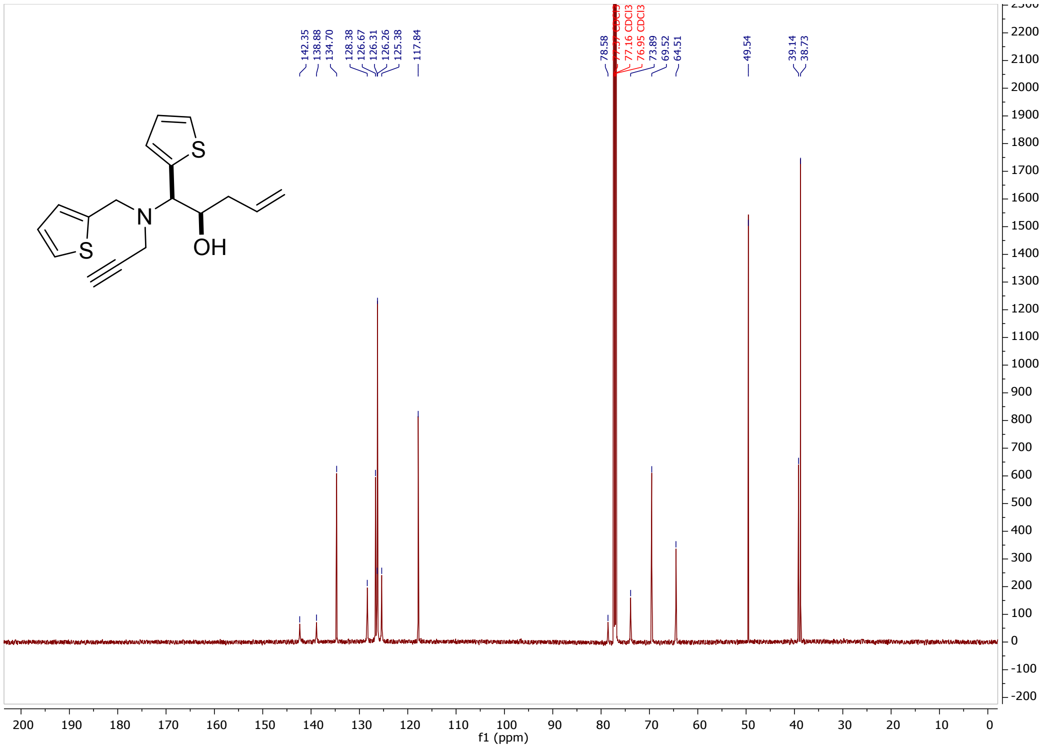

Figure S50. ^{1}H NMR (700 MHz, $CDCl_3$) of **7c**

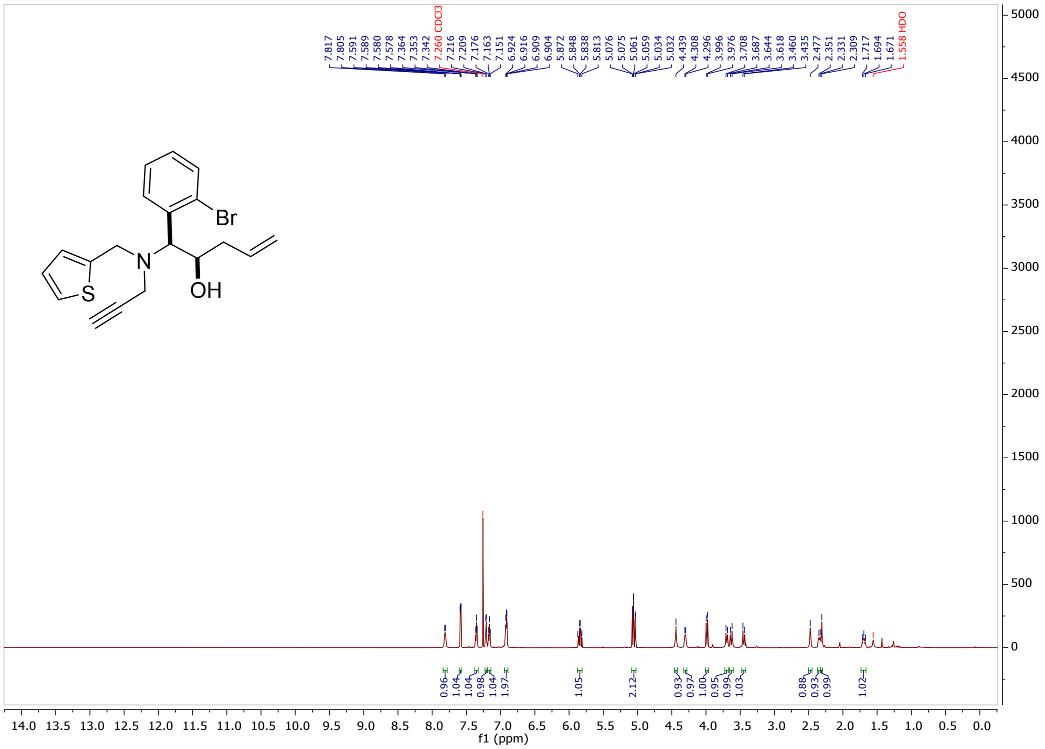

Figure S51. Expanded ^{1}H NMR (700 MHz, $CDCl_3$) of **7c**

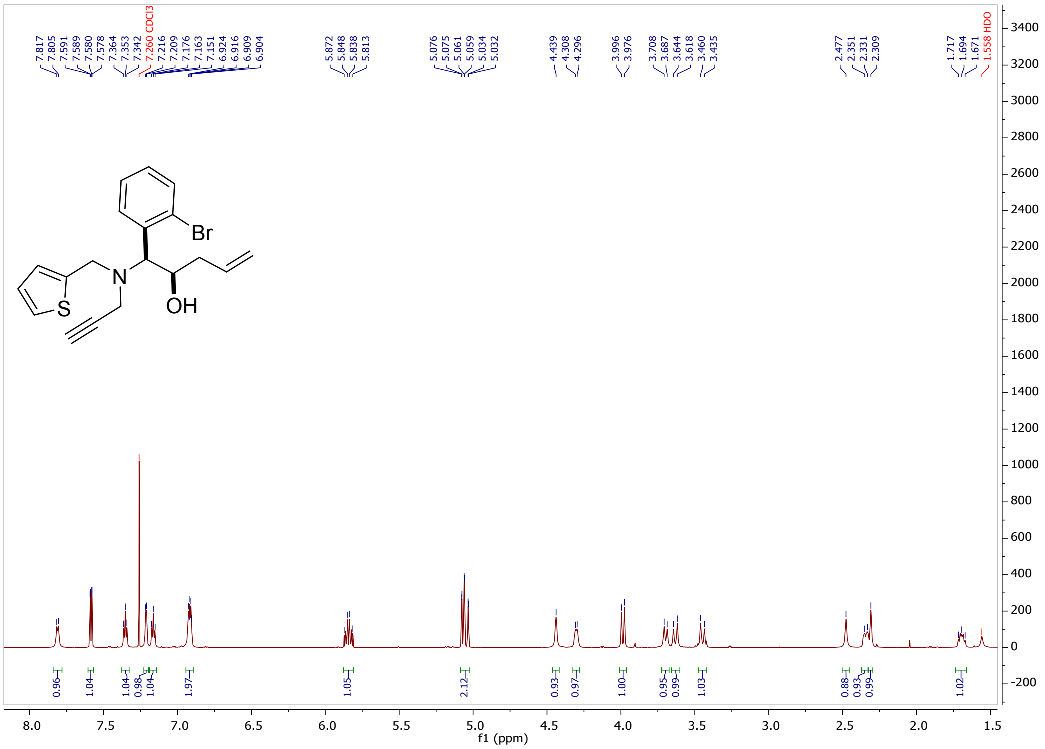

Figure S52. ^{13}C NMR (176 MHz, $CDCl_3$) of **7c**

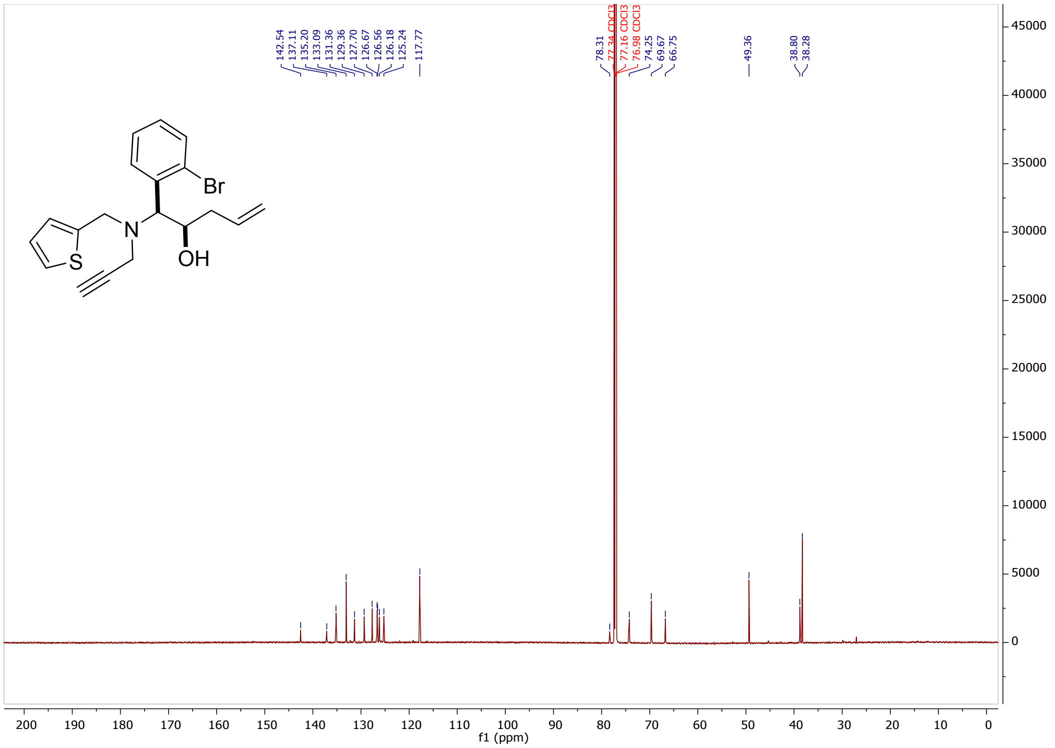

Figure S53. ^{1}H NMR (400 MHz, $CDCl_3$) **7d**

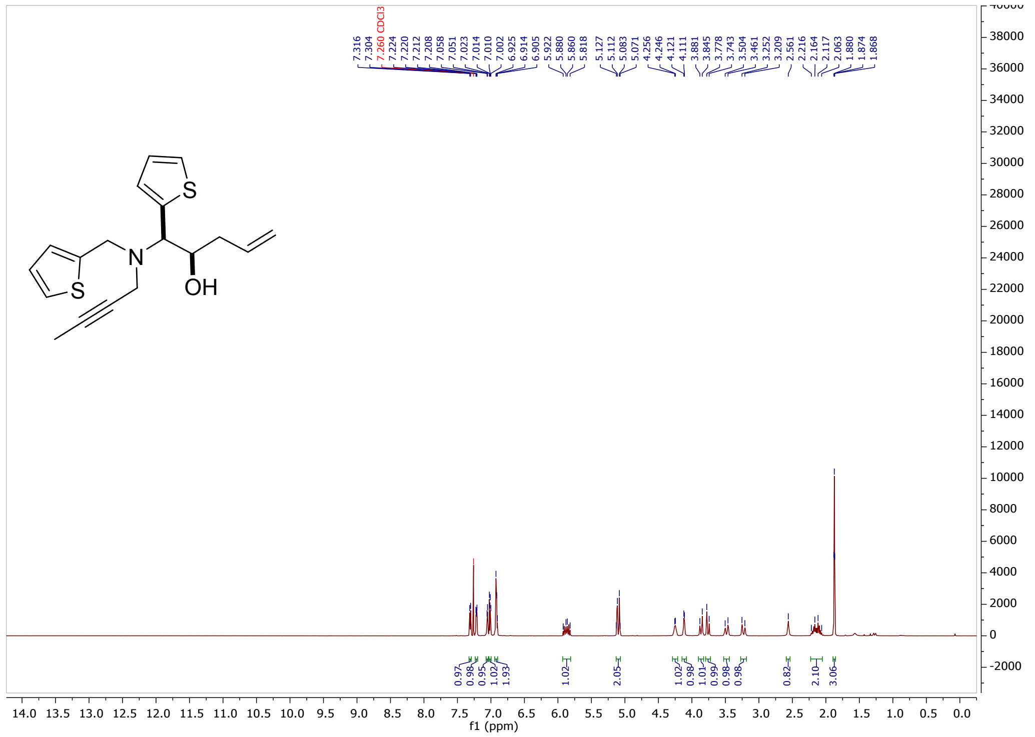

Figure S54. Expanded ^{1}H NMR (400 MHz, $CDCl_3$) **7d**

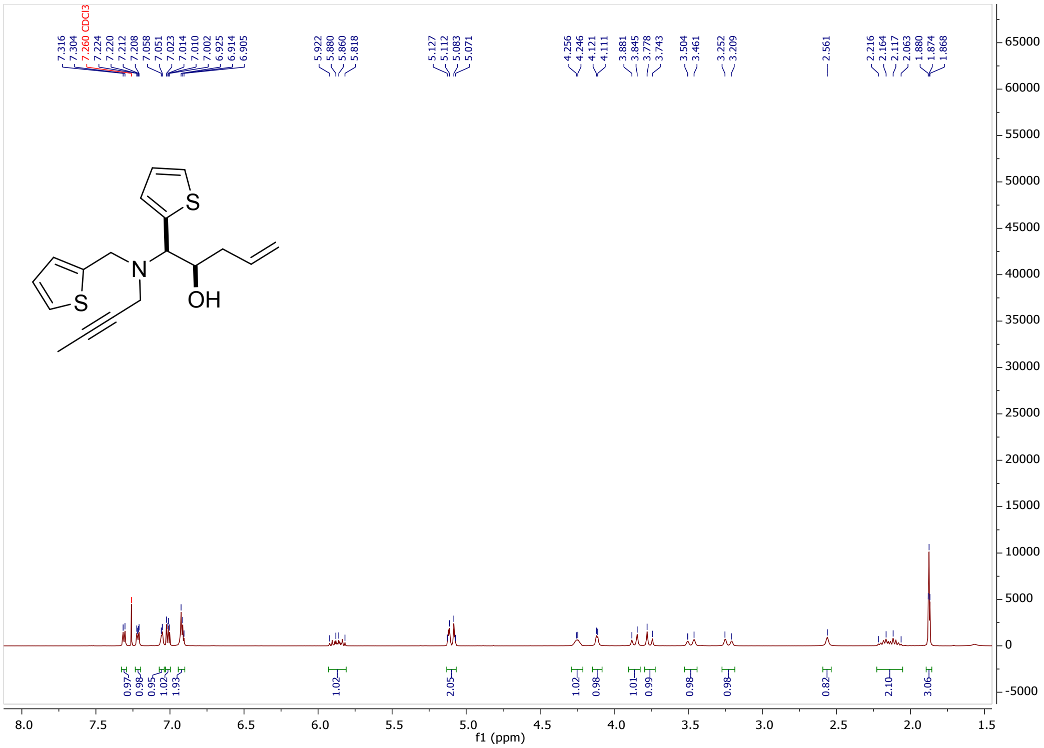

Figure S55. ^{13}C NMR (101 MHz, CDCl$_3$) **7d**

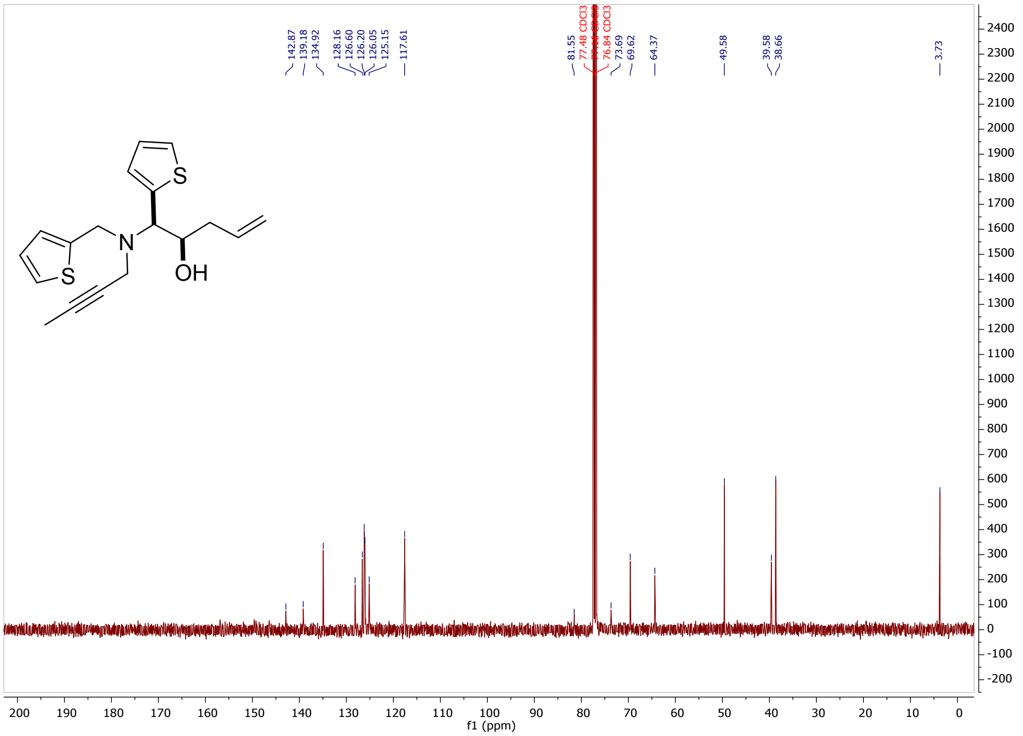

Figure S56. ^{1}H NMR (500 MHz, CDCl$_3$) **8a**

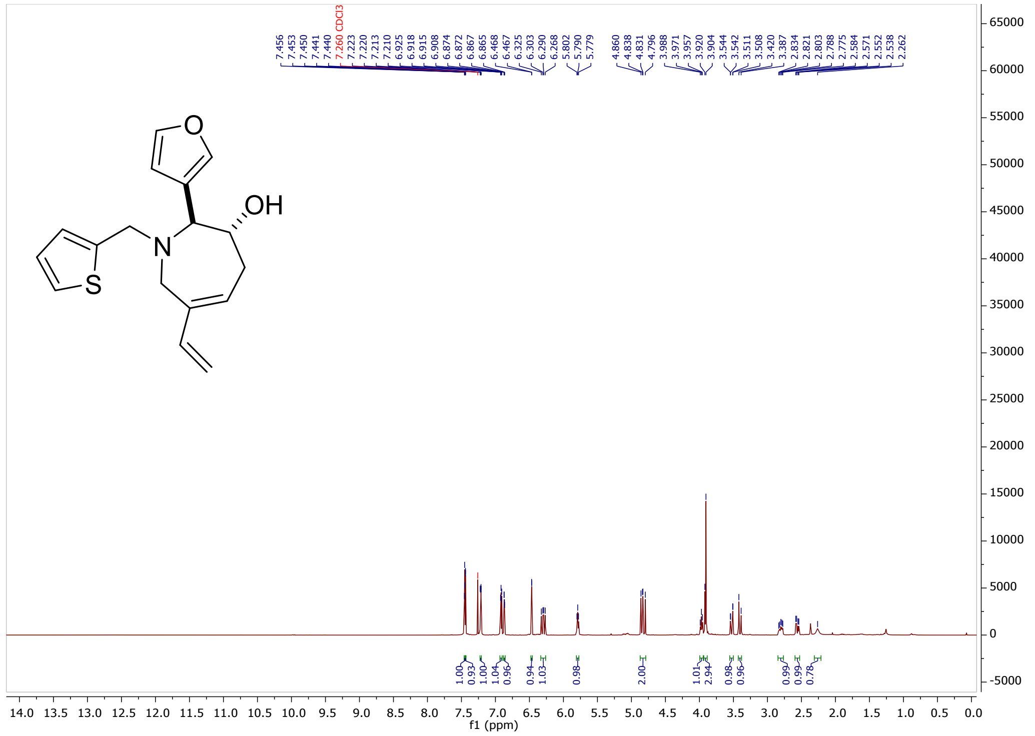

Figure S57. Expanded ^{1}H NMR (500 MHz, $CDCl_3$) **8a**

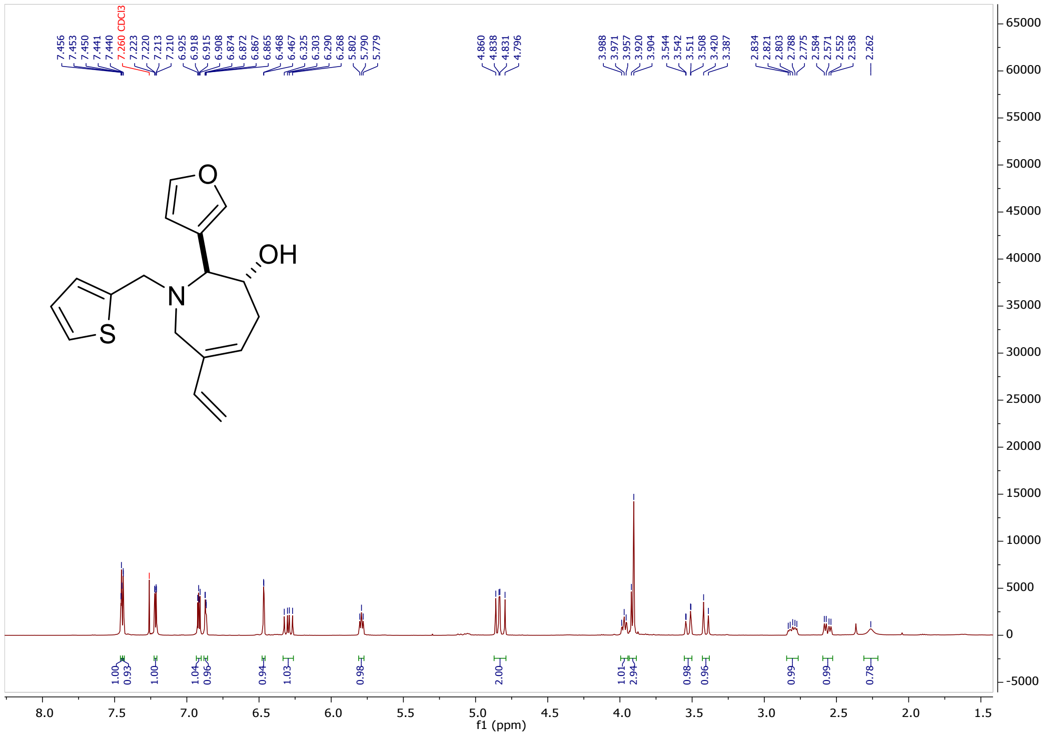

Figure S58. ^{13}C NMR (126 MHz, $CDCl_3$) **8a**

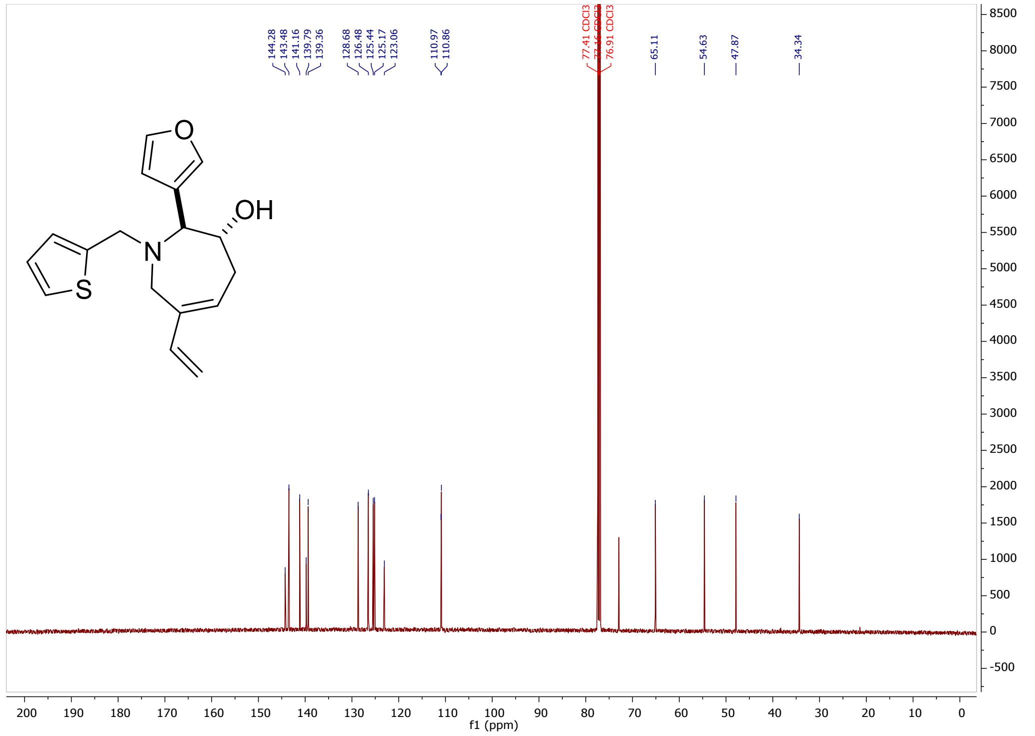

Figure S59. ^{1}H NMR (500 MHz, $CDCl_3$) of **8b**

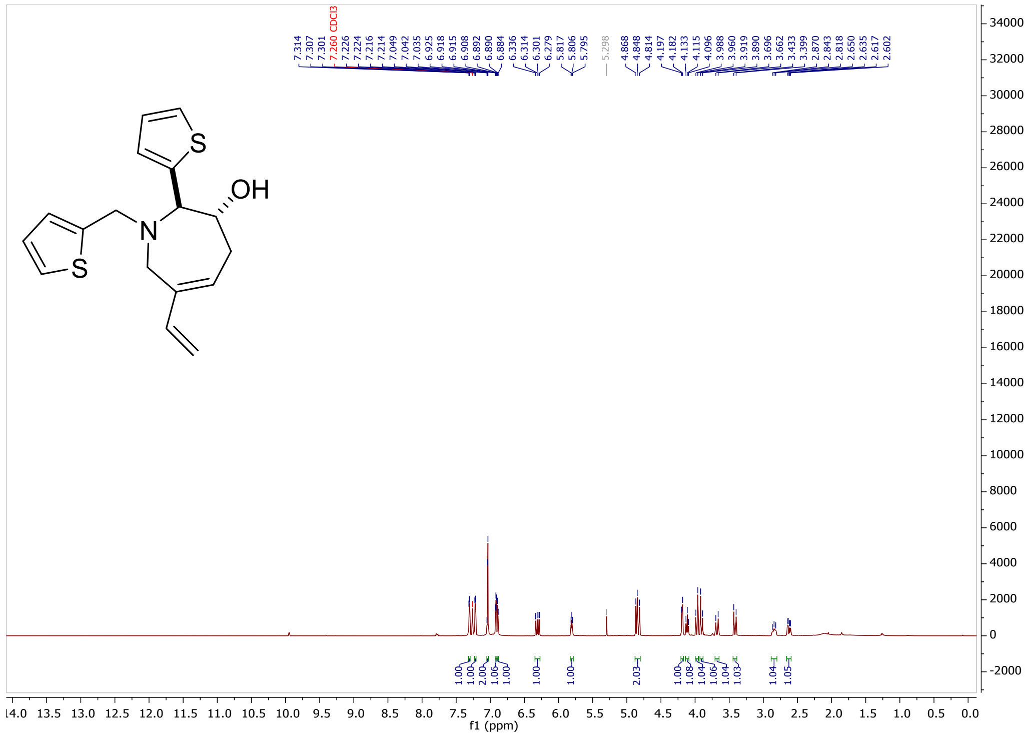

Figure S60. Expanded ^{1}H NMR (500 MHz, $CDCl_3$) of **8b**

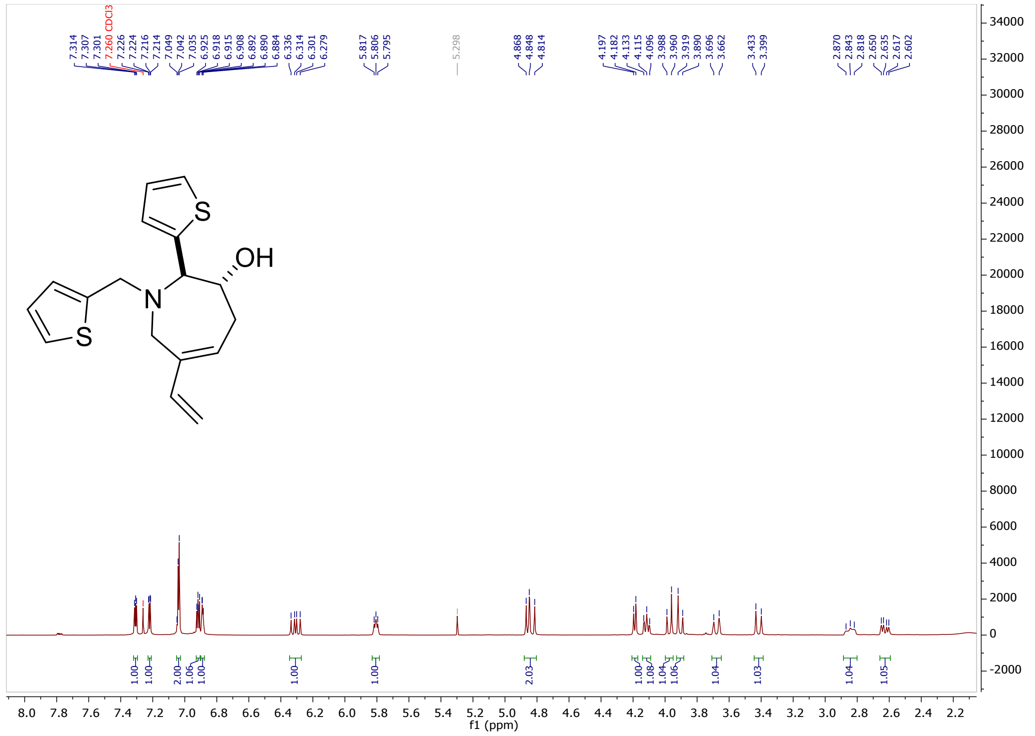

Figure S61. ^{13}C NMR (126 MHz, $CDCl_3$) of **8b**

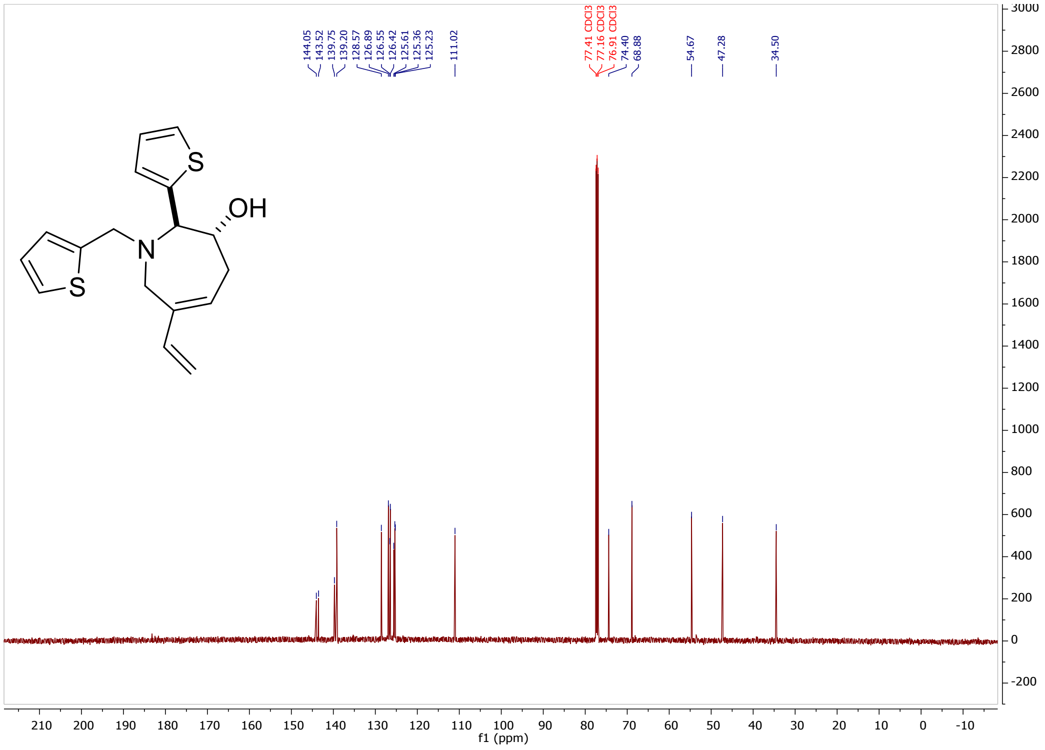

Figure S62. ^{1}H NMR (500 MHz, $CDCl_3$) of **8c**

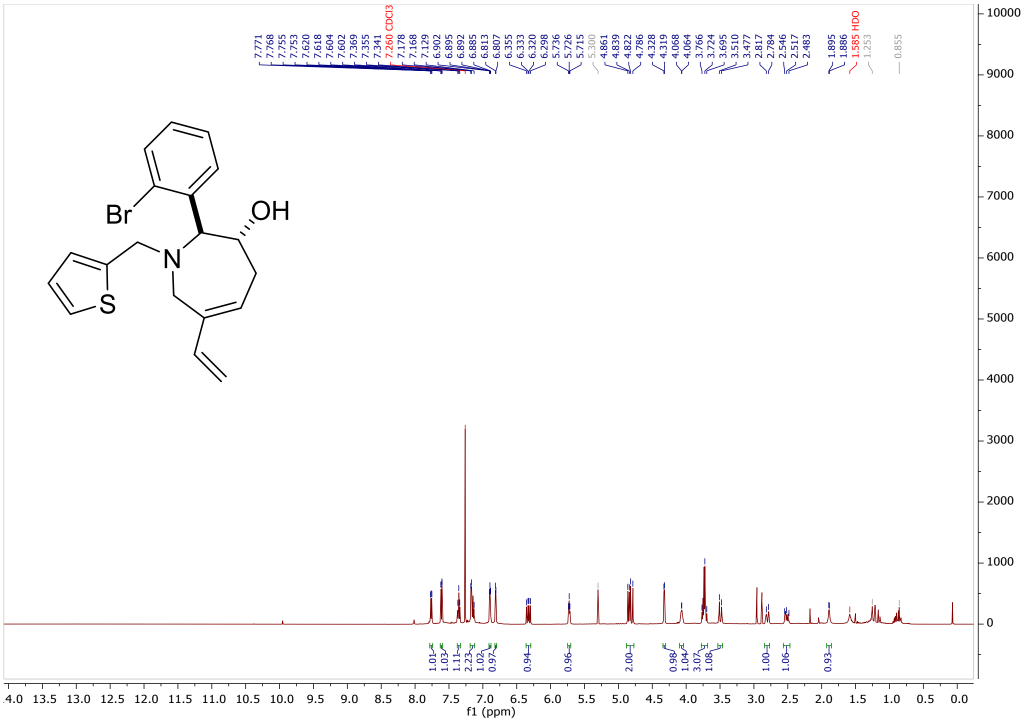

Figure S63. Expanded ^{1}H NMR (500 MHz, $CDCl_3$) of **8c**

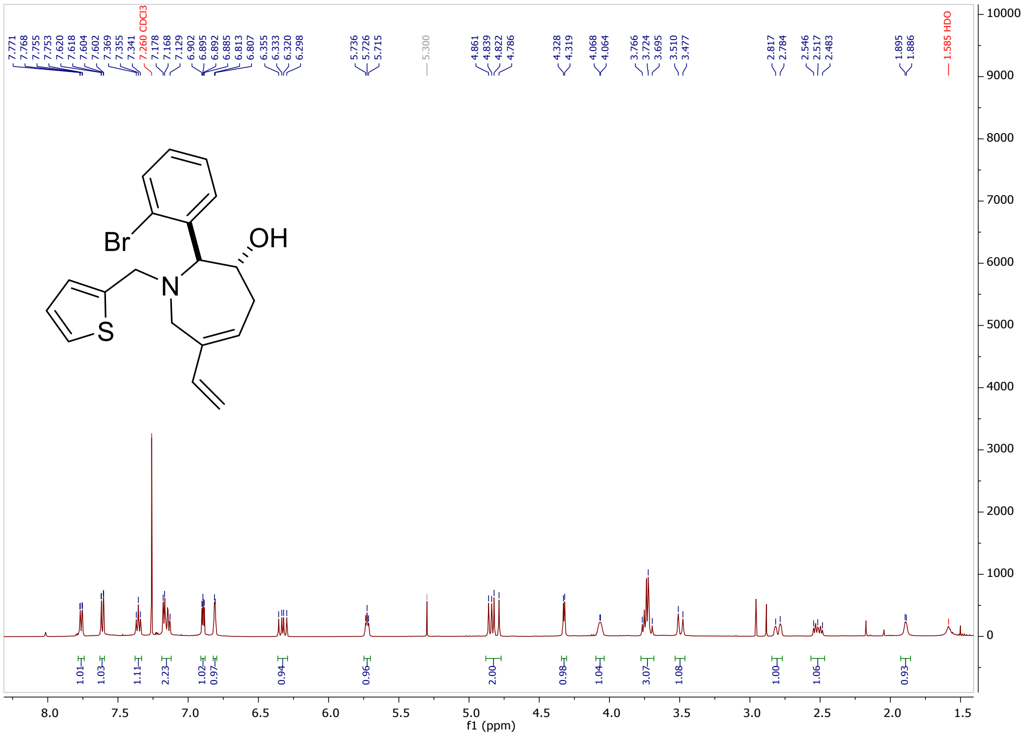

Figure S64. ^{13}C NMR (126 MHz, $CDCl_3$) of **8c**

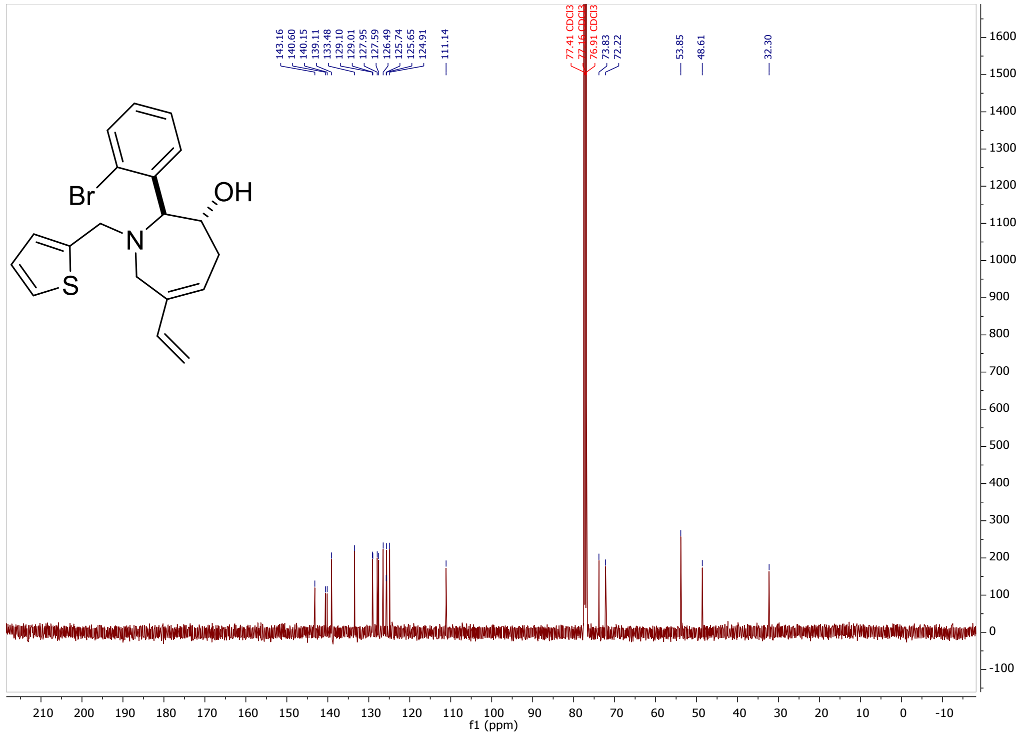

Figure S65. ^{1}H NMR (500 MHz, $CDCl_3$) of **8d**

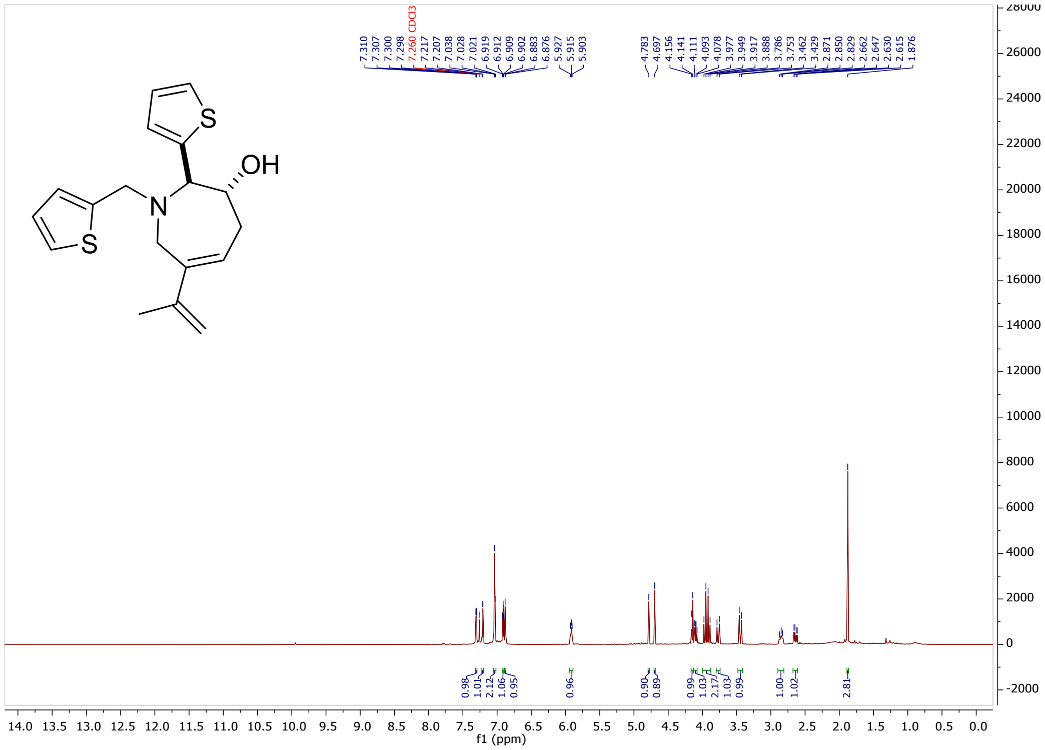

Figure S66. Expanded ^{1}H NMR (500 MHz, $CDCl_3$) of **8d**

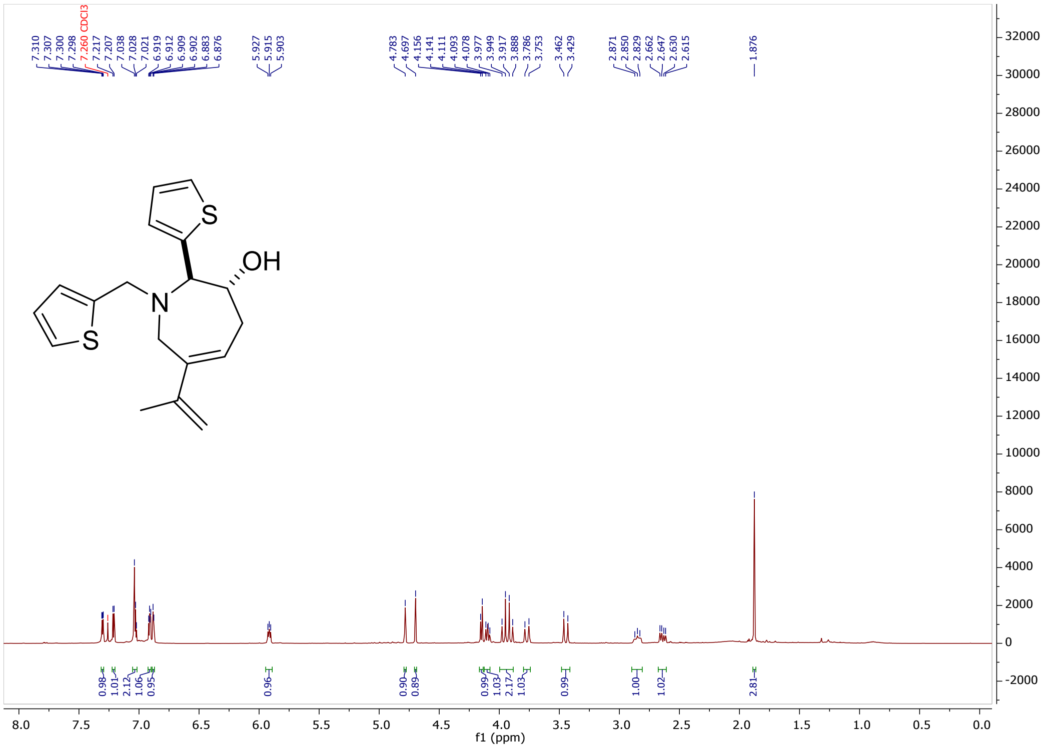

Figure S67. ^{13}C NMR (126 MHz, $CDCl_3$) of **8d**

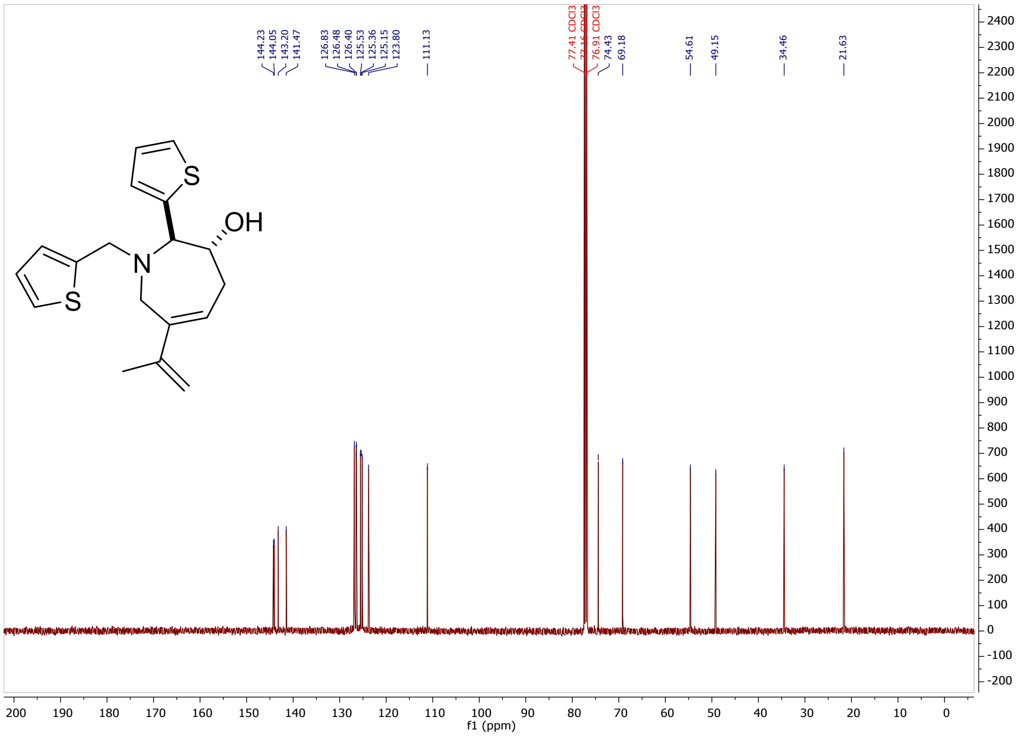

Figure S68. ^{1}H NMR (500 MHz, $CDCl_3$) of **9a**

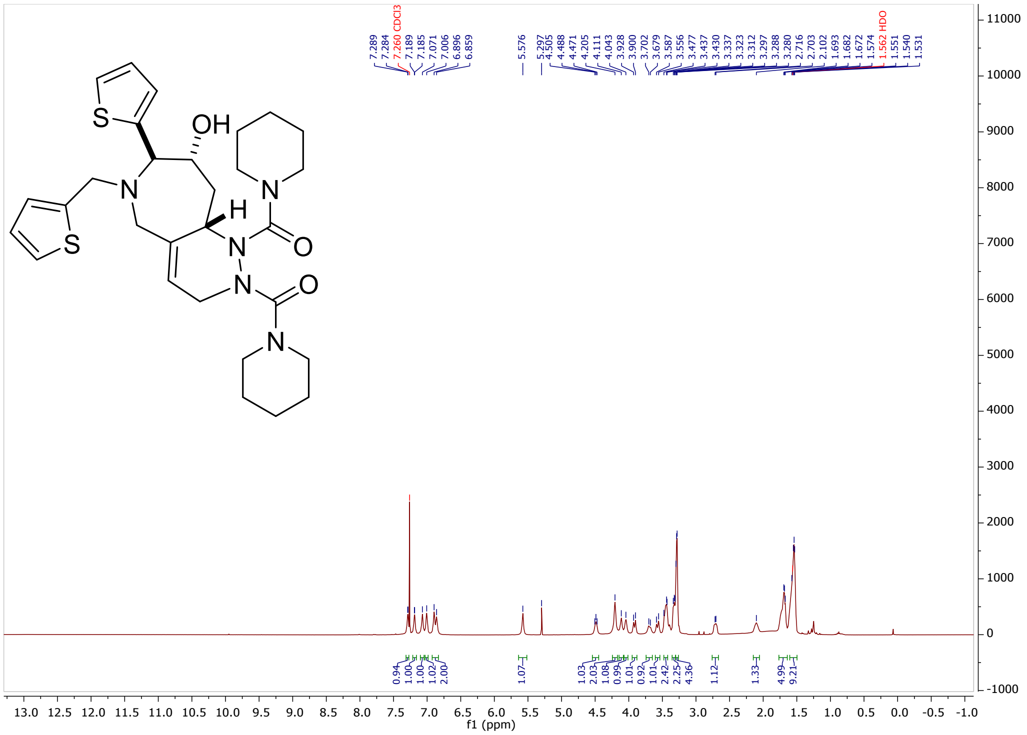

Figure S69. Expanded ^{1}H NMR (500 MHz, $CDCl_3$) of **9a**

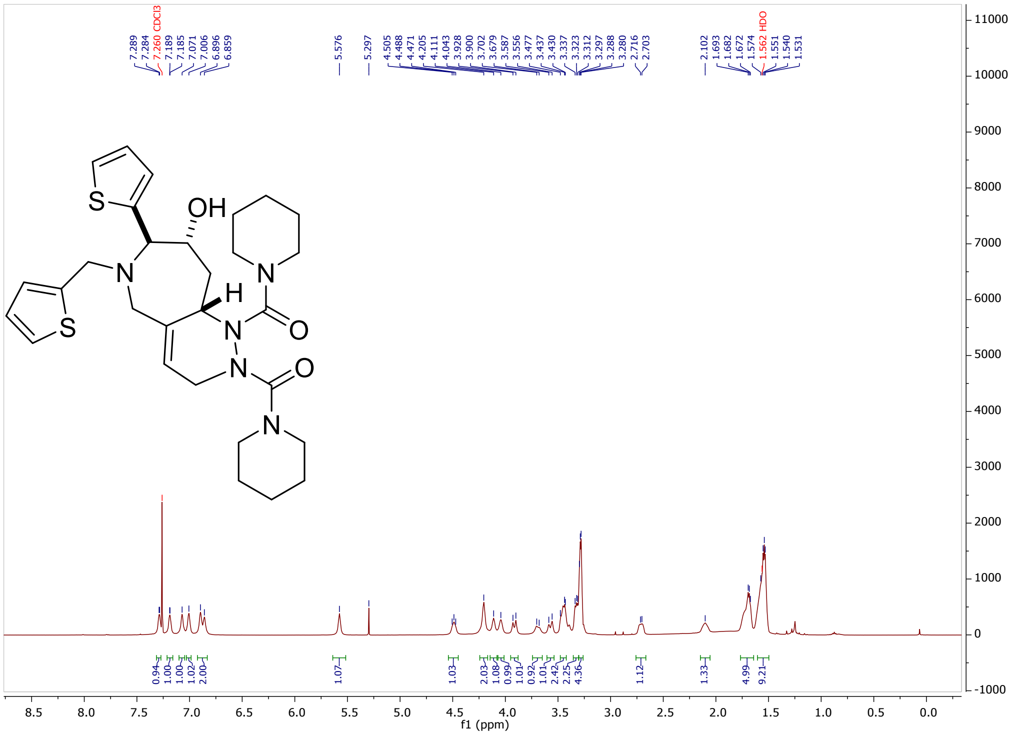

Figure S70. ^{13}C NMR (126 MHz, $CDCl_3$) of **9a**

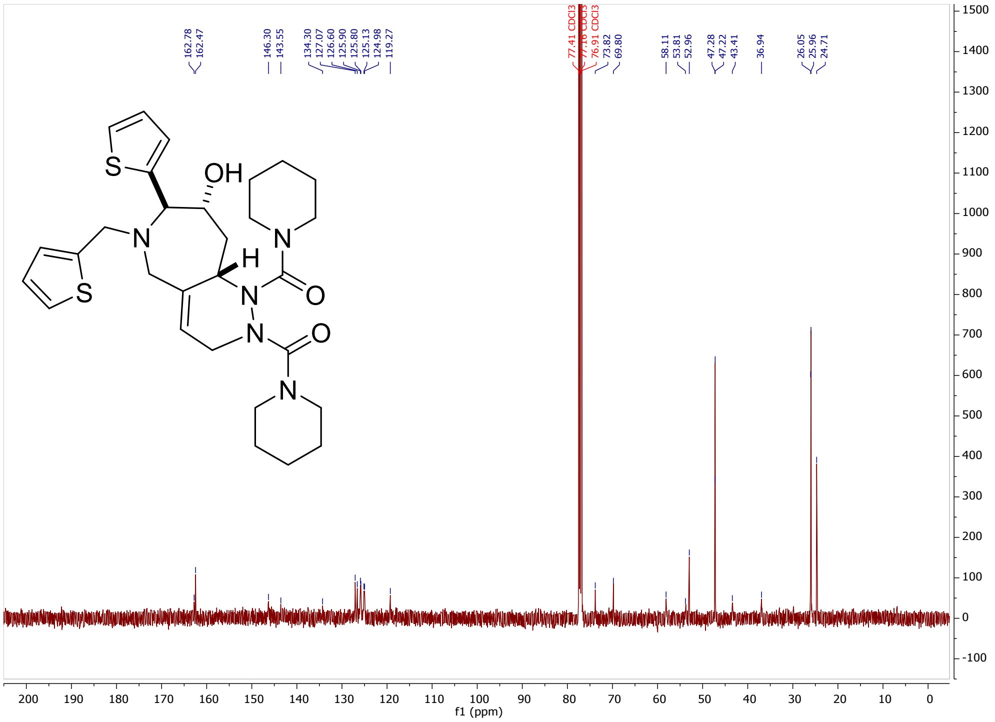

Figure S71. ^{1}H NMR (500 MHz, $CDCl_3$) of **9b**

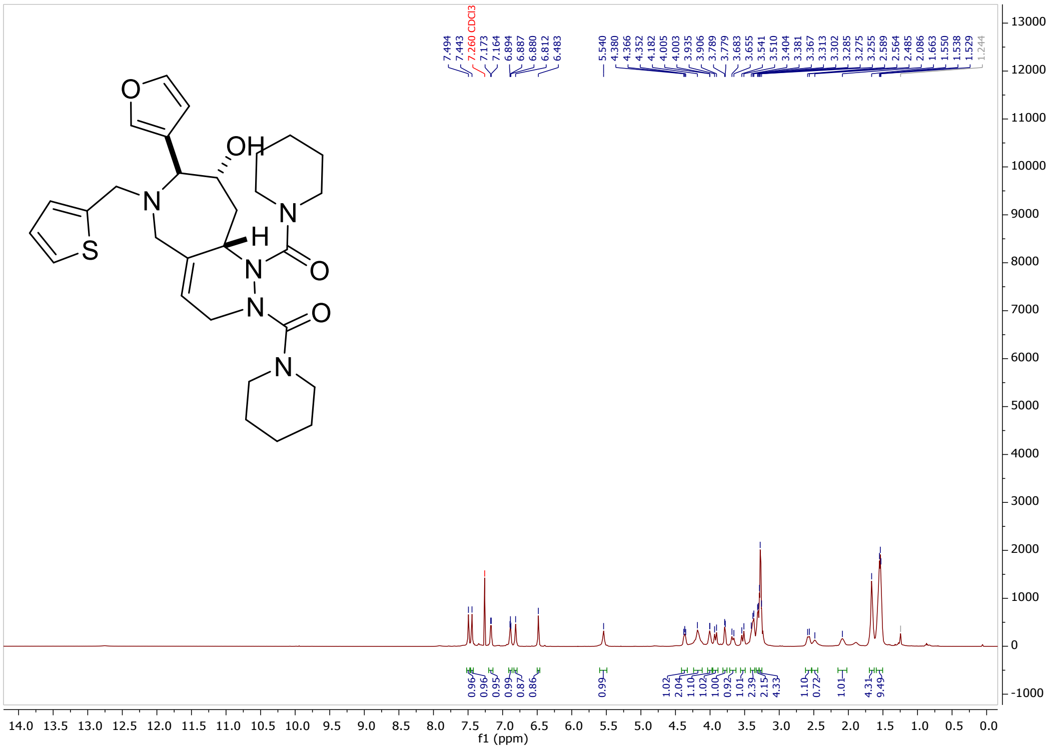

Figure S72. ^{13}C NMR (126 MHz, $CDCl_3$) of **9b**

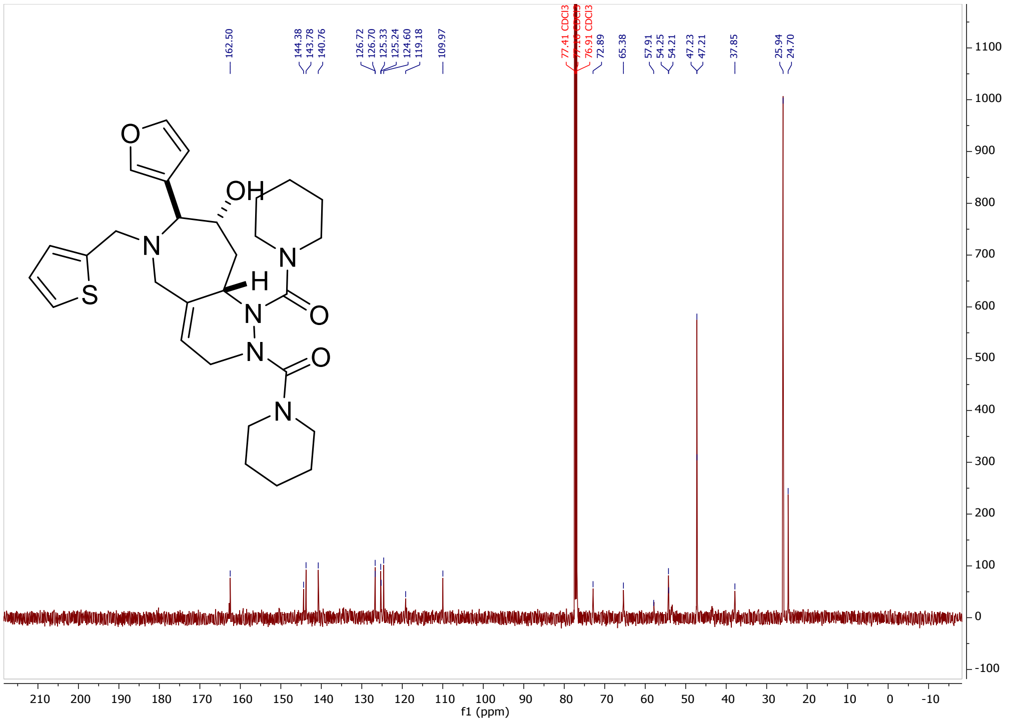

Figure S73. ^{1}H NMR (700 MHz, $CDCl_3$) of **9c**

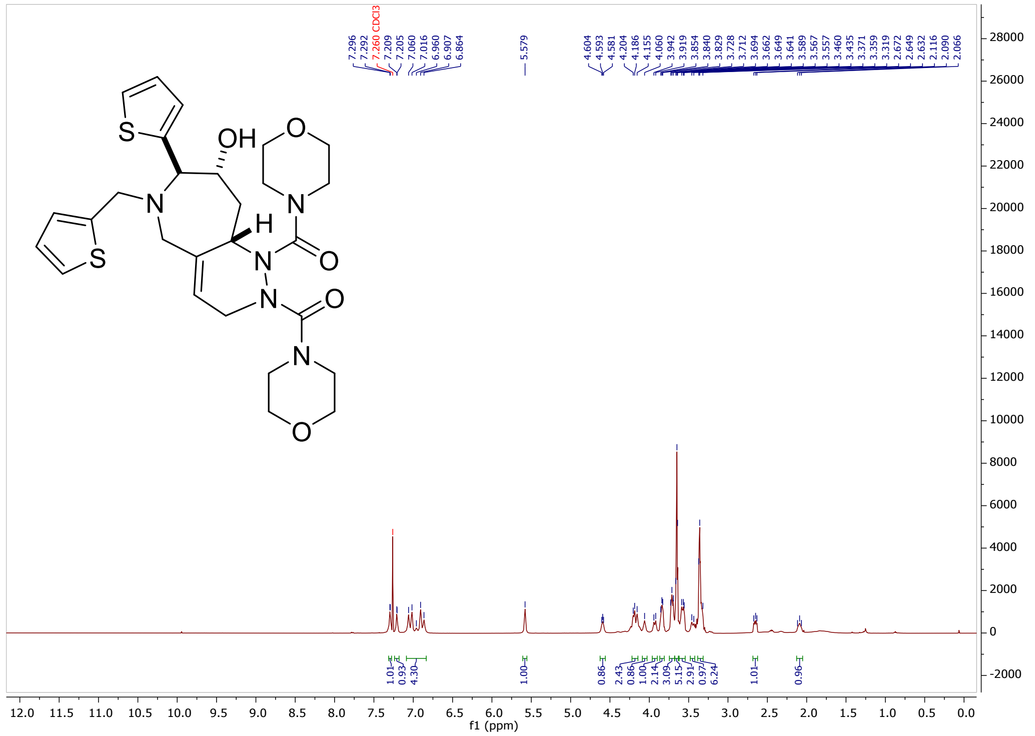

Figure S74. ^{13}C NMR (151 MHz, $CDCl_3$) of **9c**

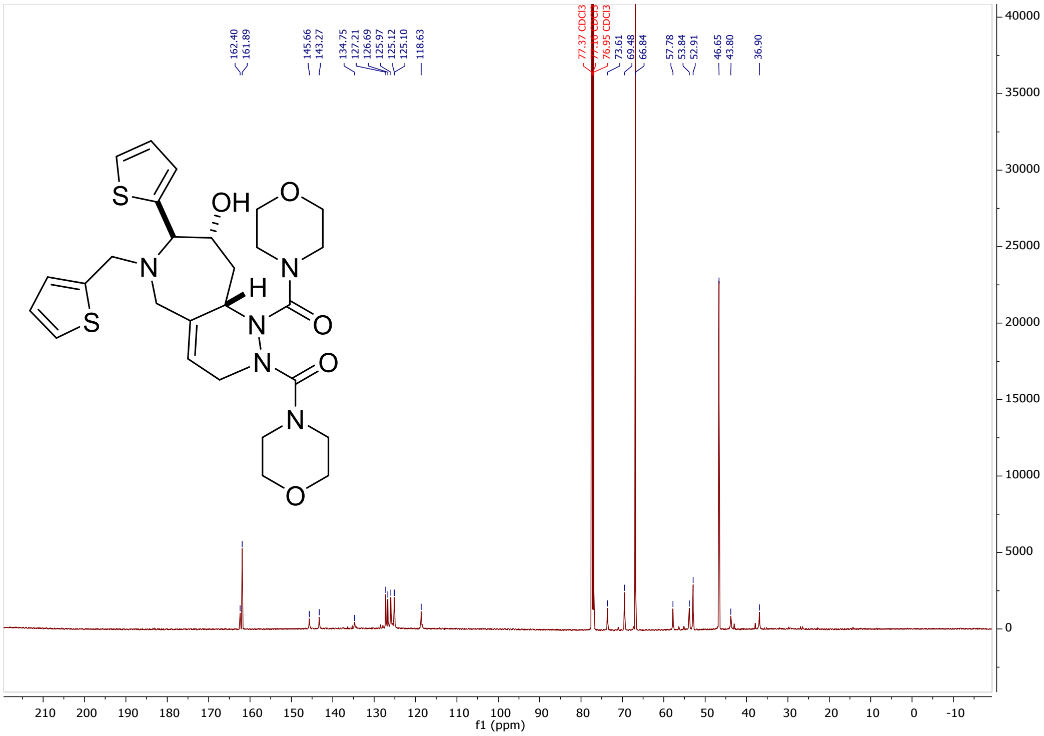

Figure S75. ^{1}H NMR (700 MHz, $CDCl_3$) of **9d**

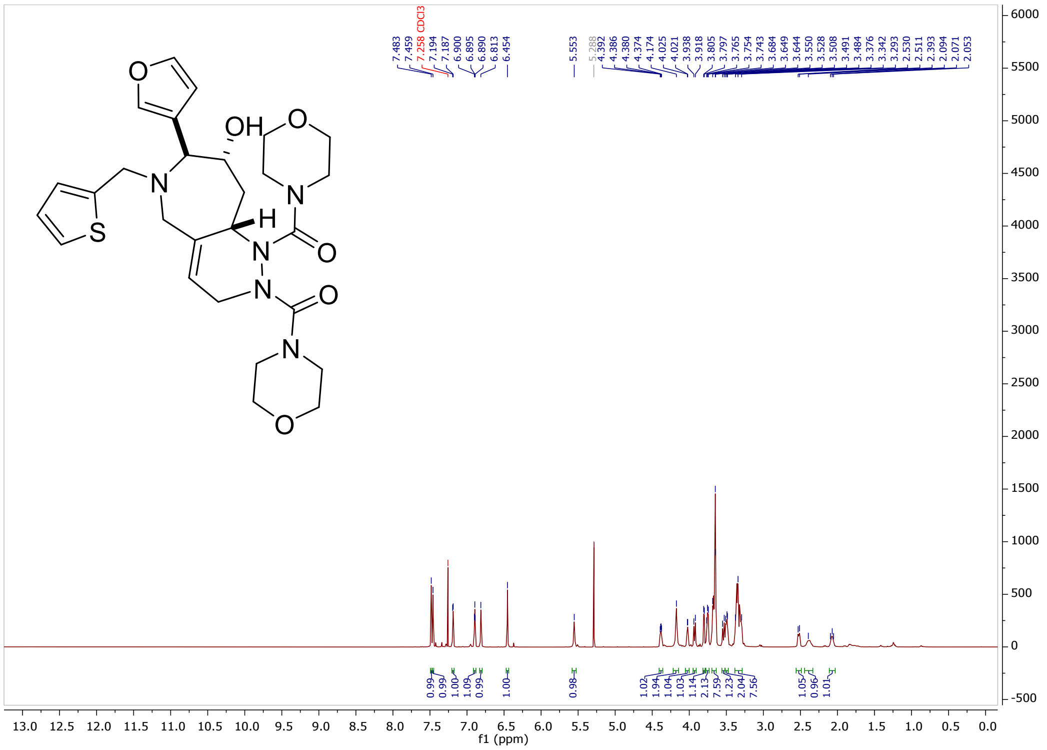

Figure S76. ^{13}C NMR (176 MHz, $CDCl_3$) of **9d**

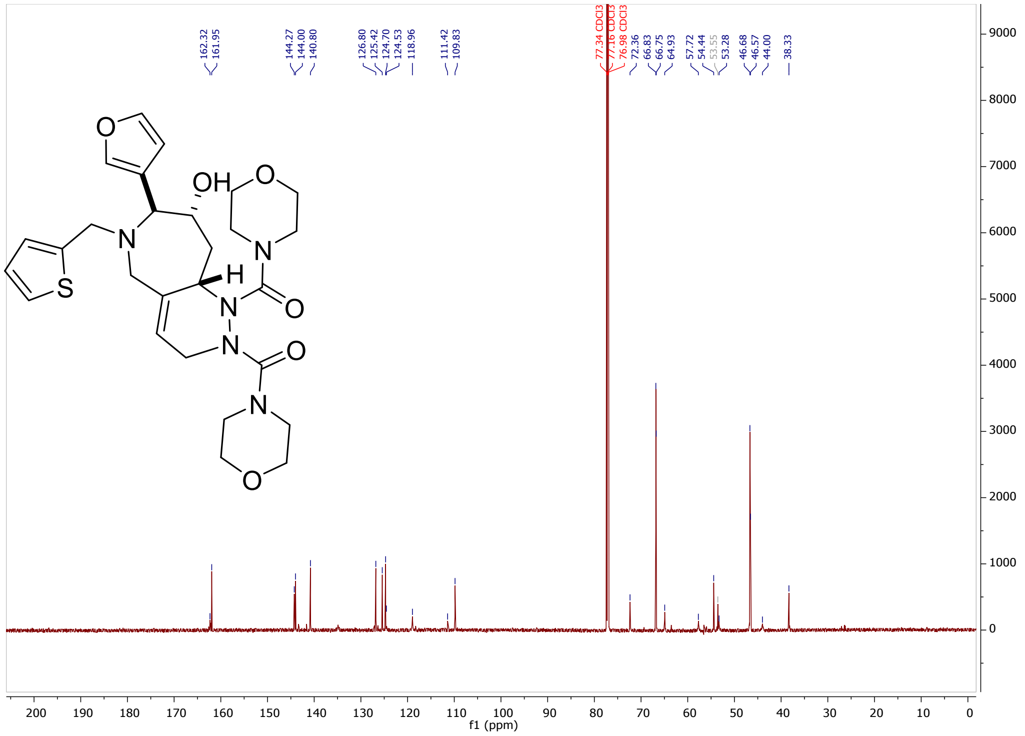

Figure S77. ^{1}H NMR (700 MHz, $CDCl_3$) of **9e**

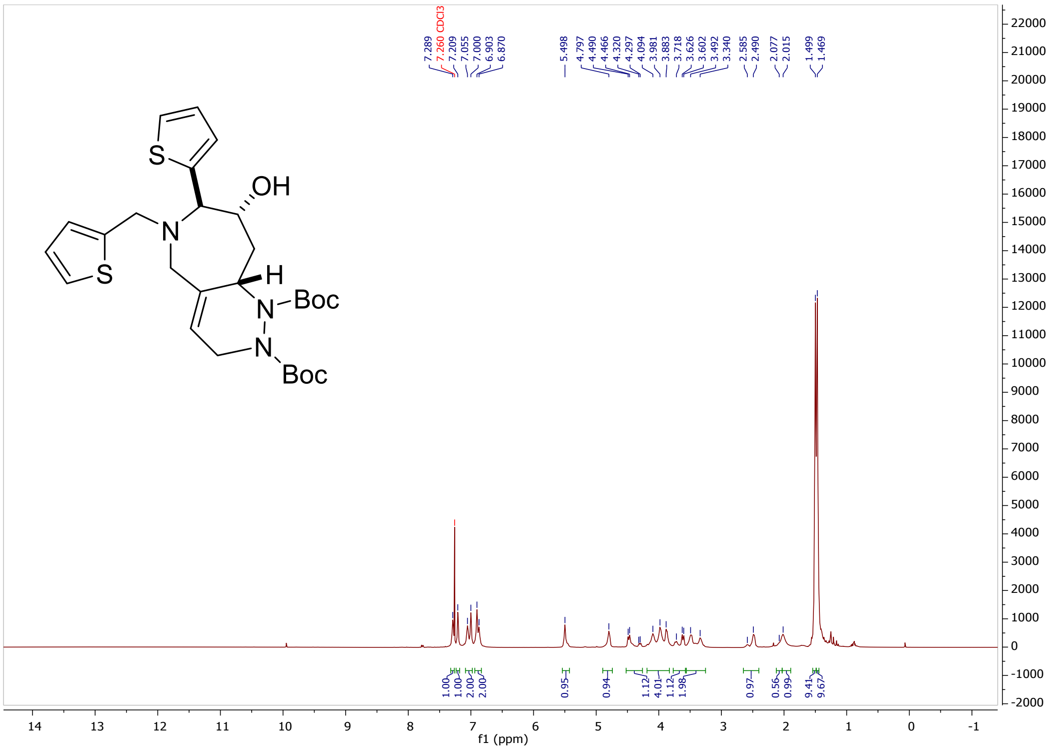

Figure S78. ^{13}C NMR (176 MHz, $CDCl_3$) of **9e**

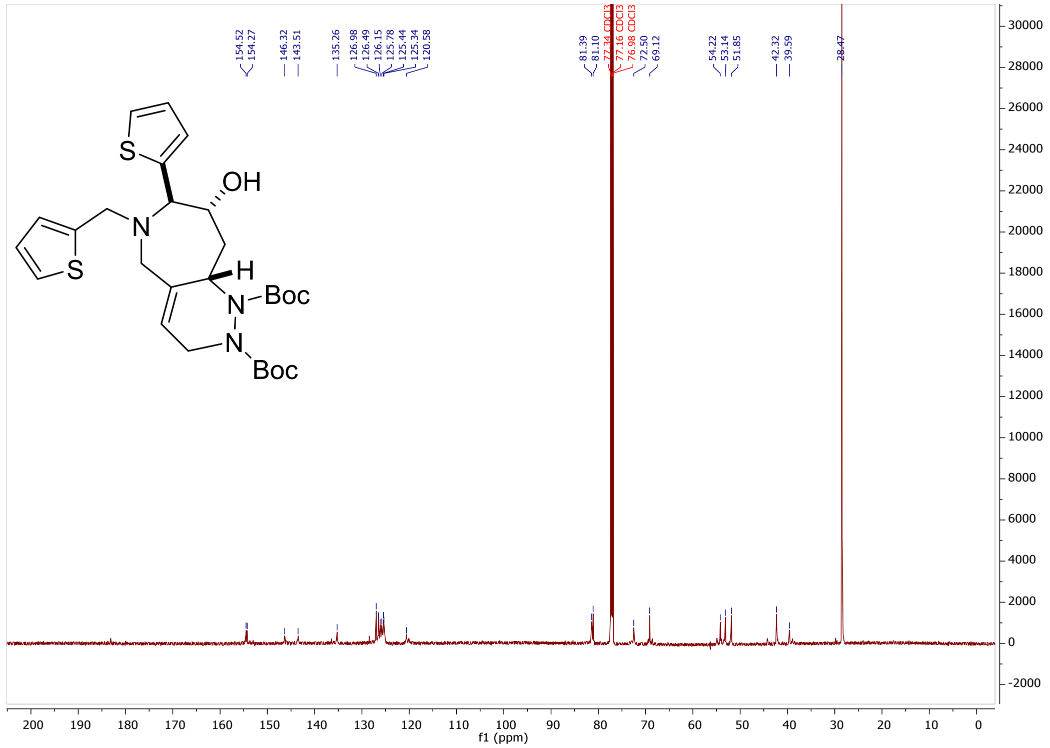

Figure S79. ^{1}H NMR (700 MHz, $CDCl_3$) of **9f**

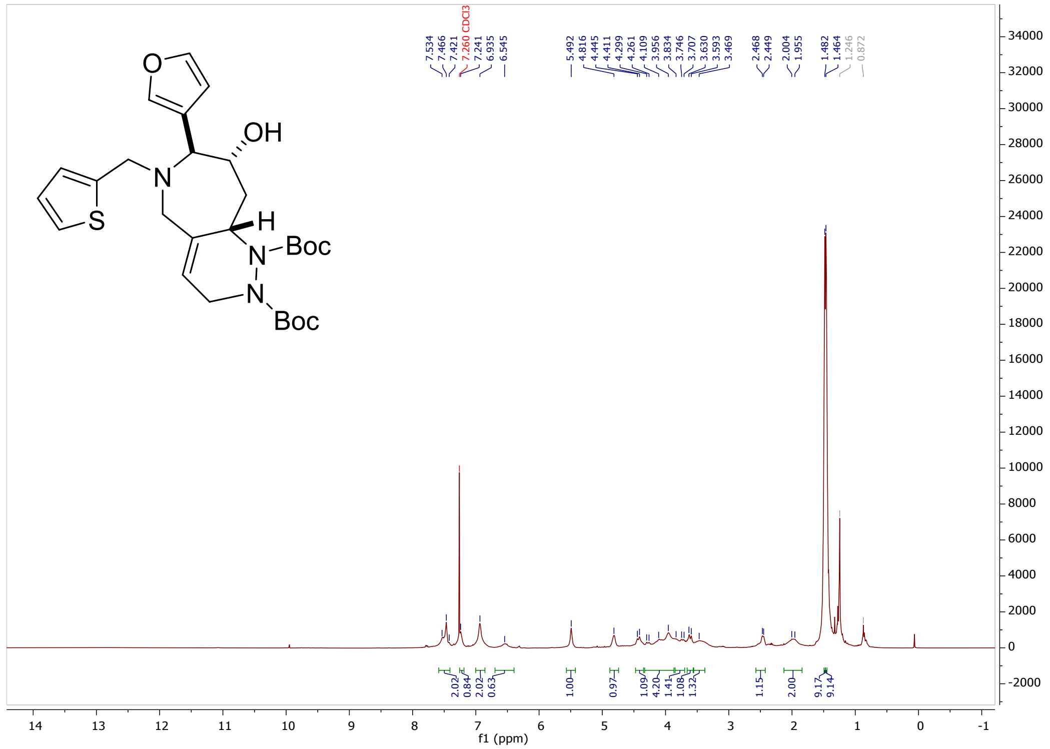

Figure S80. ^{13}C NMR (126 MHz, $CDCl_3$) of **9f**

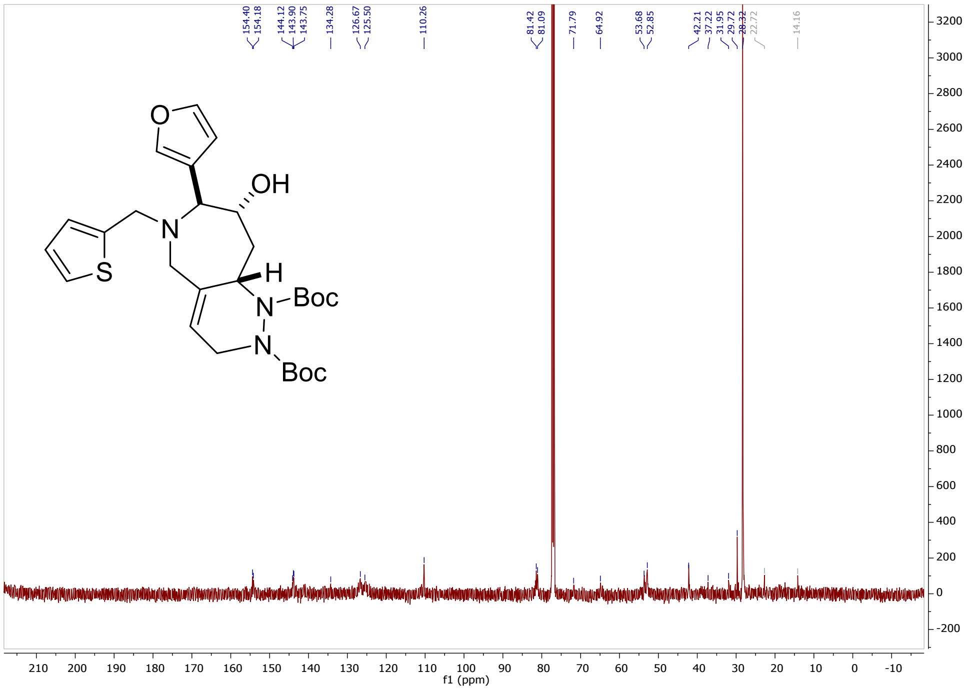

Figure S81. ^{1}H NMR (500 MHz, $CDCl_3$) of **9g**

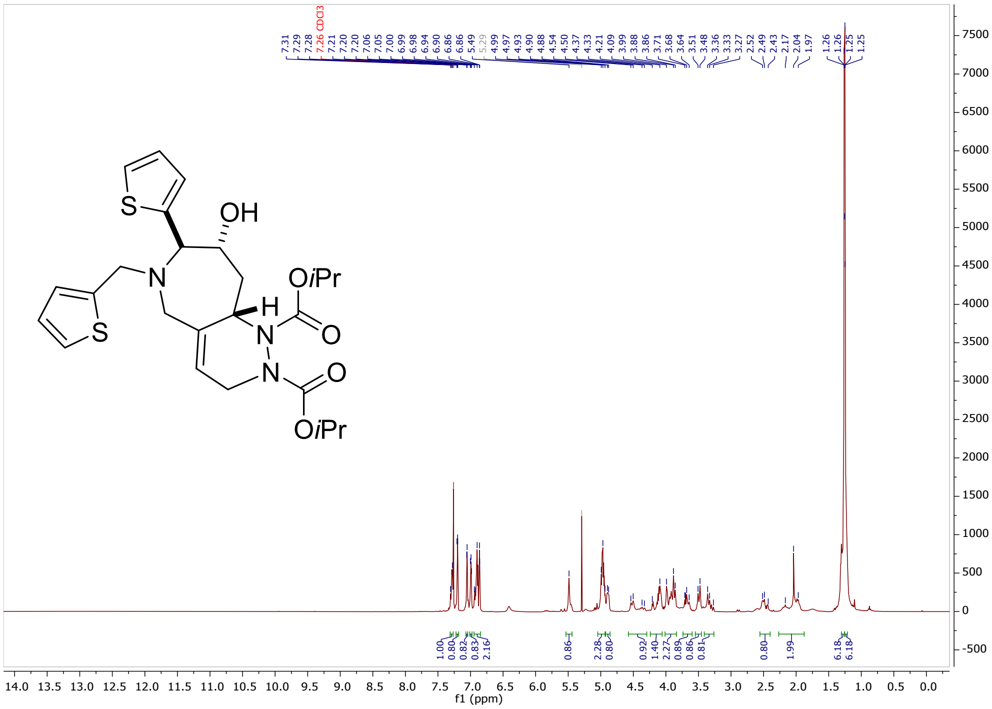

Figure S82. ^{13}C NMR (126 MHz, CDCl3) of **9g**

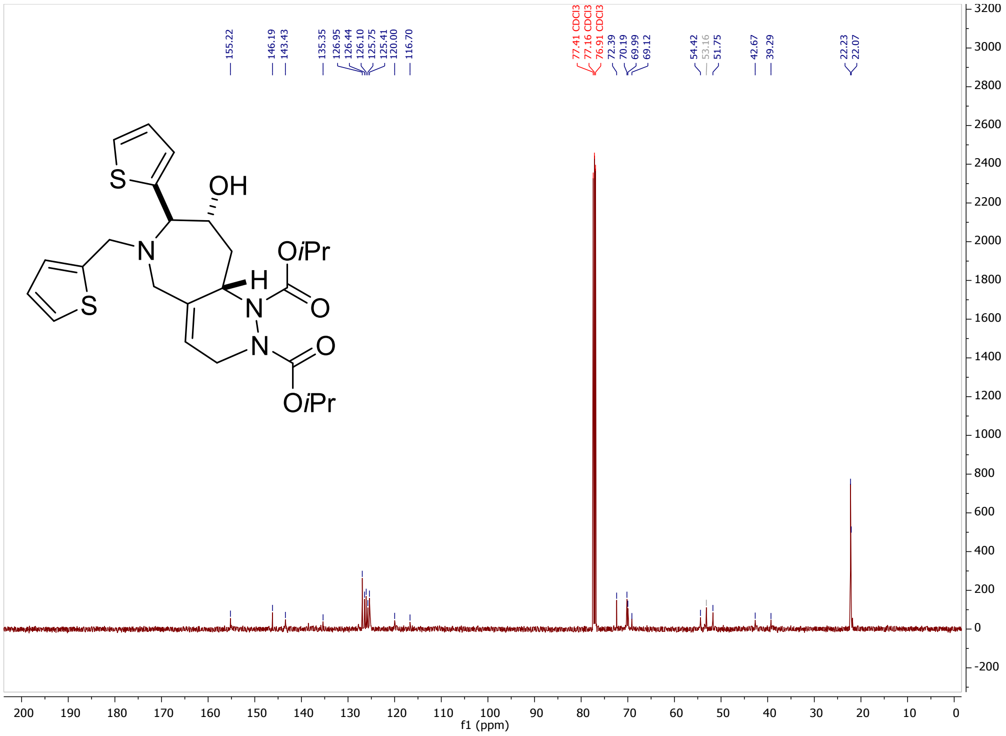

Figure S83. ^{1}H NMR (500 MHz, $CDCl_3$) of **9h**

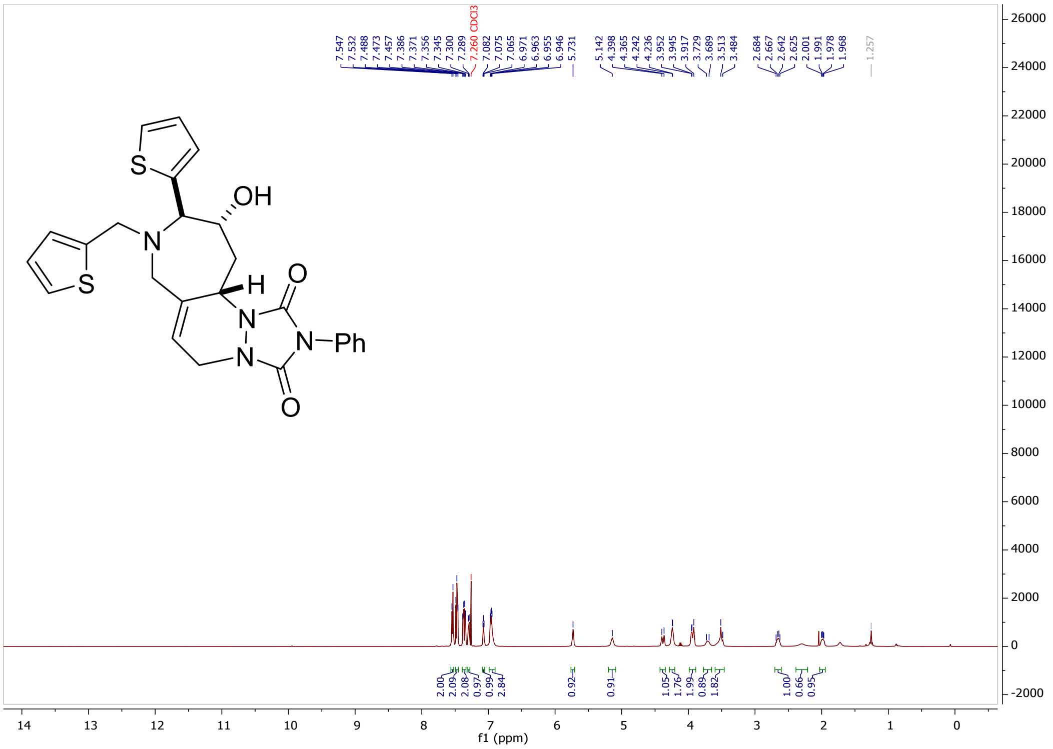

Figure S84. ^{13}C NMR (126 MHz, $CDCl_3$) **9h**

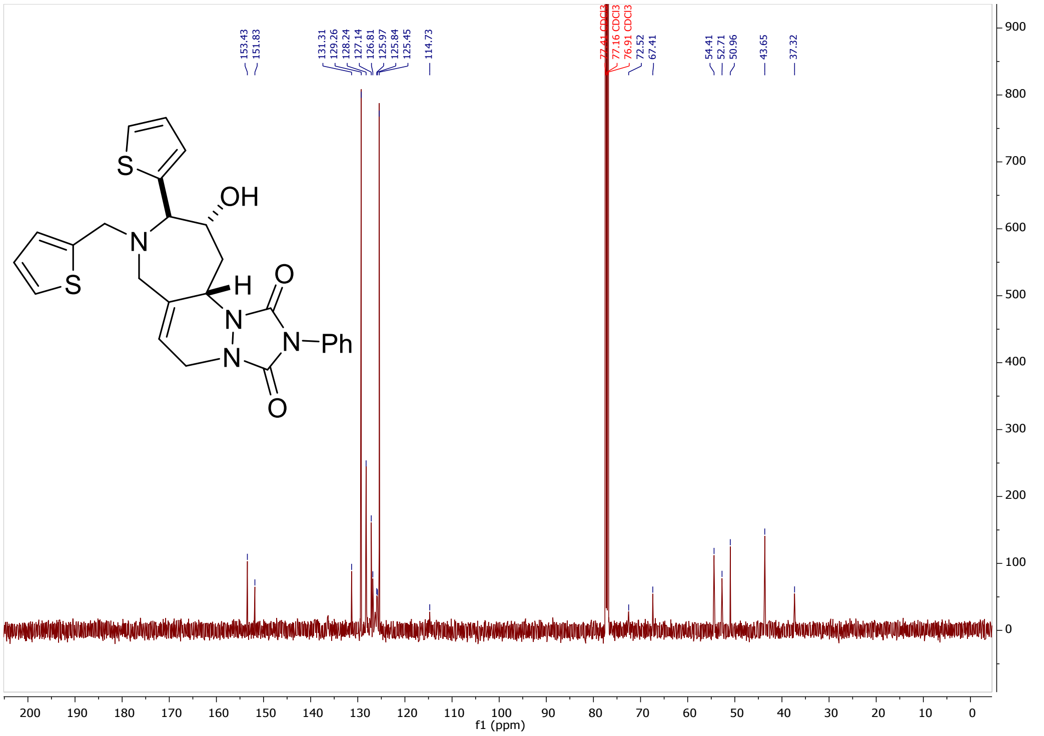

Figure S85. ^{1}H NMR (500 MHz, $CDCl_3$) **9i (**5:1 diastereomeric mixture**)**

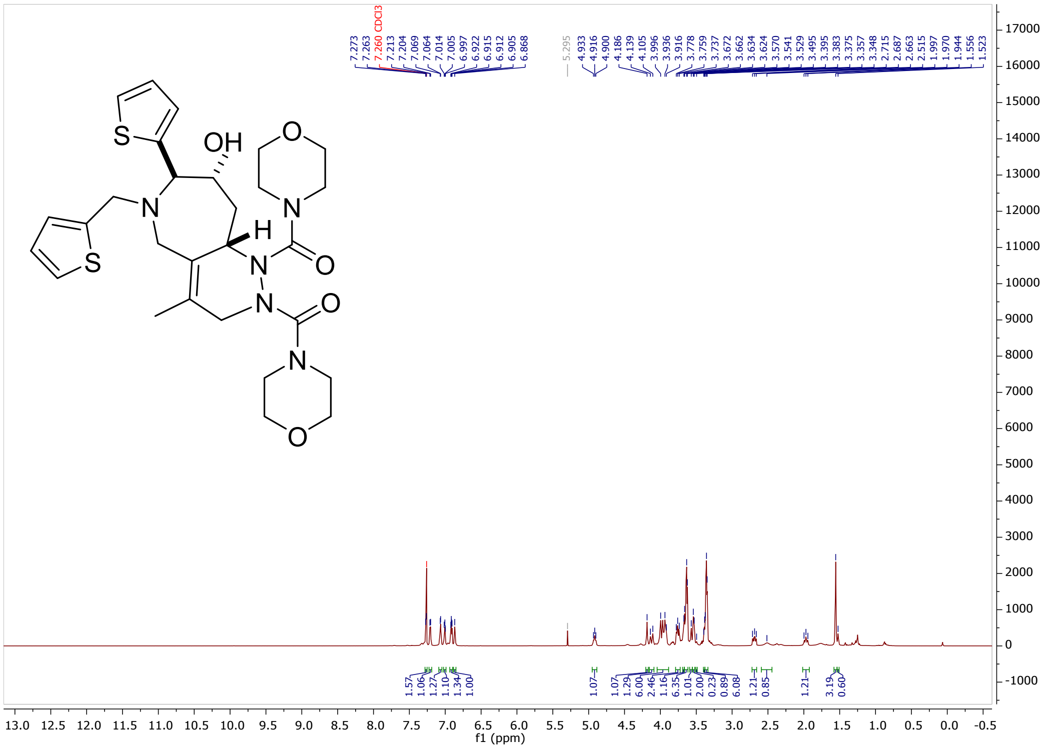

Figure S86. ^{13}C NMR (126 MHz, $CDCl_3$) of **9i (**5:1 diastereomeric mixture**)**

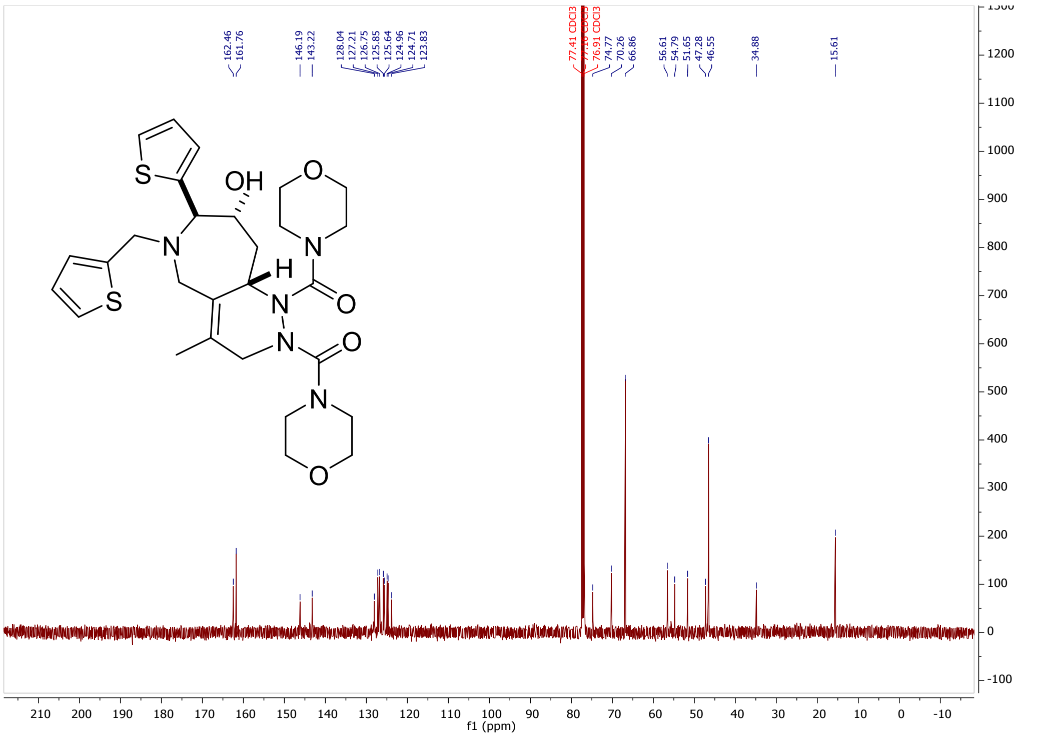

Figure S87. ^{1}H NMR (500 MHz, $CDCl_3$) of **9j**

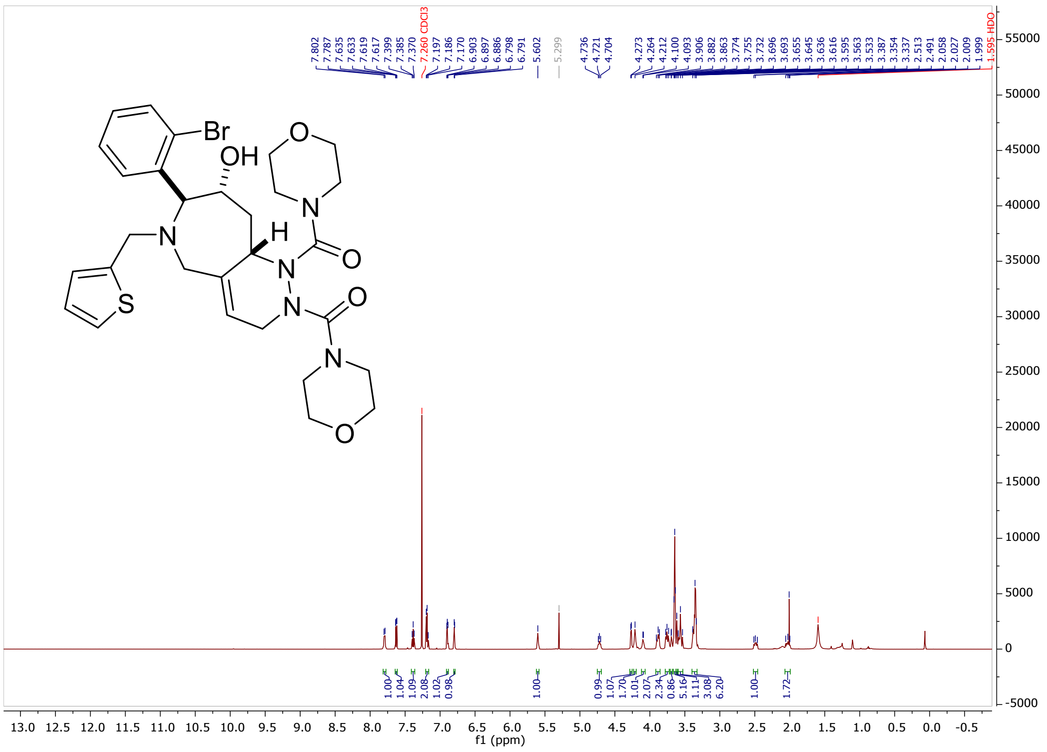

Figure S88. Expanded ^{1}H NMR (500 MHz, $CDCl_3$) of **9j**

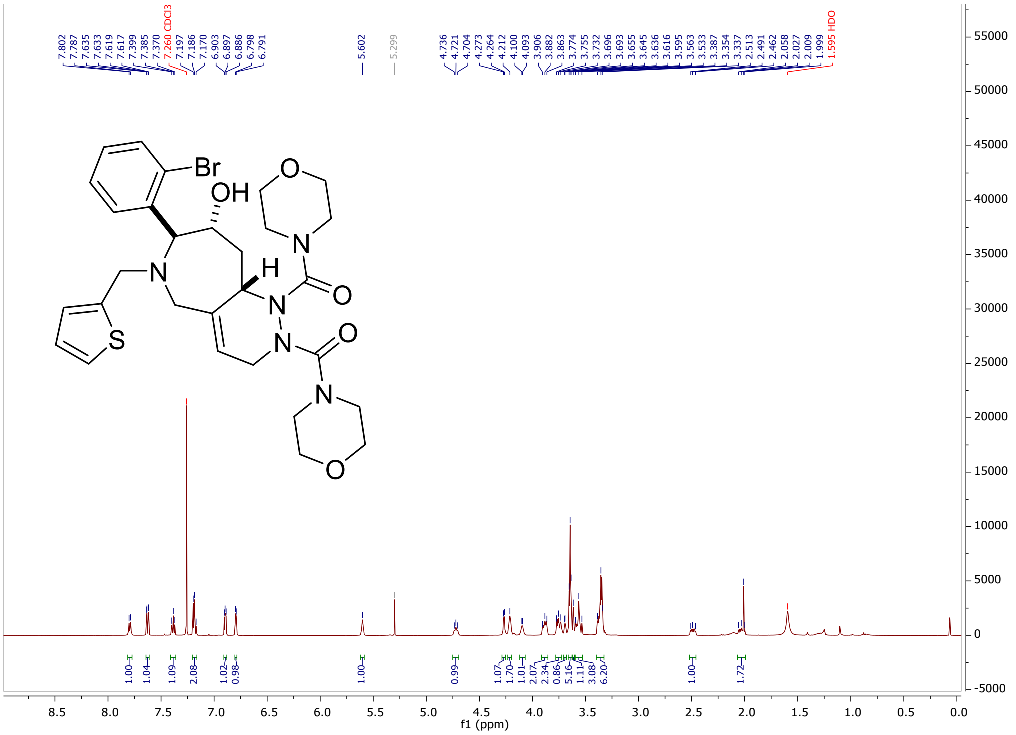

Figure S89. ^{13}C NMR (126 MHz, $CDCl_3$) of **9j**

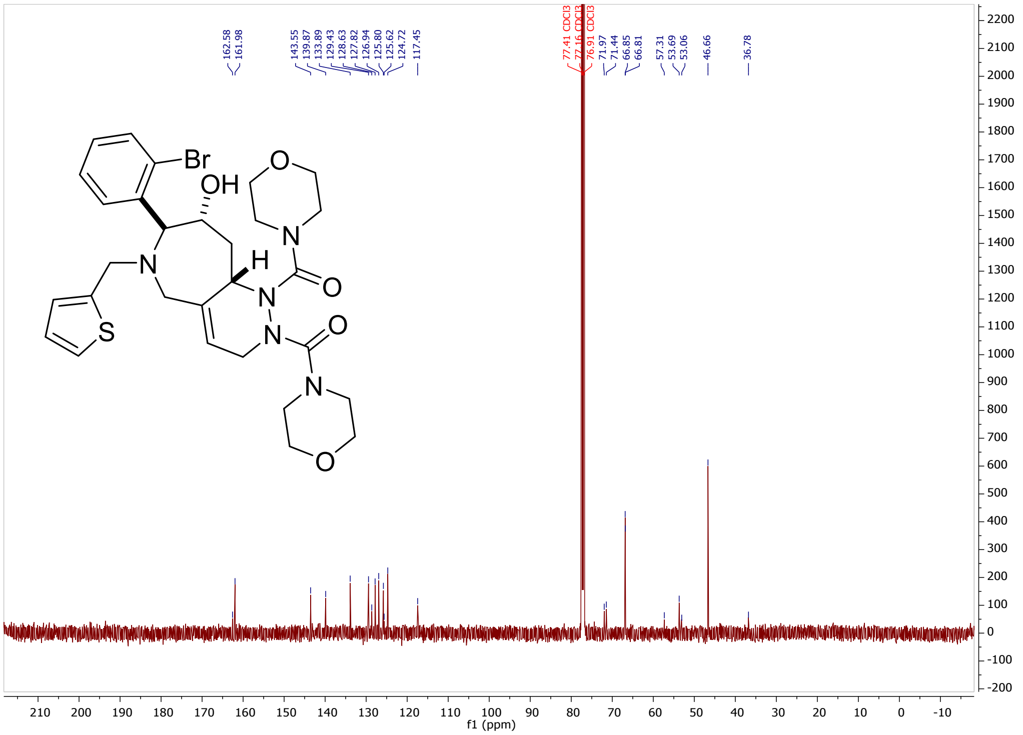

Figure S90. ^{1}H NMR (700 MHz, $CDCl_3$) of **9k**

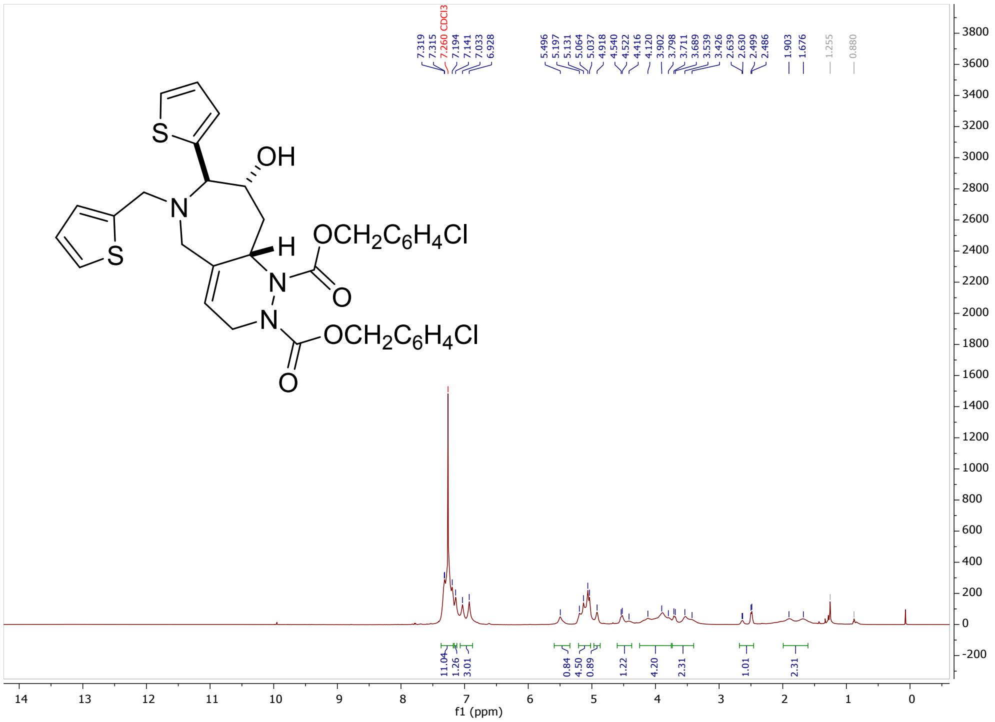

Figure S91. ^{13}C NMR (176 MHz, $CDCl_3$) of **9k**

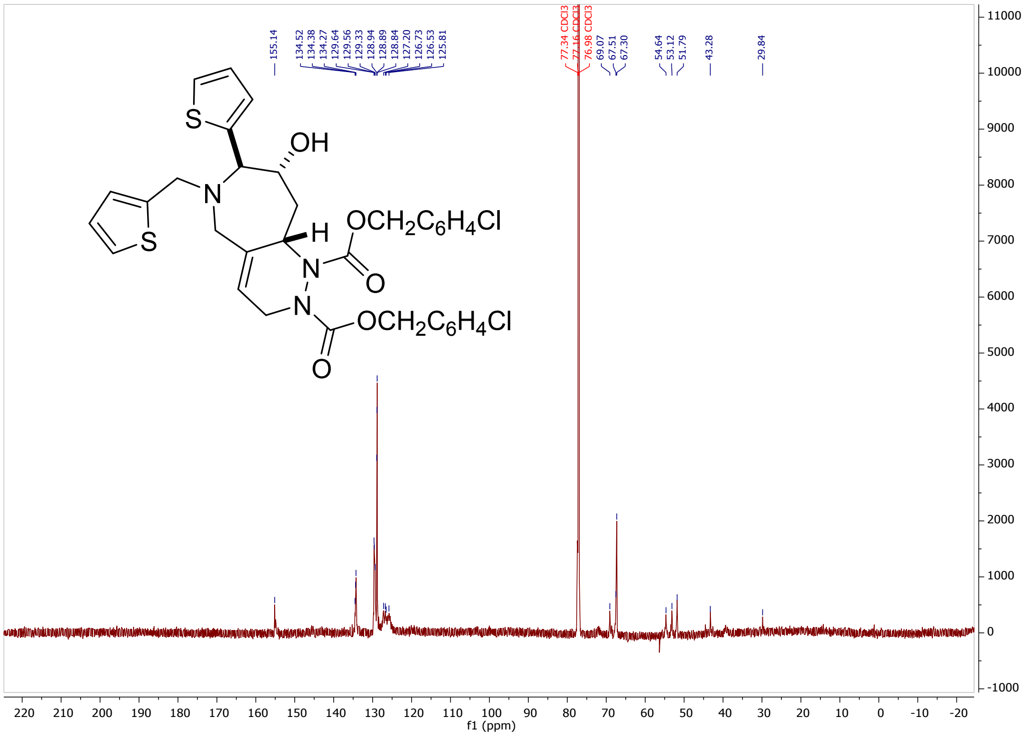

Figure S92. ^{1}H NMR (600 MHz, $CDCl_3$) **9l**

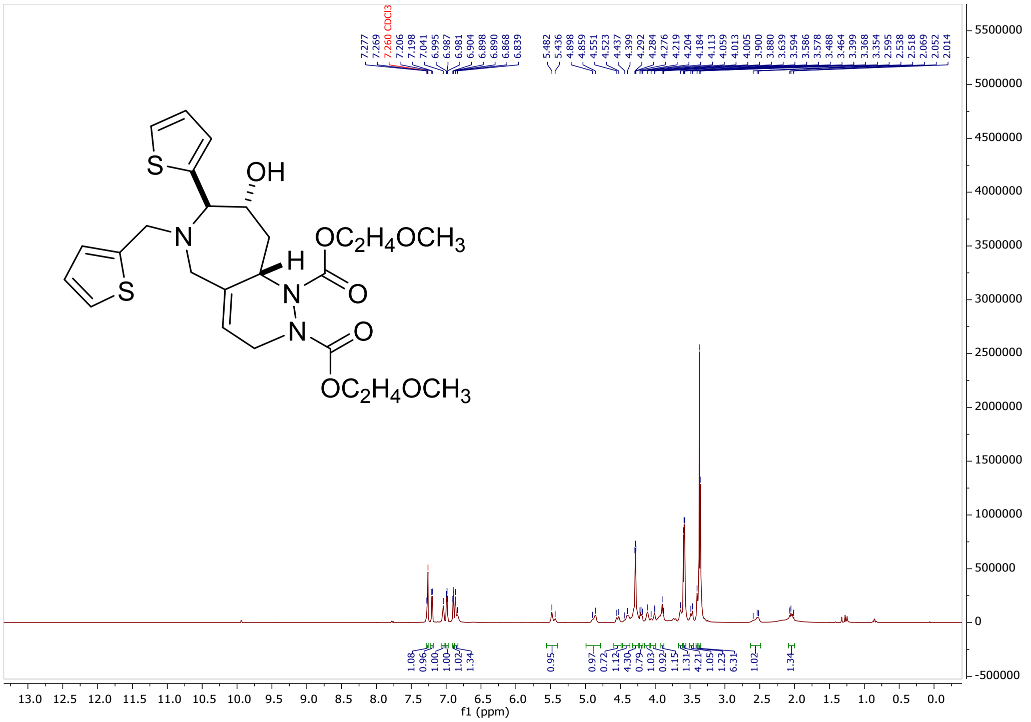

Figure S93. ^{13}C NMR (151 MHz, $CDCl_3$) of **9l**

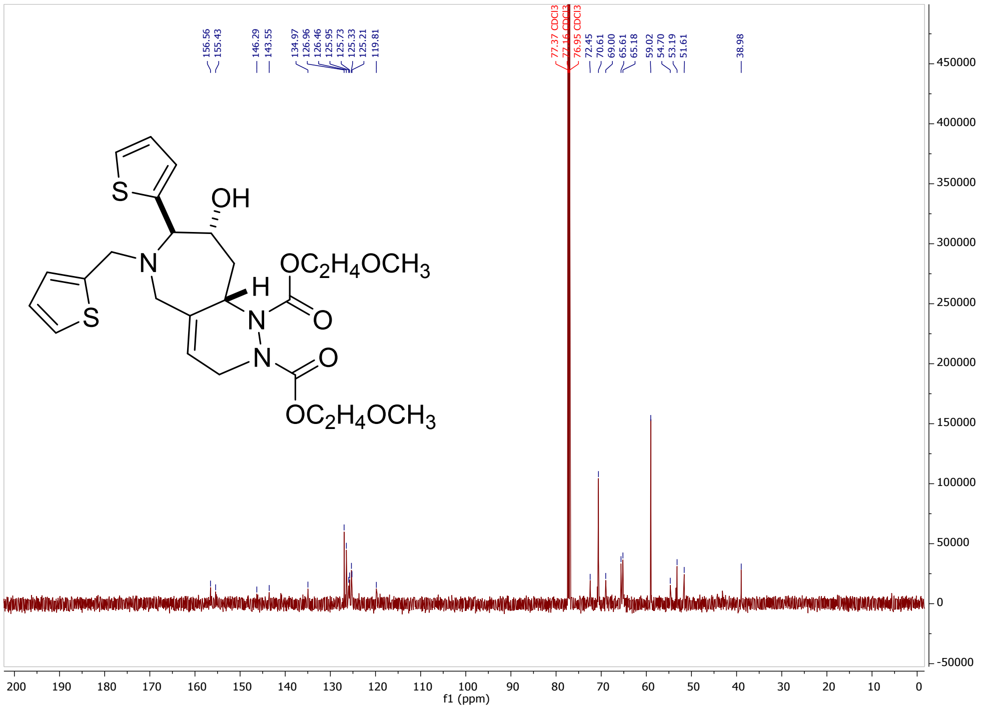

Figure S94. ^{1}H NMR (500 MHz, $CDCl_3$) of **9m**

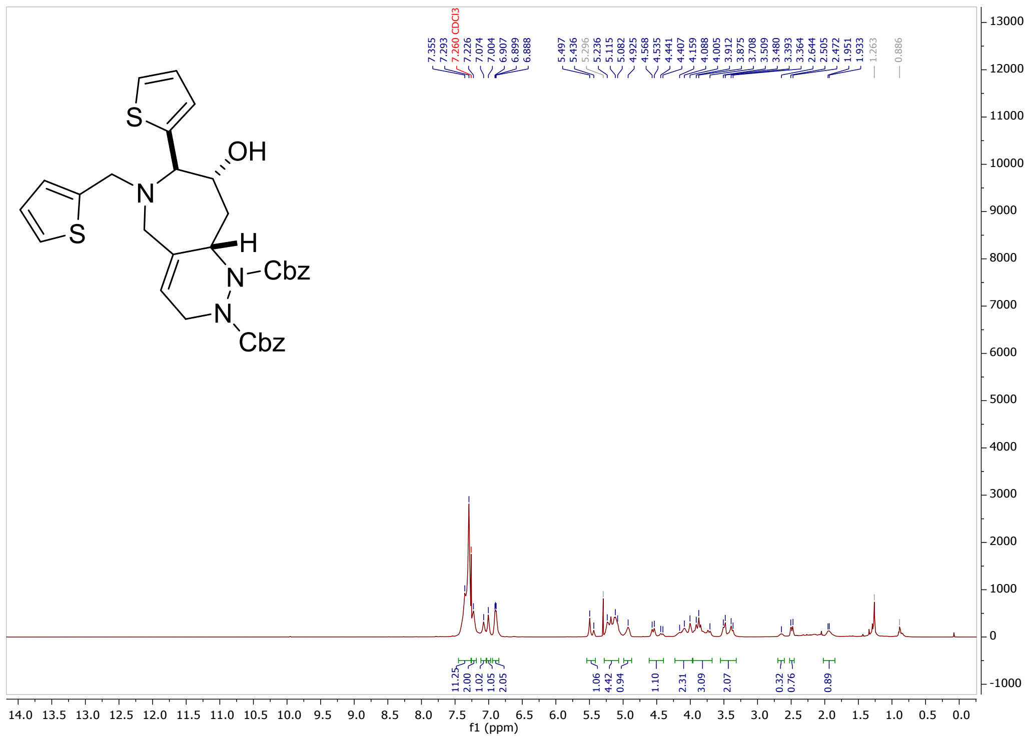

Figure S95. ^{13}C NMR (126 MHz, CDCl3) of **9m**

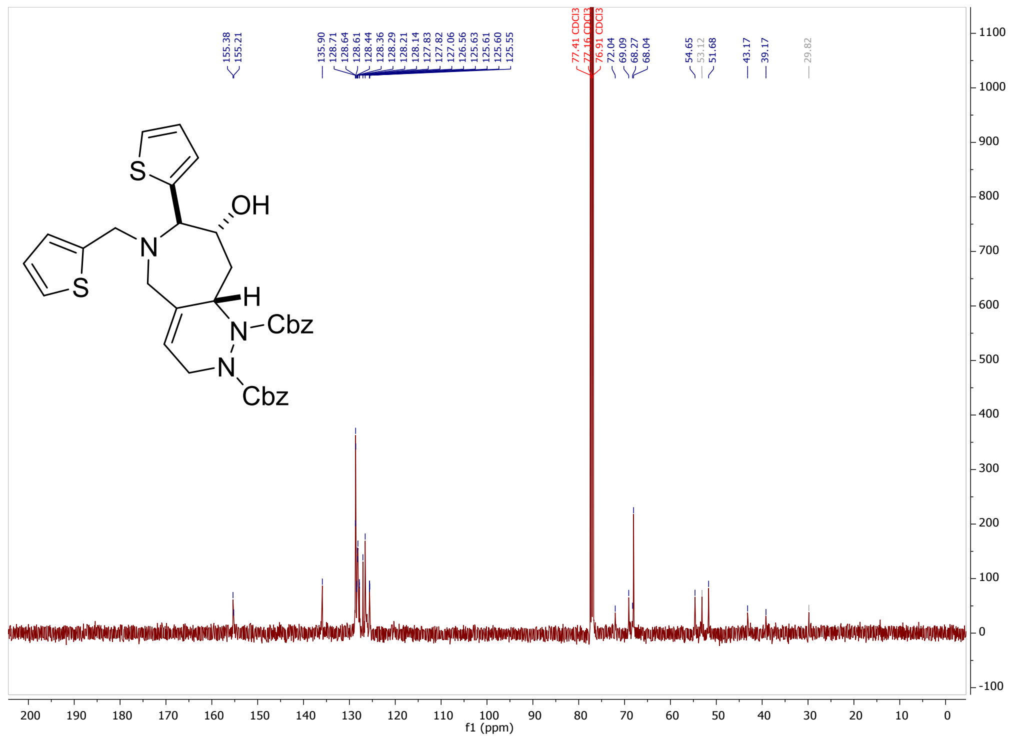

References:

1. Ghelfi, F.; Parsons, Andrew F.; Tommasini, D.; Mucci, A., Intramolecular Diels−Alder Cycloaddition of N-Allyl-N-(2-furylmethyl)amides − First Step of a New Route Towards the Synthesis of a Densely Functionalized Pyrrolizidine Ring. *Eur. J. Org. Chem.* **2001,** *2001* (10), 1845-1852.

2. Ishoey, M.; Petersen, R. G.; Petersen, M. Å.; Wu, P.; Clausen, M. H.; Nielsen, T. E., Diastereoselective synthesis of novel heterocyclic scaffolds through tandem Petasis 3-component/intramolecular Diels–Alder and ROM–RCM reactions. *Chem. Commun.* **2017,** *53* (68), 9410-9413.

3. Molavipordanjani, S.; Emami, S.; Mardanshahi, A.; Amiri, F. T.; Noaparast, Z.; Hosseinimehr, S. J., Novel 99mTc-2-arylimidazo [2, 1-b] benzothiazole derivatives as SPECT imaging agents for amyloid-β plaques. *Eur. J. Med. Chem.* **2019,** *175*, 149-161.

4. Pires, D. E. V.; Blundell, T. L.; Ascher, D. B., pkCSM: Predicting Small-Molecule Pharmacokinetic and Toxicity Properties Using Graph-Based Signatures. *J. Med. Chem.* **2015,** *58* (9), 4066-4072.
